# Sequential Annulations to Interesting Novel Pyrrolo[3,2-*c*]carbazoles

**DOI:** 10.3390/molecules24203802

**Published:** 2019-10-22

**Authors:** Alice Benzi, Lara Bianchi, Massimo Maccagno, Angela Pagano, Giovanni Petrillo, Cinzia Tavani

**Affiliations:** Department of Chemistry and Industrial Chemistry, Università di Genova, Via Dodecaneso 31, I-16146 Genova, Italy; alice.benzi@edu.unige.it (A.B.); lara.bianchi@unige.it (L.B.); massimo.maccagno@chimica.unige.it (M.M.); angela_pagano@alice.it (A.P.); giovanni.petrillo@unige.it (G.P.)

**Keywords:** nitroindoles, nitrobutadienes, benzannulation, Michael addition, Cadogan reaction

## Abstract

Herein we report a significant, valuable extension of a recently implemented pyrrole benzannulation methodology that, employing versatile nitrodienes from our lab as useful C_4_ building blocks, led to indole derivatives characterized by unusual patterns of substitution. The 6-nitro-7-arylindoles resulting from suitably derivatized, non-symmetric dienes are of foreseeable synthetic interest in search for new polyheterocyclic systems. As an example, pyrrolocarbazoles with a rarely reported ring fusion were synthesized with the classical Cadogan protocol. Furthermore, the proven easy reducibility of the nitro group to amine will surely open the way to further interesting elaborations.

## 1. Introduction

Due to the outstanding importance of indole derivatives in many applicative fields—as natural products, pharmaceuticals, fine chemicals and materials, as well as core structures in more complex heterocyclic systems—their synthesis [1,2,3] keeps on being a very active research subject. Among the plethora of more or less recent methods, benzannulation has been surely less practiced with respect to heteroannulation. Actually, classic syntheses tend to build up the penta-atomic heteroring, exploiting the functionalities of properly derivatized benzene substrates [4], rather than assembling the hexa-atomic ring onto a pyrrolic starting material [4,5,6]. Only in recent years, some examples of benzannulating procedures in which a C_4_ building block is involved have been reported: an approach to be generally preferred when specific substituents are needed at the 4–7 positions, as they can be conveniently placed in the appropriate positions of the employed reagent. Examples of pericyclic processes are limited in number, including classic Diels-Alder reactions of electron-poor dienophiles like nitropyrroles [7], or hetero-Diels-Alder reactions of electron-rich *N*-methylpyrroles with 4,5-dicyanopyridazine [8]; more frequently, ionic processes have been reported, where various sources of the C_4_ fragment were used, combined with the appropriate pyrrole derivative. Beside traditional double electrophiles like 4-chloro-4-arylthiobutanoate [9], more recently, 2-butoxy-2,3-dihydrofurans [10], or γ-carbonyl-*tert*-butylperoxides [11] were employed; other methods rely on the efficiency of transition metal catalysis [12,13,14,15]. It should be also mentioned that the benzene ring of the final indole can be also built up starting from pyrrolic intermediates that already contain all, or some, of the carbon atoms of the eventual new ring.

**Scheme 1:**
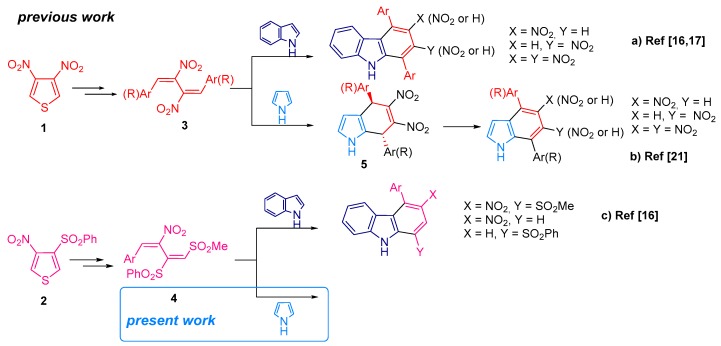
Pyrrole and indole benzannulation reactions.

In this context it should be placed our recent work on the benzannulation of indole [16,17], employing versatile nitrobutadienic building blocks (**3**, **4** in Scheme 1, parts *a* and *c*, respectively) obtained from the initial ring opening of suitably substituted nitrothiophenes (**1**, **2**) [18,19,20]. Thus, mixtures of 2- and 3-nitro-, or 2,3-dinitro-1,4-diarylcarbazoles were obtained from indole by action of 2,3-dinitrobutadienes **3**, while when employing mononitrobutadienes **4**, 4-aryl-3-nitrocarbazoles were generated as the main products.

It should be said that the practical interest of such benzannulation processes was somehow diminished by their modest selectivity, leading in every case to mixtures of compounds. Interestingly enough, when we applied the same procedure on pyrrole [21] with the 2,3-dinitrobutadienes **3**, the method’s outcomes were more gratifying from both the mechanistic and the synthetic point of view, thanks to the unexpected interception of a key intermediate (**5**, Scheme 1, part *b*), that allowed a definitely better selectivity to the whole process: actually, from the isolated **5**, suitable procedures were optimized to generate either mononitro- or dinitro-indoles substituted in positions that are not easily accessible, for instance, by means of S_E_Ar reactions.

Such interesting results encouraged us to proceed in that research by adding another tile to the picture. We present here, our very recent results obtained in the benzannulation of pyrrole with the non-symmetric 2-nitro-1,3-dienes **4,** which opens the door to possible further elaborations, aimed at building-up new polyheterocyclic derivatives.

## 2. Results

### 2.1. Search for the Best Experimental Conditions

**Scheme 2:**
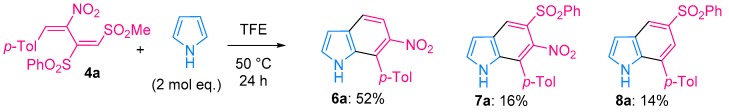
Results from the reaction between the 2-nitrodiene **4a** and pyrrole at 50 °C in TFE.

We started our research applying to the model substrate **4a** (Ar = *p*-Tol) the same reaction conditions previously applied [21] to diene **3** (2 mol equiv of pyrrole, TFE, 50 °C). Here again, 24 h were necessary to observe, by TLC, the complete disappearance of **4a**; the analysis of the chromatographed final reaction mixture highlighted the formation of three different indole derivatives (**6a**–**8a**, Scheme 2), **6a** being by far the prevalent one (Table 1, entry 1). We considered satisfying the balance and good the selectivity, in favor of the derivative probably most appealing thanks to the preservation of the versatile nitro group.

Increasing the molar equivalents of pyrrole from 2 to 4, we obtained a faster reaction (Table 1, entry 2) with almost unchanged results. A gradual decrease of the temperature caused a progressive slowdown (entries 3 and 4), maintaining again almost unchanged absolute and relative yields. It should be noted that neither at 30 °C nor at 0 °C it was possible to observe and isolate an unsaturated intermediate analogous to the dihydroindole **5** of Scheme 1. At T = 0 °C no change in the reaction mixture occurred within 48 h, and the unreacted substrate was quantitatively recovered.

At the end of this screening, we judged those of entry 3 as the conditions of choice, as they allow a reasonable reaction time and a better yield of **6a**.

### 2.2. Verifying the Scope

We proceeded, then, to verify the scope of the reaction, applying the same conditions to a series of 2-nitro-1,3-butadienes substituted at position 1 with different Ar groups. Results are reported in Table 2. It can be observed that the good selectivity and the satisfying balance evidenced for **4a** were not exhibited by the whole series, being maintained only when Ar = Ph, *m*-ClPh, and, somewhat less so, *p*-MeO- and *p*-Cl-Ph, and 1-naphthyl. Particularly negative was the result from **4f**, while for **4g** a fourth product, **9g**, originated in a yield comparable to **6g**. A longer reaction time was required by **4i**, and after 7 h the control TLC still showed traces of substrate. The data obtained until now seem to definitely reveal a non-negligible effect, in particular on the yield of **6**, exerted both by EWG and EDG aryl substituents.

### 2.3. A Possible Elaboration of 7-Aryl-6-nitroindole **6** to Pyrrolo[3,2-c]carbazoles

The 7-aryl-6-nitroindoles **6** obtained herein as the principal products appear of sure interest, as they have never been reported so far: evidence of a pattern of substitution not easy to be obtained otherwise. Actually, the position occupied by the nitro group, *ortho* to the aryl substituent and *meta*-like (i.e., not directly conjugated) to the electron-donating pyrrolic nitrogen atom, is promising, e.g., for further elaborations starting with a reductive step of the nitro group itself.

**Scheme 3:**
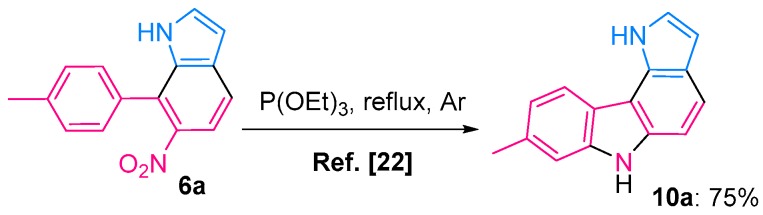
Classic conditions for the Cadogan reaction ([22]) applied to **6a**.

For instance, the application of the classic Cadogan reaction [22] could hopefully build up unusual pyrrolo[3,2-*c*]carbazoles (Scheme 3), very rarely reported before in the literature [23]; that notwithstanding, many other similar scaffolds, different from the kind of fusion between pyrrole and carbazole, have been described. For instance, some pyrrolo[2,3-*a*]carbazoles have been recently synthesized and tested for their pharmacological properties (Pim kinase inhibitory potencies [24]), while the pyrrolo[2,3-*c*]carbazole scaffold characterizes the biologically active natural compounds dictyodendrines [25]. Encouraged by the wide diffusion of fused carbazole scaffolds among natural or synthetic biologically active compounds, we submitted compound **6a** to reflux in triethyl phosphite. The reaction went on smoothly to give the expected compound **10a**, in a 75% yield.

For the extension of the Cadogan reaction to other derivatives, we selected nitroindoles **6a**–**e** and **6h**–**i**, excluding those items for which, because of the poorer results of the benzannulation, the two-step procedure would be inconvenient. The compounds obtained and relative yields are reported in Table 3.

### 2.4. Reduction of the Nitro- to Amino Group in Compounds 6: A First Step to Further Elaborations

**Scheme 4:**
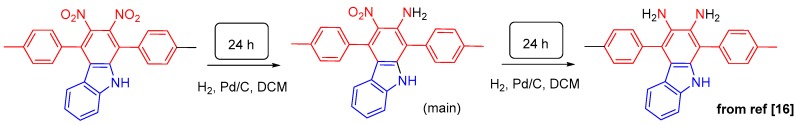
Progressive/regioselective reduction from dinitro- to diamino-carbazole derivatives.

Still in the field of polyheterocyclic synthesis, we envisaged in the reduction of 6-nitro to 6-amino group another potential application for our 7-aryl-6-nitroindoles **6**, in the light of the easy reducibility of the nitro group testified by the results of the Cadogan reaction. In this regard, it should be recalled that in our experience, nitroindoles and nitrocarbazoles are not easily reduced when the nitro groups are positioned on C5 and C3, respectively, as expected when considering the electron-donating effect of the ring nitrogens. Particularly representative of this noteworthy different reducibility was the result [16] obtained from 1,4-diaryl-2,3-dinitrocarbazole (Ar = *p*-Tol, Scheme 4), for which the reduction of the two nitro groups proceeded with such different rates as to reveal a high regioselectivity.

**Scheme 5:**
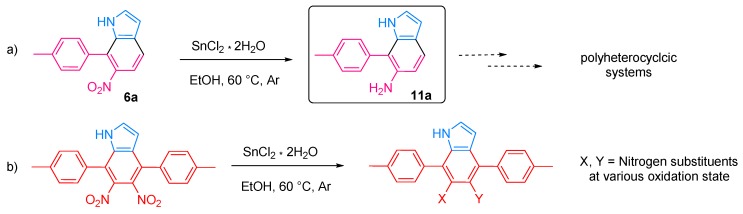
(**a**) Reduction of the nitro group in **6a** to obtain **11a**, a possible precursor for new polyheterocyclic targets.; (**b**) the same reducing agent proves less efficient in the reduction of nitro groups in a 4,7-diaryl-5,6-dinitroindole reported in ref [21].

Beside to the interest in obtaining merely the corresponding amino derivatives, we intend in the future, to investigate their possible utilization to obtain different polyheterocyclic systems (Scheme 5) involving the nucleophilic primary amino group in further reactions. A preliminary test on the model substrate **6a** showed the expected reduction to occur smoothly in satisfactory yield (**11a**: 61% of isolated compound; Scheme 5a) and in a reasonable reaction time (overnight), in conditions (SnCl_2_·2H_2_O, EtOH 60 °C, under Ar) [26] that were not as efficient when previously tried on diaryldinitroindoles (from [21]; Scheme 5b): an encouraging result in the above mentioned perspective of widening the scope of the reduction of **6** towards the synthesis of new *N*-heterocycles.

## 3. Mechanistic Aspect Discussion

As far as the benzannulation reaction is concerned, some considerations can be advanced in order to rationalize the formation and distribution of the observed products, also comparing the present system to the previously studied reaction with indole as a nucleophile (Scheme 1, part *c*). However, it should be specified that, in the absence of any isolable intermediate, the discussion herein has just a speculative significance.

**Scheme 6:**
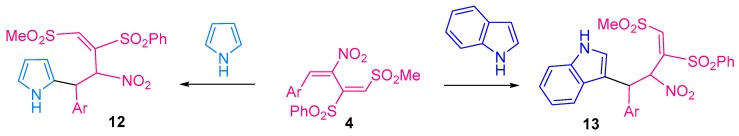
Probable first step of the process under study, and an analogy of the reaction with indole.

As already ascertained in other processes, we found, in recent years [27,28], that the more reactive electrophilic site within **4**-type butadienes is the nitrovinylic moiety, presumably leading, as a first intermediate, to the Michael adduct **12** (Scheme 6), analogous to that (**13**) hypothesized for the reaction with indole [16]. Of course, due to the different site-selectivity of the considered heterocycles, the new bond involves C2 of pyrrole, while in the case of indole involves C3.

**Scheme 7:**
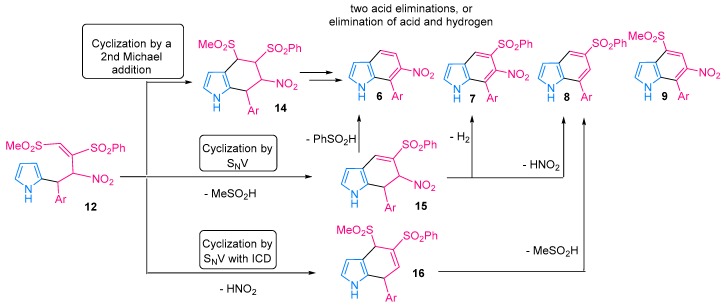
Conceivable evolutions of **13** according to the observed products.

Among the many conceivable evolutions of **12**, it is very difficult to individuate which could be the most reasonable to justify the observed products. The cyclization step could, in principle, generate (Scheme 7) the tetrahydroindole **14** (through a 6-*endo*-*trig* Michael addition) or the dihydroindole **15** (through an overall S_N_V 6-*exo*-*trig* process). A third possible way (S_N_V with ICD [29,30]) to **16** could be labeled again as a 6-*endo*-*trig* process. The main products (**6**–**8**) actually observed can find in **15** an obvious common precursor, while **14** could be the precursor not only of **6**–**8** but also of **9**, observed in our system only in a few cases (**4g**–**i**). As far as **16** is concerned, it could be at most a competitive precursor only of **8**.

**Scheme 8:**
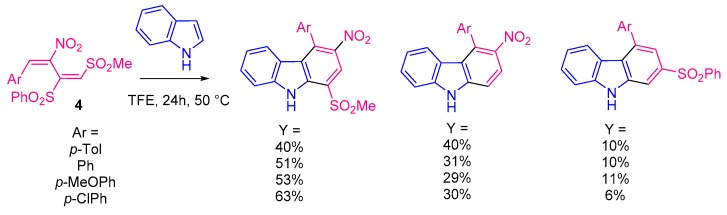
Pertinent results from [16].

As a matter of fact, experimental results in previously studied systems, as well as Baldwin’s rules [31], encouraged us to consider the intermediate **15** as the most likely one, although it is only through **14** that **9** can originate. Thus, the observed distribution could be the result of a competition between these two alternative cyclization ways, rather than the consequence of different evolutions of **14**, with **15** playing a significant role anyway: In this regard, it is worth mentioning what we observed in the reactions with indole, where a trisubstituted derivative analogous to **9** was generally the main product (Scheme 8, results from [16]).

**Scheme 9:**
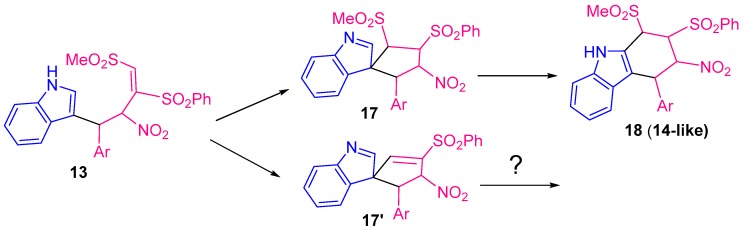
Evolution from **14** to tetrahydrocarbazole **19** through the hypothetic spiro precursor **18**.

Admitting analogous competitions in the pyrrole and indole systems, one should deduce that for indole the double Michael addition pathway should be favoured. This is not unexpected, considering that in analogy with the mechanism hypothesized for the well-known Pictet–Spengler reactions [32,33], formation of the new hexa-atomic ring (**18**) passes probably through a spiro precursor (**17** in Scheme 9), whose stability and evolution could be disadvantaged in the case of an unsaturated system (**17′**) generated by S_N_V with loss of MeSO_2_H.

**Scheme 10:**
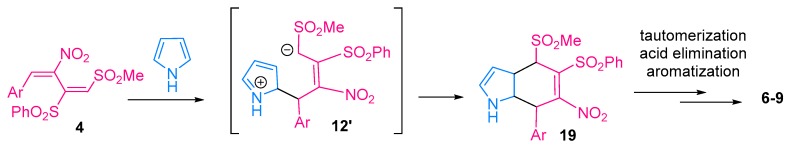
Alternative ionic cycloaddition to tetrahydroindole **19**.

On the other hand, an alternative cyclization mechanism that we considered in the analogous reactions of dinitrobutadienes **3** (Scheme 1, part *b*) also, cannot be, in principle, excluded: The initially formed zwitterionic form of **12** (**12′**) could evolve through an ionic cycloaddition in which the hexa-atomic ring closes with an inversion of polarity, generating **19**, not yet aromatic in the penta-atomic ring (Scheme 10). From this tetrahydroindole, processes involving tautomerizations, elimination(s) of acid and oxidative aromatizations (not necessarily in this sequence) would generate all the observed products.

While in the cited case of dinitrobutadienes **3** that eventuality better fits with the isolation of intermediate **5** (Scheme 1), it should be recalled that no intermediates were ever isolated herein, although a zwitterion would be perhaps more justified than in the previous system. Actually, the presence of the electron-withdrawing –SO_2_Me group on C4 would increase the stabilization of the negative charge delocalized by the nitrovinylic moiety, making intermediate **12′** a better nucleophile and a weaker base. This last hypothesis seems more intriguing, but only the interception of **19**, possibly by trapping with a suitable diene, could furnish an adequate evidence.

## 4. Materials and Methods

^1^H NMR and ^13^C NMR spectra were recorded with a Varian Mercury 300 Plus spectrometer, at 300 and 75 MHz, respectively; chemical shifts (TMS as internal reference) are reported as *δ* values (ppm). Signals are designated as follows: s, singlet; d, doublet; dd, doublet of doublets; ddd, doublet of doublet of doublets; t, triplet; dt, doublet of triplets; tt, triplet of triplets; td, triplet of doublets; m, multiplet; br, broad; app, apparent. High-resolution mass spectra (HRMS) were obtained with an Agilent MSD TOF mass spectrometer, and recorded in positive ion mode with an electrospray (ESI) source. Melting points were determined with a Büchi 535 apparatus and are uncorrected. IR spectra were recorded on a Perkin Elmer Spectrum 65 FT-IR and wave numbers are reported in cm^−1^. Petroleum ether and light petroleum refer to the fractions with bp 40–60 °C and 80–100 °C, respectively. Silica gel 230–400 mesh *Acros Organics* (50–200 μm) were used for column chromatography, all solvents being used as received. TLC analyses were performed on commercially prepared 60 F_254_ silica gel plates and visualized by UV irradiation (eluent: petroleum ether/ethyl acetate). All commercially available reagents were used as received.

### 4.1. Substrates

The employed substrates **4a**–**c**,**h**–**i** [34], **4e** [16] and **4g** [27] were known compounds, prepared according to the reported procedures. Sulfones **4d** and **4f**, and the corresponding precursors, sulfides **4′d** and **4”f**, are new compounds, prepared following the same protocols and obtained with yields from good to satisfactory (60%–90%; details in the Supporting Information).

*1-Chloro-3-[(1E,3E)-4-(methylsulfonyl)-2-nitro-3-(phenylsulfonyl)buta-1,3-dien-1-yl]benzene* (**4d**)

Yellow solid, mp 129–131 °C (taken-up with petroleum ether/DCM). ^1^H NMR (CDCl_3_, 300 MHz): δ (ppm) 3.08 (3H, s), 7.20–7.42 (6H, m), 7.51–7.58 (1H, m), 7.71–7.75 (2H, m), 7.99 (1H, s), 8.27 (1H, s). ^13^C NMR (CDCl_3_, 75 MHz): δ (ppm) 42.57, 128.49, 129.40, 129.56, 130.32, 130.52, 130.88, 132.01, 135.07, 135.36, 135.80, 136.95, 139.64, 142.44, 144.92. HRMS (ESI) *m/z* calculated [M + H]^+^ C_17_H_15_ClNO_6_S_2_^+^ 428.0024, found 428.0102.

*[(1E,3E)-4-(3-chlorophenyl)-3-nitro-2-(phenylsulfonyl)buta-1,3-dien-1-yl](methyl)sulfane* (**4′d**)

Yellow solid, mp 144–146 °C (taken-up with petroleum ether/DCM). ^1^H NMR (CDCl_3_, 300 MHz): δ (ppm) 2.55 (3H, s), 7.19–7.25 (1H, m), 7.30–7.39 (5H, m), 7.43–7.50 (1H, m), 7.71–7.77 (2H, m), 8.18 (1H, s), 8.30 (1H, s). ^13^C NMR (CDCl_3_, 75 MHz): δ (ppm) 17.69, 125.09, 128.10, 128.47, 129.11, 129.97, 130.12, 131.39, 131.69, 133.64, 134.82, 138.98, 139.22, 140.54, 156.45. HRMS (ESI) *m/z* calculated [M + H]^+^ C_17_H_15_ClNO_4_S_2_^+^ 396.0126, found 396.0178.

*1-(Methylsulfonyl)-4-[(1E,3E)-4-(methylsulfonyl)-2-nitro-3-(phenylsulfonyl)buta-1,3-dien-1-yl]benzene* (**4f**)

White solid, mp 183–185 °C (ethanol). ^1^H NMR (acetone-*d*_6_, 300 MHz): δ (ppm) 3.20 (3H, s), 3.35 (3H, s), 7.43–7.51 (2H, m), 7.59–7.66 (1H, m), 7.73 (2H, half AA’BB’, *J* = 8.2 Hz), 7.79–7.87 (4H, m), 8.49 (1H, s), 8.57 (1H, s). ^13^C NMR (acetone-*d*_6_, 75 MHz): δ (ppm) 42.95, 44.01, 128.51, 130.42, 130.75, 132.28, 135.25, 136.44, 137.70, 139.34, 139.89, 143.75, 144.26, 145.98. HRMS (ESI) *m/z* calculated [M + H]^+^ C_18_H_18_NO_8_S_3_^+^ 472.0189, found 472.0205.

*Methyl{4-[(1E,3E)-4-(methylthio)-2-nitro-3-(phenylsulfonyl)buta-1,3-dien-1-yl]phenyl}sulfane* (**4”f**)

Yellow solid, mp 118–119 °C (ethanol). ^1^H NMR (CDCl_3_, 300 MHz): δ (ppm) 2.50 (3H, s), 2.52 (3H, s), 7.10–7.20 (4H, m), 7.37–7.49 (4H, m), 7.52 (1H, d, *J* = 7.5 Hz), 7.74–7.83 (2H, m), 8.25 (1H, s), 8.27 (1H, s). ^13^C NMR (CDCl_3_, 75 MHz): δ (ppm) 14.68, 17.65, 125.43, 125.72, 125.99, 128.24, 129.11, 131.36, 133.62, 137.77, 139.59, 140.88, 145.62, 155.60. HRMS (ESI) *m/z* calculated [M + H]^+^ C_18_H_18_NO_4_S_3_^+^ 408.0392, found 408.0444.

### 4.2. General Procedure for the Synthesis of 7-arylsubstituted indoles (**6**–**8 a**–**h**)

To a stirred suspension of the suitable nitrobutadiene **4a**–**h** (0.245 mmol) in TFE (5 mL, 0.05 M), pyrrole (4 mol equiv) was added. Temperature and time are reported in Table 2. After the time necessary for the disappearance of **4** (5–7 h), the solvent was removed under reduced pressure and the residue purified by flash chromatography over a silica gel column (petroleum ether/ethyl acetate gradients).

*6-Nitro-7-(p-tolyl)-1H-indole* (**6a**)

Waxy solid. IR (ATR): ῦ = 3407 (m, br), 1592 (m), 1508 (s), 1483 (s), 1436 (m), 1413 (m), 1339 (s), 1315 (s), 1255 (m), 1149 (s), 1107 (m), 1092 (m), 1037 (m), 1022 (m) cm^−1^. ^1^H NMR (CDCl_3_, 300 MHz): δ (ppm) 2.43 (3H, s), 6.67 (1H, dd, *J* = 3.2, 2.1 Hz), 7.29 (4H, AA’BB’), 7.37 (1H, dd, *J* = 3.2, 2.6 Hz), 7.66 (1H, d, *J* = 8.7 Hz), 7.85 (1H, d, *J* = 8.7 Hz), 8.28 (1H, br s). ^13^C NMR (CDCl_3_, 75 MHz): δ (ppm) 21.30, 103.55, 116.61, 119.59, 122.02, 128.25, 129.31, 129.84, 130.99, 131.14, 134.57, 138.21, 142.57. HRMS (ESI) *m/z* calculated [M + H]^+^ C_15_H_13_N_2_O_2_^+^ 253.0972, found 253.0993.

*6-Nitro-5-phenylsulfonyl-7-(p-tolyl)-1H-indole* (**7a**)

Yellow solid, mp 141–142 °C (ethanol). IR (ATR): ῦ = 3289 (m, br), 1534 (s), 1446 (m), 1368 (m), 1350 (m), 1306 (s), 1288 (m), 1259 (m), 1141 (s), 1082 (s) cm^−1^. ^1^H NMR (CDCl_3_, 300 MHz): δ (ppm) 2.39 (3H, s), 6.82 (1H, dd, *J* = 3.3, 2.0 Hz), 7.26 (4H, AA’BB’), 7.42 (1H, dd, *J* = 3.2, 2.5 Hz), 7.47–7.62 (3H, m), 7.96–8.02 (2H, m), 8.54 (1H, br s), 8.55 (1H, s). ^13^C NMR (CDCl_3_, 75 MHz): δ (ppm) 21.34, 105.28, 116.15, 120.21, 123.79, 124.96, 127.29, 127.93, 128.68, 128.95, 129.29, 130.14, 133.17, 136.20, 139.75, 141.81, 149.47. HRMS (ESI) *m/z* calculated [M + H]^+^ C_21_H_17_N_2_O_4_S^+^ 393.0909, found 393.0993.

*5-Phenylsulfonyl-7-(p-tolyl)-1H-indole* (**8a**)

Yellow oil. IR (ATR): ῦ = 3293 (m, br), 1708 (m), 1614 (m), 1512 (m), 1446 (m), 1301 (m), 1180 (m), 1149 (s), 1088 (s), 1038 (m), 1019 (m). ^1^H NMR (CDCl_3_, 300 MHz): δ (ppm) 2.35 (3H, s), 6.65 (1H, dd, *J* = 3.3, 2.1 Hz), 7.15–7.30 (4H, m), 7.35–7.49 (4H, m), 7.65 (1H, d, *J* = 1.7 Hz), 7.88-7.92 (2H, m), 8.22 (1H, d, *J* = 1.6 Hz), 8.60 (1H, br s). ^13^C NMR (CDCl_3_, 75 MHz): δ (ppm) 21.25, 104.66, 120.28, 120.81, 126.56, 126.66, 127.35, 127.95, 128.06, 129.08, 130.06, 132.54, 133.16, 134.37, 136.02, 138.28, 142.92. HRMS (ESI) *m/z* calculated [M + H]^+^ C_21_H_18_NO_2_S^+^ 348.1053, found 348.1103.

*7-(4-Phenyl)-6-nitro-1H-indole* (**6b**)

Orange oil. IR (ATR): ῦ = 3285 (m, br), 1598 (w), 1587 (w), 1509 (w), 1490 (s), 1443 (w), 1362 (m), 1343 (m), 1307 (s), 1282 (s), 1252 (s), 1204 (m), 1151 (m), 1102 (s), 1071 (m), 1031 (m) cm^−1^. ^1^H NMR (CDCl_3_, 300 MHz): δ (ppm) 6.68 (1H, dd, *J* = 3.2, 2.1 Hz), 7.35–7.42 (3H, m), 7.43–7.56 (3H, m), 7.67 (1H, d, *J* = 8.7 Hz), 7.87 (1H, d, *J* = 8.7 Hz), 8.30 (1H, br s). ^13^C NMR (CDCl_3_, 75 MHz): δ (ppm) 103.62, 116.64, 119.80, 121.99, 128.38, 128.43, 129.13, 129.41, 131.13, 134.32, 134.51, 142.48. HRMS (ESI) *m/z* calculated [M + H]^+^ C_14_H_11_N_2_O_2_^+^ 239.0815, found 239.0873.

*7-(4-Methoxyphenyl)-6-nitro-1H-indole* (**6c**)

Yellow solid, mp 175–177 °C (taken-up with petroleum ether/DCM). IR (ATR): ῦ = 3356 (m, br), 1610 (m), 1502 (s), 1483 (m), 1412 (w), 1361 (m), 1311 (s), 1257 (m), 1233 (s), 1176 (m), 1148 (m), 1110 (s), 1022 (s) cm^−1^. ^1^H NMR (CDCl_3_, 300 MHz): δ (ppm) 3.87 (3H, s), 6.67 (1H, dd, *J* = 3.2, 2.1 Hz), 7.03 (2H, half AA’BB’), 7.32 (2H, half AA’BB’), 7.37 (1H, dd, *J* = 3.2, 2.6 Hz), 7.65 (1H, d, *J* = 8.7 Hz), 7.82 (1H, d, *J* = 8.7 Hz), 8.32 (1H, br s). ^13^C NMR (CDCl_3_, 75 MHz): δ (ppm) 55.33, 103.62, 114.64, 116.65, 119.57, 121.67, 126.10, 129.23, 129.70, 130.93, 134.77, 142.77, 159.62. HRMS(ESI) *m*/*z* calculated [M + H]^+^ C_15_H_13_N_2_O_3_^+^ 269.0921, found 269.0997.

*7-(3-Chlorophenyl)-6-nitro-1H-indole* (**6d**)

Yellowish solid, mp 122–125 °C (taken-up with petroleum ether/DCM). IR (ATR): ῦ = 3326 (m, br), 1586 (m), 1567 (m), 1510 (m), 1490 (s), 1360 (m), 1350 (m), 1314 (s), 1282 (s), 1251 (s), 1203 (m), 1152 (m), 1104 (s), 1079 (s) cm^−1^. ^1^H NMR (CDCl_3_, 300 MHz): δ (ppm) 6.70 (1H, dd, *J* = 3.2, 2.0 Hz), 7.28 (1H, app td), 7.40–7.50 (4H, m), 7.70 (1H, d, *J* = 8.7 Hz), 7.91 (1H, d, *J* = 8.7 Hz), 8.23 (1H, br s). ^13^C NMR (CDCl_3_, 75 MHz): δ (ppm) 103.85, 116.75, 120.32, 120.51, 126.82, 128.55, 128.63, 129.67, 130.45, 131.42, 134.34, 135.02, 136.23, 142.26. HRMS (ESI) *m*/*z* calculated [M + H]^+^ C_14_H_10_ClN_2_O_2_^+^ 273.0425, found 273.0615.

*7-(4-Chlorophenyl)-6-nitro-1H-indole* (**6e**)

Yellow solid, mp 110–113 °C (taken-up with petroleum ether/DCM). IR (ATR): ῦ = 3306 (m, br), 1598 (w), 1511 (w), 1487 (s), 1360 (m), 1334 (m), 1309 (s), 1281 (m), 1255 (m), 1206 (w), 1150 (m), 1106 (m), 1092 (m), 1016 (m) cm^−1^. ^1^H NMR (CDCl_3_, 300 MHz): δ (ppm) 6.69 (1H, t, *J* = 2.5 Hz), 7.33 (2H, d, *J* = 8.4 Hz), 7.40 (1H, t, *J* = 2.9 Hz), 7.50 (2H, d, *J* = 8.3 Hz), 7.70 (1H, d, *J* = 8.7 Hz), 7.89 (1H, d, *J* = 8.7 Hz), 8.23 (1H, s). ^13^C NMR (CDCl_3_, 75 MHz): δ (ppm) 103.79, 116.77, 120.19, 120.78, 129.44, 129.63, 129.92, 131.34, 132.81, 134.42, 134.55, 142.35. HRMS (ESI) *m*/*z* calculated [M + H]^+^ C_14_H_10_ClN_2_O_2_^+^ 273.0425, found 273.0453.

*7-(4-(Methylsulfonyl)phenyl)-6-nitro-1H-indole* (**6f**)

Yellow solid, mp 248–249 °C (taken-up with petroleum ether/DCM). IR (ATR): ῦ = 3314 (m, br), 1590 (w), 1513 (m), 1494 (m), 1360 (m), 1321 (s), 1293 (s), 1279 (s), 1257 (s), 1209 (m), 1184 (m), 1137 (s), 1109 (s), 1081 (s), 1017 (m) cm^−1^. ^1^H NMR (CDCl_3_, 300 MHz): δ (ppm) 3.17 (3H, s), 6.72 (1H, dd, *J* = 3.1, 2.0 Hz), 7.44 (1H, dd, *J* = 3.2, 2.6 Hz), 7.62 (2H, half AA’BB’, *J* = 8.5 Hz), 7.75 (1H, dd, *J* = 8.7, 0.8 Hz), 7.98 (1H, d, *J* = 8.7 Hz), 8.10 (2H, half AA’BB’, *J* = 8.4 Hz), 8.24 (1H, br s). ^13^C NMR (CDCl_3_, 75 MHz): δ (ppm) 44.53, 104.01, 116.86, 120.13, 120.83, 128.23, 129.75, 130.17, 131.84, 134.14, 140.46, 140.88, 141.96. HRMS (ESI) *m/z* calculated [M + H]^+^ C_15_H_13_N_2_O_4_S^+^ 317.0591, found 317.0703.

*7-(3,5-Bis(trifluoromethyl)phenyl)-6-nitro-1H-indole* (**6g**)

Yellow solid, mp 143–145 °C (taken-up with petroleum ether/DCM). IR (ATR): ῦ = 3353 (m, br), 1513 (m), 1491 (m), 1380 (m), 1361 (m), 1325 (m), 1308 (m), 1276 (s), 1241 (m), 1171 (m), 1167 (m), 1147 (s), 1128 (s), 1108 (s), 1097 (s) cm^−1^. ^1^H NMR (CDCl_3_, 300 MHz): δ (ppm) 6.75 (1H, dd, *J* = 3.3, 2.1 Hz), 7.46 (1H, dd, *J* = 3.3, 2.7 Hz), 7.78 (1H, dd, *J* = 8.8, 0.8 Hz), 7.87 (2H, app s), 7.99-8.05 (2H, m), 8.08 (1H, br s). ^13^C NMR (CDCl_3_, 75 MHz): δ (ppm) 104.28, 117.05, 118.89, 121.24, 122.45 (app quint, *J* = 3.72 Hz), 123.07 (q, *J* = 273.16 Hz), 129.06 (app d, *J* = 3.74 Hz), 130.34, 132.07, 132.58 (q, *J* = 33.30 Hz), 134.28, 137.13, 141.95. HRMS(ESI) *m*/*z* calculated [M + H]^+^ C_16_H_9_F_6_N_2_O_2_^+^ 375.0563, found 375.0635.

*7-(3,5-Bis(trifluoromethyl)phenyl)-2-methylsulfonyl-6-nitro-1H-indole* (**9g**)

Brown solid, mp 222–224 °C (taken-up with petroleum ether/DCM). IR (ATR): ῦ = 3480 (w), 3308 (w, br), 1580 (w), 1520 (m), 1491 (m), 1379 (m), 1348 (m), 1314 (m), 1280 (s), 1245 (m), 1177 (m), 1128 (s), 1109 (s) cm^−1^. ^1^H NMR (acetone-*d_6_*, 300 MHz): δ (ppm) 3.33 (3H, s), 7.19 (1H, dd, *J* = 3.3, 1.8 Hz), 7.97 (1H, t, *J* = 3.0 Hz), 8.25 (1H, app q, *J* = 0.8 Hz), 8.29 (2H, app br s), 8.55 (1H, s), 11.34 (1H, br s). ^13^C NMR (acetone-*d*_6_, 75 MHz): δ (ppm) 43.06, 102.68, 117.18, 122.52 (app. quint., *J* = 3.77 Hz), 123.50 (q, *J* = 273.08 Hz), 124.38, 128.23, 129.66 (app. d, *J* = 3.64 Hz), 131.36, 131.74 (q, *J* = 33.33 Hz), 134.69, 136.50, 136.89, 140.65. HRMS(ESI) *m*/*z* calculated [M + H]^+^ C_17_H_11_F_6_N_2_O_4_S^+^ 453.0265, found 453.0289.

*7-(Naphthalen-1-yl)-6-nitro-1H-indole* (**6h**)

Orange oil. IR (ATR): ῦ = 3410 (m, br), 1589 (m), 1514 (s), 1507 (s), 1492 (s), 1437 (m), 1334 (s), 1309 (s), 1268 (s), 1245 (m), 1208 (m), 1181 (m), 1150 (m), 1129 (m), 1102 (m), 1067 (m), 1036 (m), 1015 (m) cm^−1^. ^1^H NMR (CDCl_3_, 300 MHz): δ (ppm) 6.70 (1H, dd, *J* = 3.2, 2.0 Hz), 7.30 (1H, app. t), 7.34 (2H, app. d), 7.43–7.54 (3H, m), 7.58 (1H, dd, *J* = 8.3, 7.0 Hz), 7.79 (1H, dd, *J* = 8.7, 0.8 Hz), 7.93–7.99 (2H, m), 8.05 (1H, d, *J* = 8.7 Hz). ^13^C NMR (CDCl_3_, 75 MHz): δ (ppm) 103.53, 116.77, 120.20, 120.42, 124.86, 125.65, 126.33, 126.37, 126.82, 128.58, 128.87, 129.55, 131.29, 131.42, 132.01, 133.80, 135.05, 143.25. HRMS (ESI) *m*/*z* calculated [M + H]^+^ C_18_H_13_N_2_O_2_^+^ 289.0972, found 289.0792.

*6-Nitro-7-(thiophen-2-yl)-1H-indole* (**6i**)

Yellow solid, mp 112–114 °C (taken-up with petroleum ether/DCM). IR (ATR): ῦ = 3349 (m, br), 3297 (m, br), 1592 (m), 1504 (s), 1487 (s), 1443 (m), 1355 (m), 1327 (s), 1311 (s), 1279 (s), 1240 (s), 1214 (m), 1191 (m), 1152 (m), 1104 (s), 1085 (s), 1039 (m) cm^−1^. ^1^H NMR (CDCl_3_, 300 MHz): δ (ppm) 6.68 (1H, dd, *J* = 3.2, 2.1 Hz), 7.14–7.23 (2H, m), 7.42 (1H, t, *J* = 2.9 Hz), 7.53 (1H, dd, *J* = 5.0, 1.4 Hz), 7.70 (1H, d, *J* = 8.7 Hz), 7.82 (1H, d, *J* = 8.7 Hz), 8.53 (1H, br s). ^13^C NMR (CDCl_3_, 75 MHz): δ (ppm) 103.77, 114.16, 116.60, 120.70, 127.42, 127.75, 127.92, 129.32, 130.92, 133.46, 135.13, 143.73. HRMS (ESI) *m*/*z* calculated [M + H]^+^ C_12_H_9_N_2_O_2_S^+^ 245.0379, found 245.0409.

### 4.3. General Procedure for the Cadogan Reaction: Synthesis of Pyrrolo[3,2-c]carbazoles 10 

A 5 mL flame-dried, round-bottomed flask, equipped with a condenser, was charged with 0.11 mmol of the relevant **6** and 2.2 mL of triethylphosphite (d = 0.969 g/mL, 2.13 g, 12.8 mmol). The resulting mixture was heated at reflux under nitrogen for 5 h, and then the excess of triethylphosphite was partly removed under reduced pressure (bath temperature 80 °C). The residue was dissolved in ethyl acetate and washed with HCl 5% solution, and water, then dried. The crude was purified by column chromatography (ethyl acetate/petroleum ether 4:1) to isolate compound **10**.

*8-Methyl-1,6-dihydropyrrolo[3,2-c]carbazole* (**10a**)

Grey solid, mp 238–240 °C (taken-up with petroleum ether/DCM). IR (ATR): ῦ = 3426 (m), 3388 (s), 1622 (m), 1590 (w), 1521 (m), 1455 (m), 1418 (m), 1366 (s), 1329 (m), 1282 (m), 1254 (m), 1179 (m), 1075 (m), 1064 (m), 1036 (w) cm^−1^. ^1^H NMR (acetone-*d*_6_, 300 MHz): δ (ppm) 2.51 (3H, s), 6.62 (1H, dd, *J* = 3.1, 2.0 Hz), 7.05 (1H, ddt, *J* = 8.0, 1.4, 0.7 Hz), 7.23–7.30 (2H, m), 7.35 (1H, dt, *J* = 1.5, 0.8 Hz), 7.58 (1H, dd, *J* = 8.5, 0.8 Hz), 8.30 (1H, d, *J* = 8.0 Hz), 10.25 (1H, br s), 10.68 (1H, br s). ^13^C NMR (acetone-*d*_6_, 75 MHz): δ (ppm) 22.12, 103.70, 105.52, 108.46, 111.65, 119.40, 120.60, 121.07, 121.36, 122.29, 122.45, 130.85, 134.29, 138.06, 140.34. HRMS (ESI) *m*/*z* calculated [M + H]^+^ C_15_H_13_N_2_^+^ 221.1073, found 221.1125.

*1,6-Dihydropyrrolo[3,2-c]carbazole* (**10b**) [35]

Grey glassy solid. IR (ATR): ῦ = 3399 (m, br), 1637 (m), 1611 (m), 1424 (m), 1369 (m), 1330 (m) cm^−1^. ^1^H NMR (acetone-*d*_6_, 300 MHz): δ (ppm) 6.63 (1H, dd, *J* = 3.2, 2.0 Hz), 7.21 (1H, ddd, *J* 8.1, 7.2, 1.1 Hz), 7.28–7.32 (2H, m), 7.35 (1H, ddd, *J* = 8.2, 7.2, 1.2 Hz), 7.56 (1H, dt, *J* = 8.1, 1.0 Hz), 7.64 (1H, dd, *J* = 8.5, 0.8 Hz), 8.43 (1H, ddd, *J* = 7.8, 1.3, 0.7 Hz), 10.38 (1H, br s), 10.72 (1H, br s). ^13^C NMR (acetone-*d*_6_, 75 MHz): δ (ppm) 130.78, 105.51, 108.43, 111.54, 119.50, 120.03, 121.63, 122.47, 122.53, 122.75, 124.62, 130.94, 138.15, 139.82. HRMS (ESI) *m*/*z* calculated [M + H]^+^ C_14_H_11_N_2_^+^ 207.0917, found 207.0975.

*8-Methoxy-1,6-dihydropyrrolo[3,2-c]carbazole* (**10c**)

Whitish solid, mp 214–217 °C (taken-up with petroleum ether/DCM). IR (ATR): ῦ = 3444 (w, br), 3390 (m, br), 1617 (s), 1522 (w), 1501 (m), 1459 (m), 1439 (m), 1419 (m), 1368 (s), 1339 (m), 1324 (m), 1304 (m), 1253 (m), 1230 (m), 1220 (m), 1196 (m), 1176 (m), 1161 (m), 1121(m), 1079 (m), 1061 (m), 1041 (m), 1022 (m) cm^−1^. ^1^H NMR (acetone-*d*_6_, 300 MHz): δ (ppm) 3.88 (3H, s), 6.61 (1H, dd, *J* = 3.2, 1.9 Hz), 6.86 (1H, dd, *J* = 8.6, 2.3 Hz), 7.09 (1H, d, *J* = 2.4 Hz), 7.25 (1H, d, *J* = 8.7 Hz), 7.28 (1H, dd, *J* = 3.0, 2.4 Hz), 7.55 (1H, dd, *J* = 8.5, 0.7 Hz), 8.30 (1H, d, *J* = 8.6 Hz), 10.27 (1H, br s), 10.68 (1H, br s). ^13^C NMR (acetone-*d*_6_, 75 MHz): δ (ppm) 55.78, 95.41, 103.63, 105.44, 107.52, 107.78, 116.80, 118.52, 122.21, 122.50, 122.62, 130.58, 137.97, 141.13, 158.86. HRMS (ESI) *m*/*z* calculated [M + H]^+^ C_15_H_13_N_2_O^+^ 237.1022, found 237.1092.

*7-Chloro- (10d) and 9-Chloro-1,6-dihydropyrrolo[3,2-c]carbazole* (**10d’**)

The product obtained (yellow solid) was revealed, upon the ^1^H NMR analysis, to be a mixture of 7-chloro and 9-chloro isomers, in a ratio of about 1:1. Only the 7-chloro derivative (**10d**) was obtained pure: ^1^H NMR (acetone-*d*_6_, 300 MHz): δ (ppm) 6.66 (1H, dd, *J* = 3.1, 2.0 Hz), 7.23 (1H, t, *J* = 7.8 Hz), 7.34 (1H, dd, *J* = 3.2, 2.4 Hz), 7.37–7.44 (2H, m), 7.71 (1H, dd, *J* = 8.6, 0.8 Hz), 8.42 (1H, dt, *J* = 7.8, 0.9 Hz), 10.68 (1H, s), 10.84 (1H, s). The ^1^H NMR spectrum of **10d’** was deduced by difference from that of the mixture: ^1^H NMR (acetone-*d*_6_, 300 MHz): δ (ppm) 6.61–6.69 (1H, m), 7.30 (1H, d, *J* = 8.7 Hz), 7.32–7.35 (2H, m), 7.55 (1H, d, *J* = 8.6 Hz), 7.68 (1H, d, *J* = 8.5 Hz), 8.47 (1H, d, *J* = 2.1 Hz), 10.56 (1H, s), 10.90 (1H, s).

*8-Chloro-1,6-dihydropyrrolo[3,2-c]carbazole* (**10e**)

Yellow solid, mp 177–180 °C (taken-up with petroleum ether/DCM). IR (ATR): ῦ = 3444 (m), 3400 (m, br), 1707 (w), 1638 (m), 1604 (m), 1584 (m), 1522 (m), 1477 (w), 1451 (m), 1427 (m), 1415 (m), 1363 (s), 1326 (m), 1290 (m), 1272 (m), 1249 (s), 1175 (m), 1108 (m), 1064 (m), 1033 (m) cm^−1^. ^1^H NMR (CDCl_3_, 300 MHz): δ (ppm) 6.66 (1H, dt, *J* = 3.8, 2.0 Hz), 7.22 (1H, dt, *J* = 8.4, 2.0 Hz), 7.28–7.37 (2H, m), 7.59 (1H, t, *J* = 1.7 Hz), 7.68 (1H, dd, *J* = 8.5, 1.8 Hz), 8.43 (1H, dd, *J* = 8.4, 1.5 Hz), 10.55 (1H, s), 10.81 (1H, s). ^13^C NMR (CDCl_3_, 75 MHz): δ (ppm) 103.85, 103.94, 105.56, 107.98, 111.37, 119.72, 120.64, 121.52, 122.64, 122.87, 129.82, 130.61, 138.61, 140.37. HRMS (ESI) *m*/*z* calculated [M + H]^+^ C_14_H_10_ClN_2_^+^ 241.0527, found 241.0597.

*1,6-Dihydrobenzo[c]pyrrolo[2,3-g]carbazole* (**10h**)

Grey solid, mp 227–230 °C (taken-up with petroleum ether/DCM). IR (ATR): ῦ = 3480 (m), 3382 (m, br), 1699 (w, br), 1613 (m), 1582 (m), 1530 (m), 1392 (m), 1368 (m), 1349 (s), 1302 (s), 1259 (m) cm^−1^. ^1^H NMR (acetone-*d*_6_, 300 MHz): δ (ppm) 6.74 (1H, dd, *J* = 3.2, 1.8 Hz), 7.37 (1H, dd, *J* = 3.2, 2.6 Hz), 7.43–7.50 (2H, m), 7.65–7.72 (2H, m), 7.84 (2H, AB, *J* 17.6, 8.8 Hz), 8.05 (1H, dm, *J* = 8.0 Hz), 9.06 (1H, ddt, *J* = 8.4, 1.3, 0.7 Hz), 10.60 (1H, br s), 10.99 (1H, br s), ^13^C NMR (acetone-*d*_6_, 75 MHz): δ (ppm) 104.61, 106.37, 109.87, 114.24, 116.12, 119.57, 122.56, 123.33, 123.67, 124.88, 126.52, 126.97, 130.11, 130.19, 130.30, 130.38, 137.09, 137.53. HRMS (ESI) *m*/*z* calculated [M + H]^+^ C_18_H_13_N_2_^+^ 257.1073, found 257.1097.

*1,6-Dihydropyrrolo[2,3-e]thieno[3,2-b]indole* (**10i**)

Whitish solid, mp 181–182 °C (taken-up with petroleum ether/DCM). IR (ATR): ῦ = 3389 (s, br), 1710 (w), 1628 (w), 1517 (w), 1432 (m), 1389 (s), 1372 (s), 1318 (m), 1294 (m), 1263 (m), 1249 (m), 1234 (m), 1182 (m), 1097 (m), 1075 (m), 1062 (m), 1042 (m), 1030 (m) cm^−1^. ^1^H NMR (CDCl_3_, 300 MHz): δ (ppm) 6.59 (1H, dd, *J* = 3.1, 2.0 Hz), 7.21 (1H, d, *J* = 5.2 Hz), 7.24–7.30 (2H, m), 7.42–7.48 (2H, m), 10.51 (1H, br s), 10.68 (1H, br s). ^13^C NMR (CDCl_3_, 300 MHz): δ (ppm) 103.77, 106.83, 108.46, 112.68, 115.34, 117.05, 122.00, 122.27, 126.26, 128.71, 139.53, 142.98. HRMS (ESI) *m*/*z* calculated [M + H]^+^ C_12_H_9_N_2_S^+^ 213.0481, found 213.0499.

### 4.4. Procedure for the Reduction of the Nitro- to Amino- Group in Compound **6a**

To a solution of **6a** (85 mg, 0.34 mmol) in dry ethanol (3.5 mL), SnCl_2_·2H_2_O (768 mg, 10.0 mmol) was added. The reaction mixture was left overnight at 60 °C under an argon atmosphere. After removal of the solvent under reduced pressure, the crude residue was partitioned between ethyl acetate and KOH 2M. The aqueous layer was extracted with ethyl acetate (3 × 10 mL), and the combined organic extracts washed with brine (2 × 10 mL) and water (3 × 10 mL), then dried (Na_2_SO_4_), and concentrated under reduced pressure. A flash silica-gel column chromatography (petroleum ether/DCM 1:1) of the crude residue gave compound **11a** (46 mg, 61%).

*7-(p-Tolyl)-1H-indol-6-amine* (**11a**)

Whitish solid, mp 127–129 °C (taken-up with petroleum ether/DCM). IR (ATR): ῦ = 3368 (m, br), 3313 (w, br) 1618 (s), 1503 (m), 1432 (s), 1335 (m), 1248 (m), 1185 (w), 1106 (m), 1060 (w), 1022 (w) cm^−1^. ^1^H NMR (CDCl_3_, 300 MHz): δ (ppm) 2.45 (3H, s), 3.25 (2H, br), 6.47 (1H, app t, *J* = 2.5 Hz), 6.69 (1H, d, *J* = 8.3 Hz), 6.94 (1H, app t, *J* = 2.7 Hz), 7.32–7.45 (5H, m), 7.82 (1H, br). ^13^C NMR (CDCl_3_, 300 MHz): δ (ppm) 21.26, 102.70, 110.22, 111.17, 120.34, 120.98, 122.10, 129.74, 130.23, 132.34, 135.94, 137.39, 138.36. HRMS(ESI) *m*/*z* calculated [M + H]^+^ C_15_H_15_N_2_^+^ 223.1230, found 223.1322.

## 5. Conclusions

In conclusion, in the present research we have explored a pyrrole-to-indole benzannulation procedure making use of non-symmetric nitrobutadienic C_4_ building blocks, that allowed us to obtain, in satisfactory yields and good selectivity, unusual nitroindolic derivatives still unknown in the literature, and further amenable to interesting elaborations. Thanks to the easy reducibility of the nitro group and to the relative position of substituents, novel and interesting pyrrolo[3,2-*c*]carbazoles could be obtained applying a reported protocol for the Cadogan reaction (non-optimized for our compounds), while the promising easy reduction of nitro to amino derivatives opens the way to further elaborations of **6** to other polyheterocyclic systems; for instance, pyrrolo[2,3-*a*]phenanthridines, by means of the Pictet–Spengler protocol [32,33].

## Figures and Tables

**Table 1:** Search for conditions of choice in the reactions of nitrobutadiene **4a** with pyrrole ^1^.

Entry	T (°C)	Mol Eq. of Pyrrole	time (h)	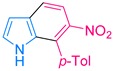 6a (Yield %)	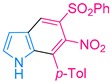 7a (Yield %)	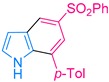 8a (Yield %)	Overall Balance
1	50	2	24	52	16	14	82%
2	50	4	2.5	53	21	17	91%
3	40	4	5	57	17	12	86%
4	30	4	24	57	16	19	92%
5	0	4	48	-	-	-	100% of **4a**

^1^ Yields reported are for chromatographically isolated compounds.

**Table 2:** Results obtained in the reactions of nitrobutadienes **4a**–**i** with pyrrole in TFE at 40 °C for 5 h ^1^.

Entry	Ar in 4	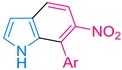 6 (Yields %)	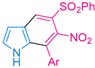 7 (Yields %)	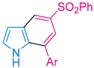 8 (Yields %)	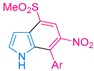 9 (Yields %)	Overall Balance
1	*p*-Tol: **4a**	**6a**: 57	**7a**: 17	**8a**: 12	**9a**: nq^2^	86%
2	Ph: **4b**	**6b**: 60	**7b**: 10	**8b**: 10	**9b**: -^3^	80%
3	*p*-MeOC_6_H_4_: **4c**	**6c**: 44	**7c**: 17	**8c**: 13	**9c**: -^3^	74%
4	*m*-ClC_6_H_4_: **4d**	**6d**: 53	**7d**: 11	**8d**: 18	**9d**: -^3^	81%
5	*p*-ClC_6_H_4_: **4e**	**6e**: 42	**7e**: 8	**8e**: 15	**9e**: 18	83%
6	*p*-MeSO_2_C_6_H_4_: **4f**	**6f**: 19	**7f**: nq^2^	**8f**: nq^2^	**9f**: nq^2^	nq
7	3,5-(CF_3_)_2_C_6_H_3_: **4g**	**6g**: 22	**7g**: 10	**8g**: 11	**9g**: 25	68%
8	1-Naphthyl: **4h**	**6h**: 40	**7h**: 14	**8h**: 13	**9h**: 13	80%
9	2-Thienyl:^4^ **4i**	**6i**: 33	**7i**: 7	**8i**: 11	**9i**: 14	65%

^1^ Yields reported are for chromatographically isolated compounds, unless otherwise specified. ^2^ Observed by TLC and hypothesized on the grounds of ^1^H NMR signals, but not quantified. ^3^ Not evidenced by TLC. ^4^ Reaction time: 7h.

**Table 3:** Results obtained in the Cadogan reaction (triethyl phosphite at reflux, 5 h, under Ar) applied to 7-aryl-6-nitroindoles **6** ^1^.

Entry	Substrate		Product	Yield
1	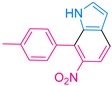	**6a**	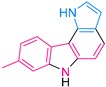	**10a**: 75%
2	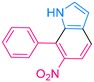	**6b**	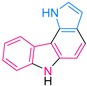	**10b**: 50%
3	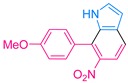	**6c**	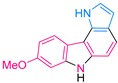	**10c**: 53%
4	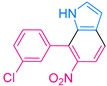	**6d**	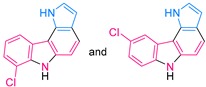	**10d** and **10d’**: 75% ca. 1:1
5	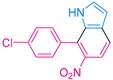	**6e**	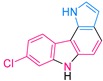	**10e**: 51%
6	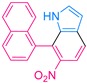	**6h**	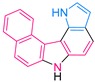	**10h**: 65%
7	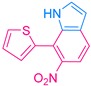	**6i**	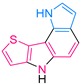	**10i**: 38%

^1^ Yields reported are for chromatographically isolated compounds.

## Supplementary Materials

The following are available online: Detailed description on preparative procedures of substrates **4d**, **4f**, and the corresponding precursors, **4′d**, **4”f**: ^1^H NMR and ^13^C NMR spectra for all new compounds.
