# Supplementary material for: Sequential Annulations to Interesting Novel Pyrrolo[3,2-c]carbazoles
Source: Molecules. 2019 Oct 22;24(20):3802. doi: 10.3390/molecules24203802 (PMC6832463; doi:10.3390/molecules24203802)
Supplement: Supplementary file 1 [file molecules-24-03802-s001.pdf]

Supporting Information for the Article:

Sequential annulations to interesting novel pyrrolo[3,2-*c*]carbazoles

Alice Benzi, Lara Bianchi, Massimo Maccagno, Angela Pagano, Giovanni Petrillo, and Cinzia Tavani*

Department of Chemistry and Industrial Chemistry, Università di Genova, Via Dodecaneso 31, I-16146 Genova, Italy; http://www.chimica.unige.it

* Correspondence: cinzia.tavani@unige.it; Tel.: +39-010-3536112

Index:

1. Detailed procedures for the synthesis of substrates **4d**, **4f**, **4′d**, **4″f** S1-S3
2. ^{1}H and ^{13}C NMR for all new compounds S4-S27

1. *Detailed procedures for the synthesis of substrates* **4d**, **4f**, **4′d**, **4″f**

The optimized general procedure for preparation of sulfones **4** has already been reported.[1] In this context, it was applied for the first time to prepare compounds **4d** and **4f**. The overall synthethic protocol is represented in **Scheme 1**. The starting point was compound **B** obtained from 2-phenylsulfonyl-3-nitrothiophene **A**, applying a ring-opening reaction with pyrrolidine and silver nitrate, followed by the one-pot methylation of the generated silver enthiolate, and the substitution of the pyrrolidino group with the desired Ar by reaction with ArMgX.[2]

For this work, from **B**, treatment with *m*-chlorophenyl magnesium bromide generates sulfide **4′d**, while treatment with *p*-methylthiophenyl magnesium bromide generates sulfide **4″f.** The last step to obtain **4d** requires oxidation of –SMe to –SO_2Me, traditionally performed with a peroxyacid (*m*-CPBA in DCM, or CH_3COOH/H_2O_2). The same oxidative procedures were applied to **4″f**, whereby both -SMe groups could be oxidized at the same time to produce **4f**.

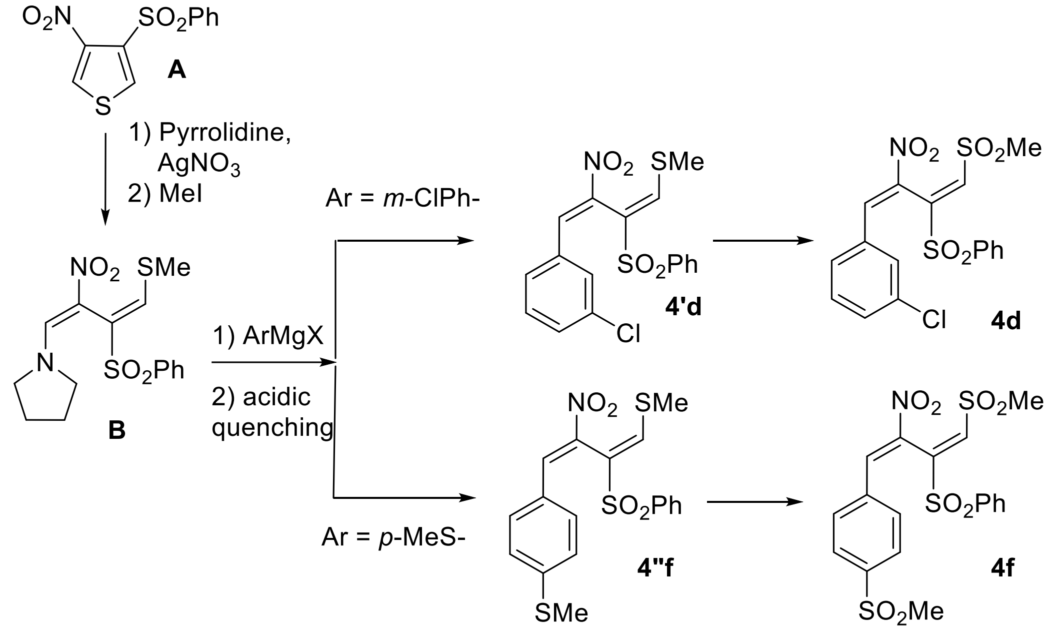

Scheme 1. Synthetic protocol to obtain the desired sulfones **4d** and **4f**.

[1]Bianchi, L.; Dell’Erba, C.; Maccagno, M.; Mugnoli, A.; Novi, M.; Petrillo, G.; Sancassan, F.; Tavani, C. *J. Org. Chem.* **2003**, *68*, 5254-5260.
[2]Dell’Erba, C.; Gabellini, A.; Novi, M.; Petrillo, G.; Tavani, C.; Cosimelli, B.; Spinelli, D. *Tetrahedron* **2001**, *57*, 8159-8165.

[(1*E*,3*E*)-4-(3-chlorophenyl)-3-nitro-2-(phenylsulfonyl)buta-1,3-dien-1-yl](methyl)sulfane (4′d) and
Methyl{4-[(1E,3E)-4-(methylthio)-2-nitro-3-(phenylsulfonyl)buta-1,3-dien-1-yl]phenyl}-sulfane (4″f)

To a suspension of 1-[(1*E*,3*E*)-4-(methylthio)-2-nitro-3-(phenylsulfonyl)buta-1,3-dien-1-yl]pyrrolidine (MW 354.44, 0.354 g, 1 mmol) in THF (12 mL), cooled to 0 °C, the Grignard reagent solution in THF (1.1 mmol) was slowly added by syringe under argon and magnetic stirring. The reaction mixture was left to reach room temperature, kept under stirring for 15 min (the end of the reaction being judged by TLC analysis), and eventually poured into a dichloromethane-ice-HCl (1.1 mmol) mixture. After separation of the two layers, the aqueous phase was extracted with dichloromethane; the collected organic extracts were washed with water and dried over Na_2SO_4. Concentration under vacuum of the extracts gave a crude that was purified by column chromatography over silica gel (petroleum ether/dichloromethane gradients as eluent). For compound **4′d**, 0.222 g were obtained (MW = 395.87, yield = 56%); for compound **4″f**, 0.236 g were obtained (MW = 407.52, yield = 58%).

1-Chloro-3-[(1*E*,3*E*)-4-(methylsulfonyl)-2-nitro-3-(phenylsulfonyl)buta-1,3-dien-1-yl] benzene (4d) and
1-(Methylsulfonyl)-4-[(1*E*,3*E*)-4-(methylsulfonyl)-2-nitro-3-(phenylsulfonyl)buta-1,3-dien-1-yl]benzene (4f)

To a gently warmed (50 °C) solution of the suitable methyl sulfide (1 mmol) in acetic acid (15 mL), a 50% w/w aqueous solution of hydrogen peroxide (20-30 mol eq.) was added, after cooling at room temperature and under magnetic stirring. Completed the addition, the solution was again warmed at 50 °C and kept overnight. The end of reaction was verified by TLC. After dilution in brine, the solid precipitated was filtered and washed with water. The crude obtained was usually pure by TLC and ^{1}H NMR analysis. For compound **4d**, 0.158 g were obtained (MW = 427.87, yield = 74%); for compound **4f**, 0.215 g were obtained (MW = 471.52, yield = 91%).

4d

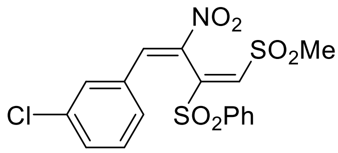
NO2
SO2Me
Cl
SO2Ph

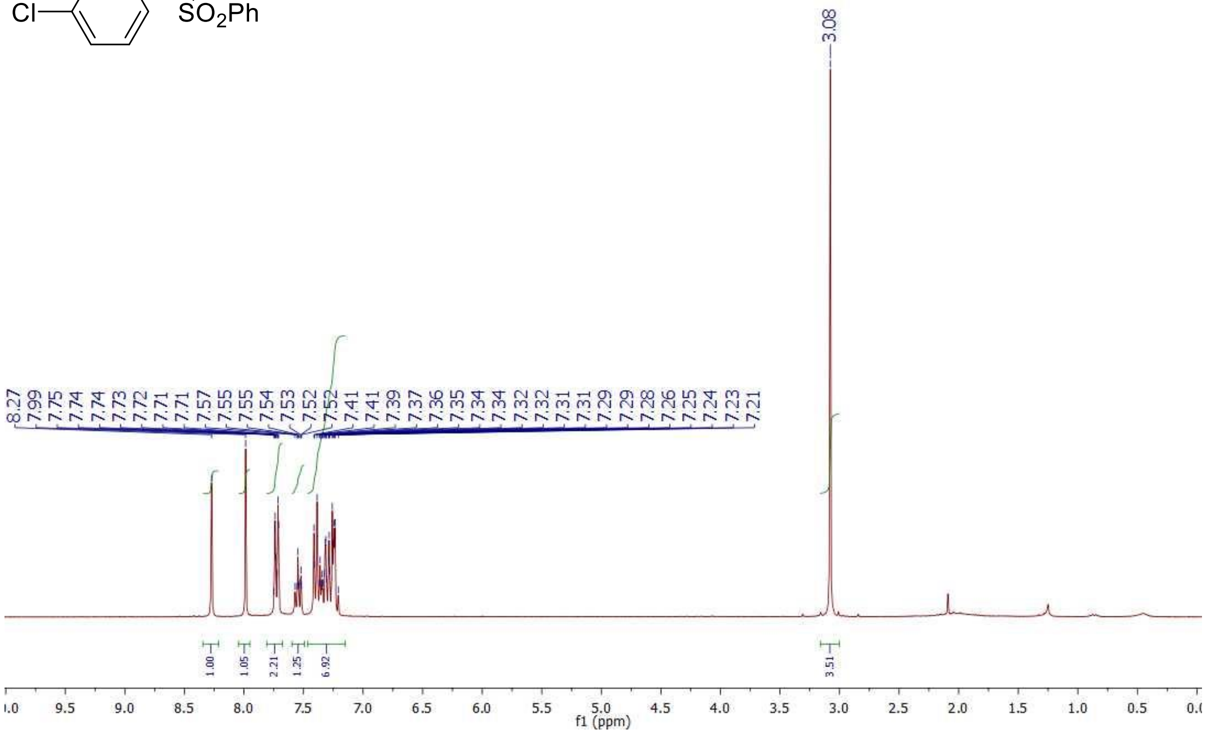
8.27
7.99
7.75
7.74
7.74
7.73
7.72
7.71
7.71
7.57
7.55
7.55
7.54
7.53
7.52
7.52
7.41
7.41
7.39
7.37
7.36
7.35
7.34
7.34
7.32
7.32
7.31
7.31
7.29
7.29
7.28
7.26
7.25
7.24
7.23
7.21
3.08
1.00
1.05
2.21
1.25
6.92
3.51
f1 (ppm)

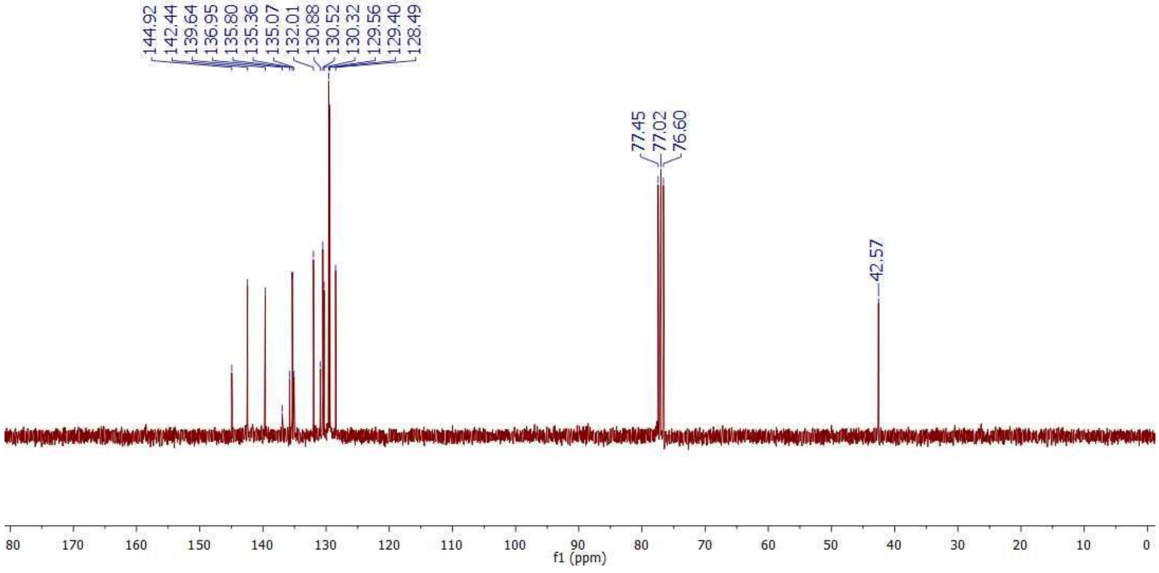
144.92
142.44
139.64
136.95
135.80
135.36
135.07
132.01
130.88
130.52
130.32
129.56
129.40
128.49
77.45
77.02
76.60
42.57
f1 (ppm)

4’d

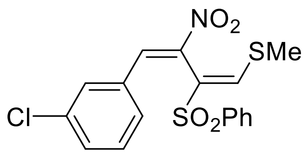

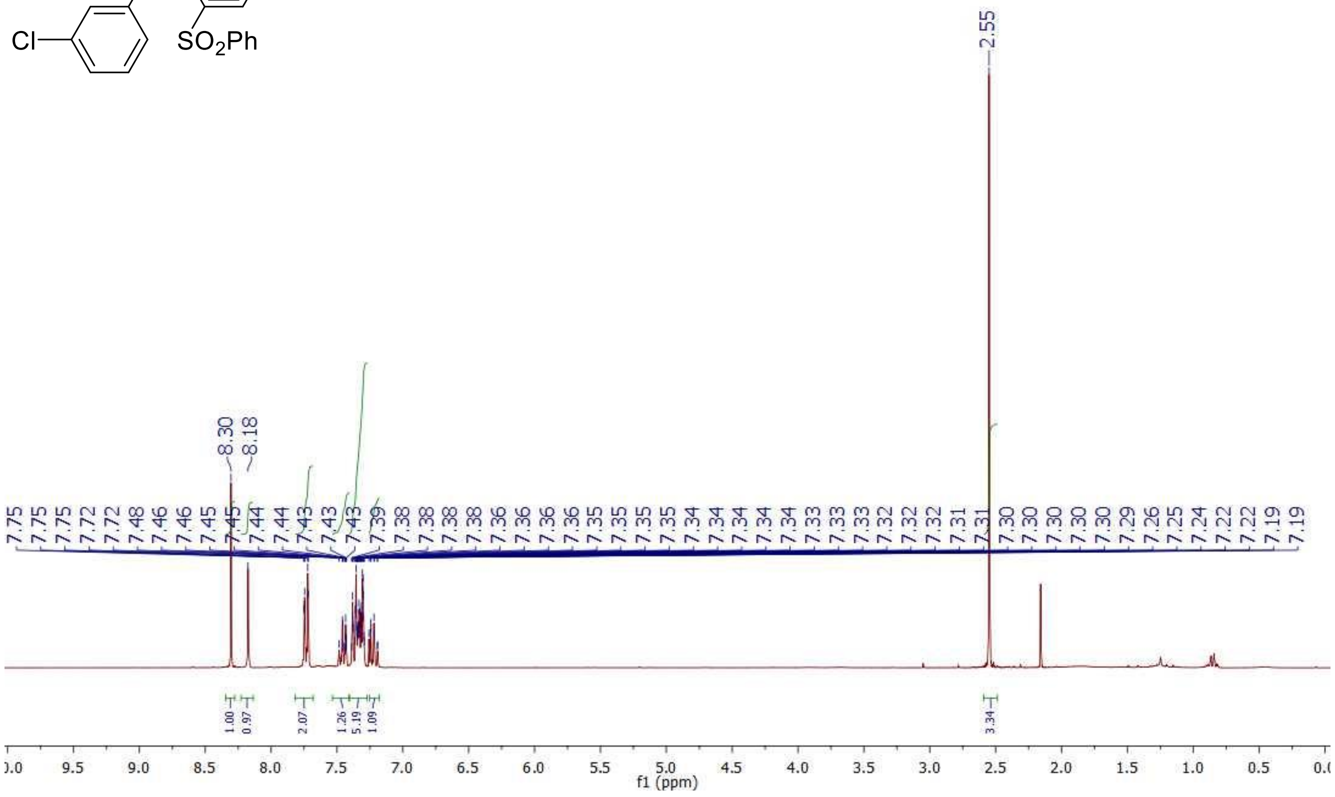

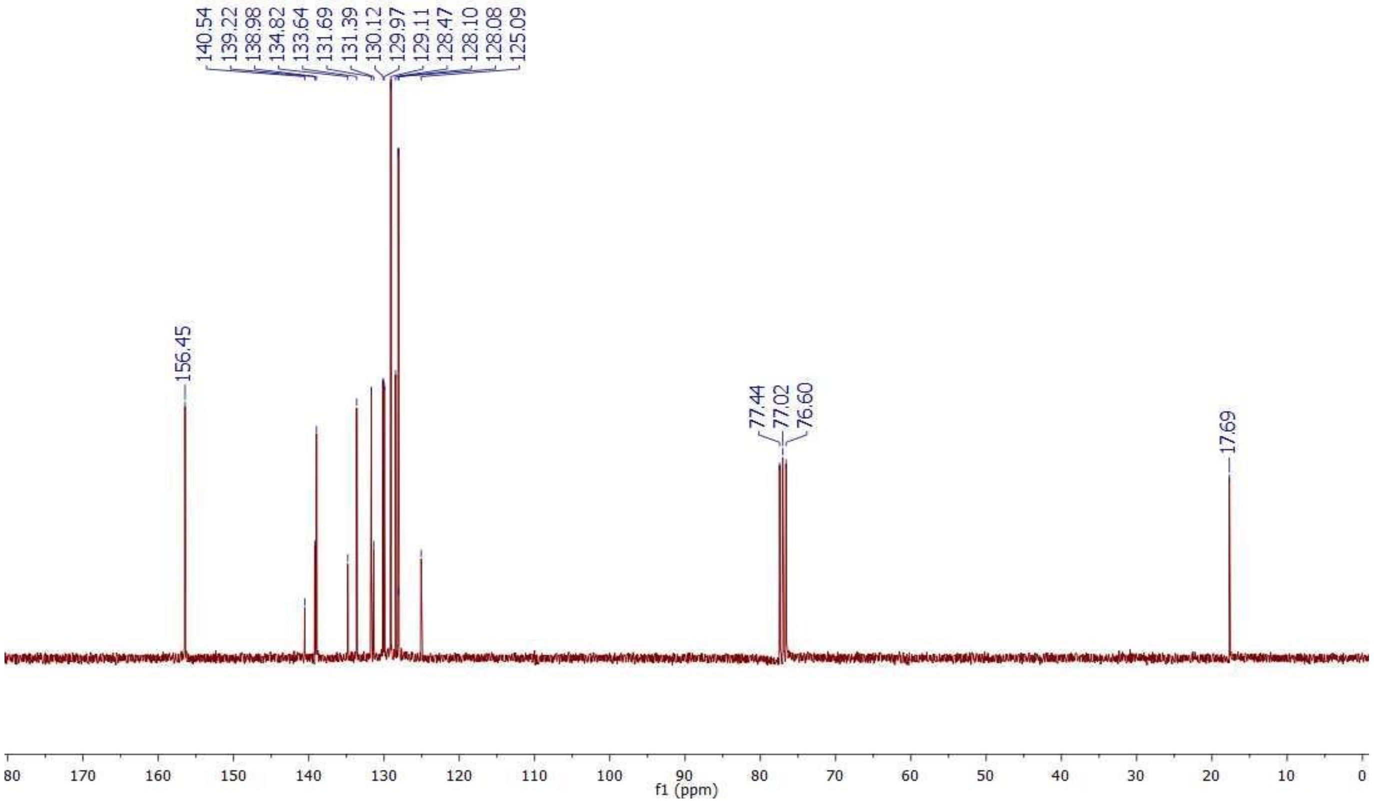

4f

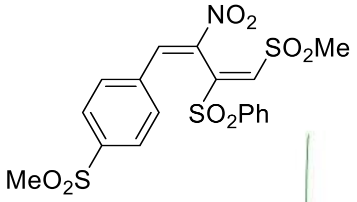

NO2
SO2Me
SO2Ph
MeO2S

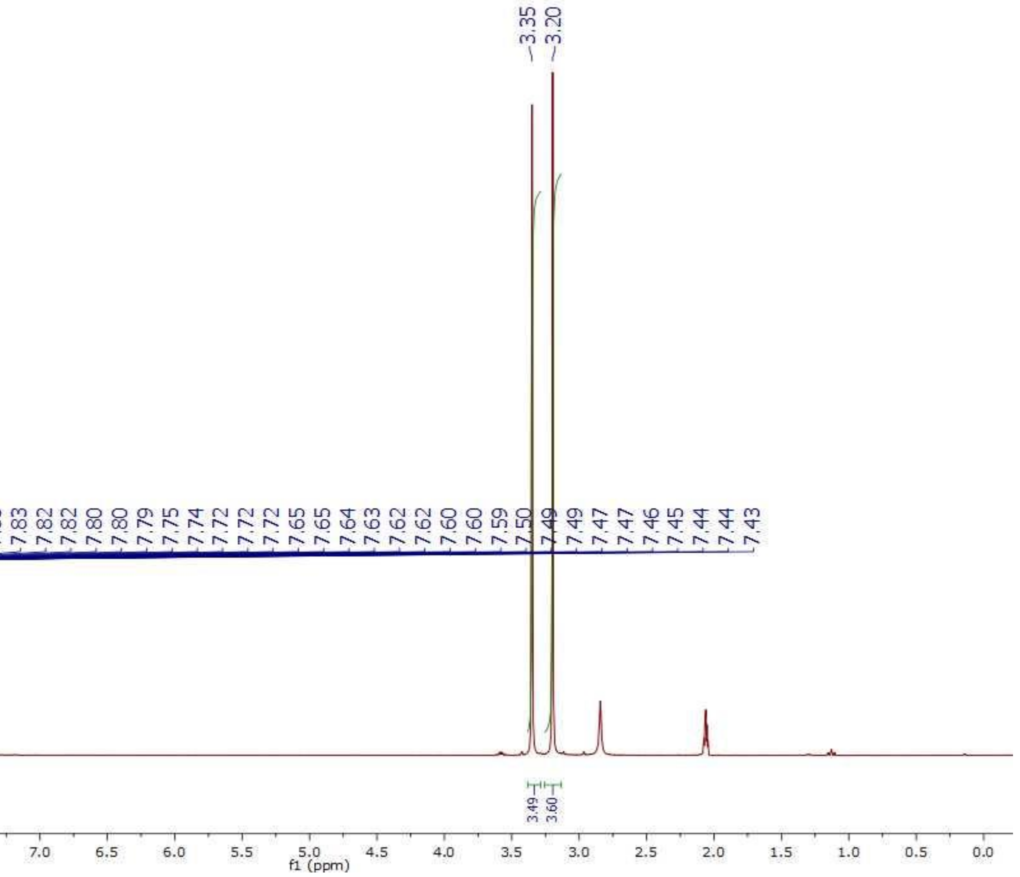

8.57
8.49
7.87
7.86
7.85
7.84
7.83
7.83
7.82
7.82
7.80
7.80
7.79
7.75
7.74
7.72
7.72
7.72
7.65
7.64
7.63
7.62
7.62
7.60
7.60
7.59
7.50
7.49
7.49
7.47
7.47
7.46
7.45
7.44
7.44
7.43
3.35
3.20
1.00
0.91
4.08
2.25
1.09
2.18
3.49
3.60
f1 (ppm)

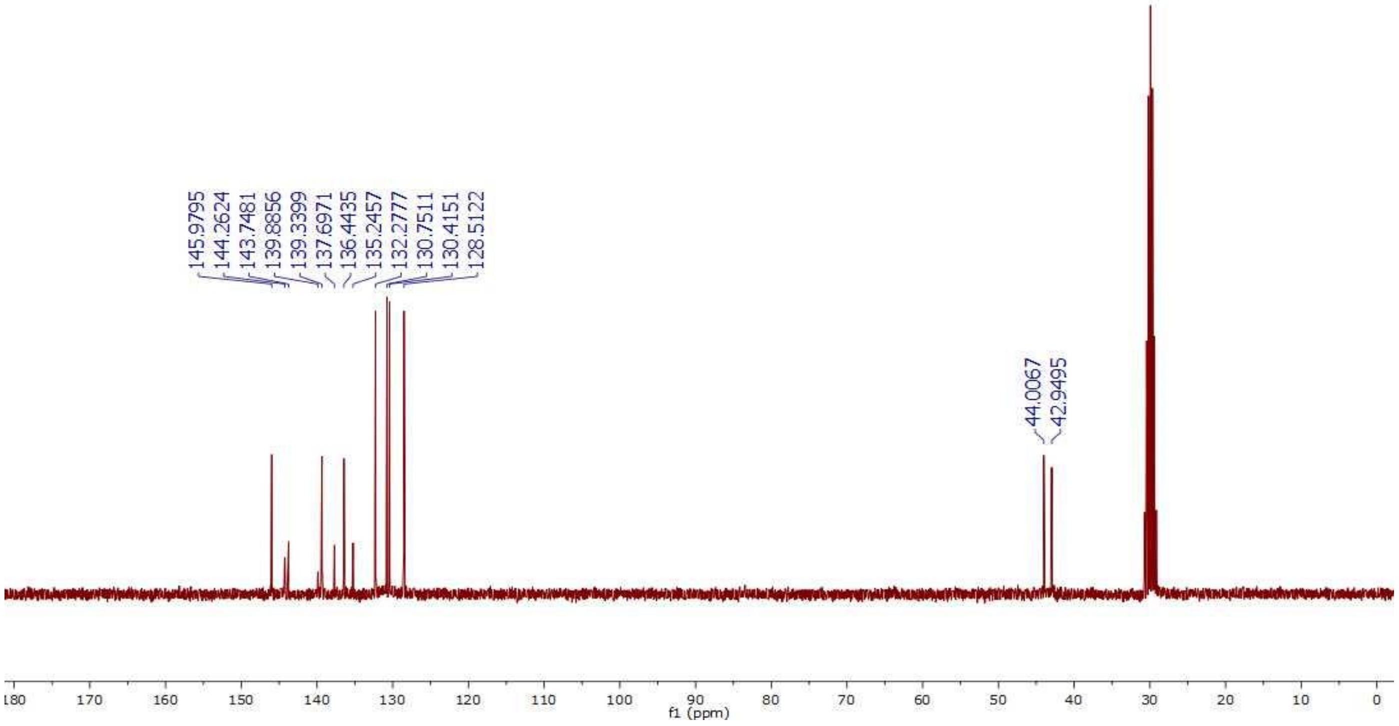

145.9795
144.2624
143.7481
139.8856
139.3399
137.6971
136.4435
135.2457
132.2777
130.7511
130.4151
128.5122
44.0067
42.9495
f1 (ppm)

4”f

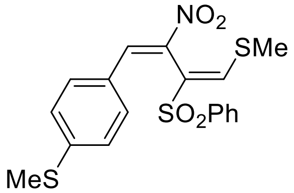
NO2
SMe
SO2Ph
MeS

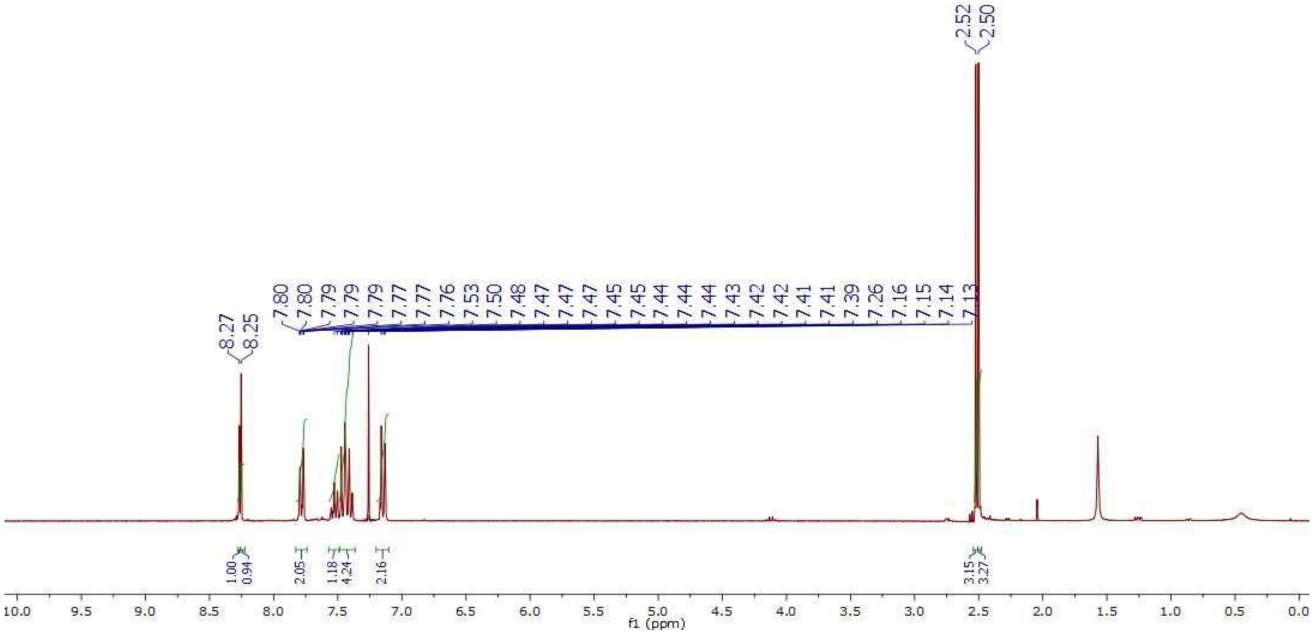
8.27
8.25
7.80
7.80
7.79
7.79
7.79
7.77
7.77
7.76
7.53
7.50
7.48
7.47
7.47
7.47
7.45
7.45
7.44
7.44
7.44
7.43
7.42
7.42
7.41
7.41
7.39
7.26
7.16
7.15
7.14
7.13
2.52
2.50
1.00
0.94
2.05
1.18
4.24
2.16
3.15
3.27
10.0
9.5
9.0
8.5
8.0
7.5
7.0
6.5
6.0
5.5
5.0
4.5
4.0
3.5
3.0
2.5
2.0
1.5
1.0
0.5
0.0
f1 (ppm)

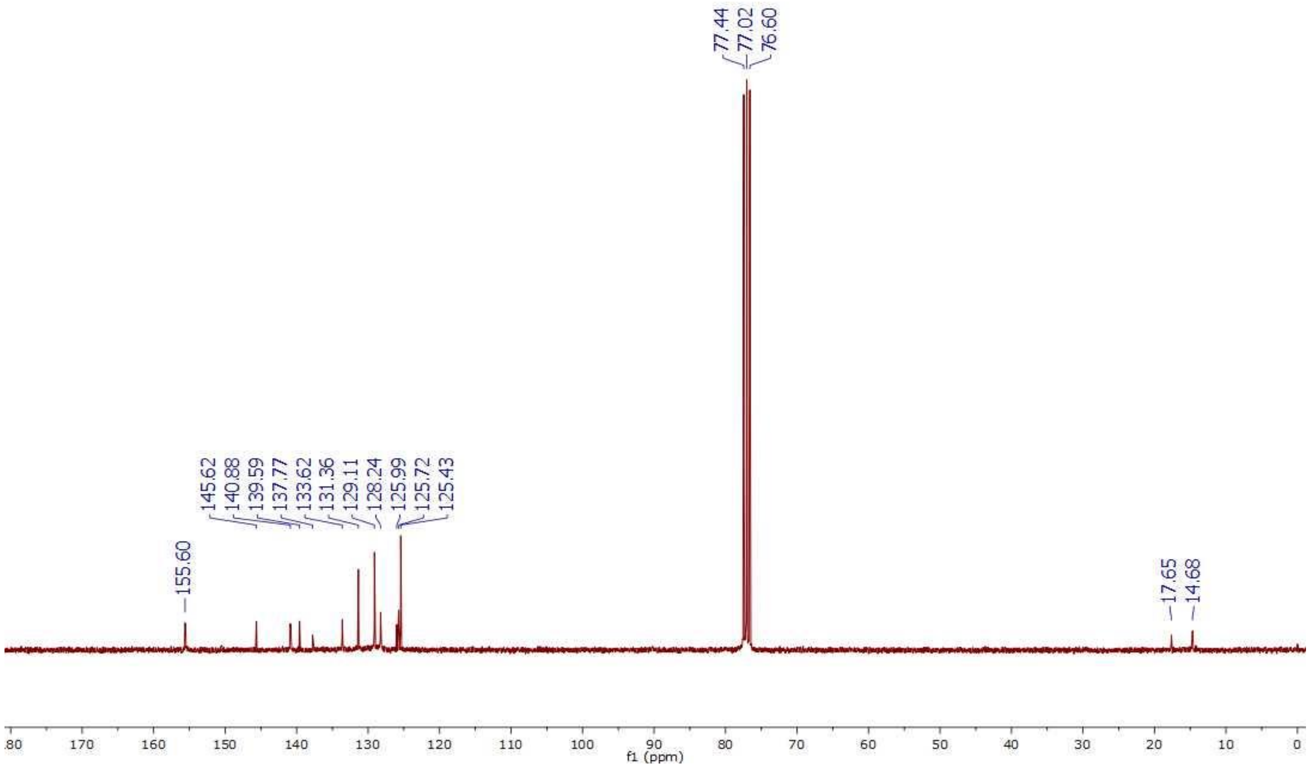
77.44
77.02
76.60
155.60
145.62
140.88
139.59
137.77
133.62
131.36
129.11
128.24
125.99
125.72
125.43
17.65
14.68
80
170
160
150
140
130
120
110
100
90
80
70
60
50
40
30
20
10
0
f1 (ppm)

6a

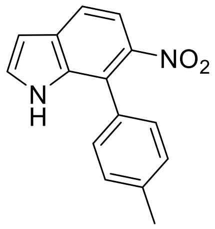
NO2
N
H

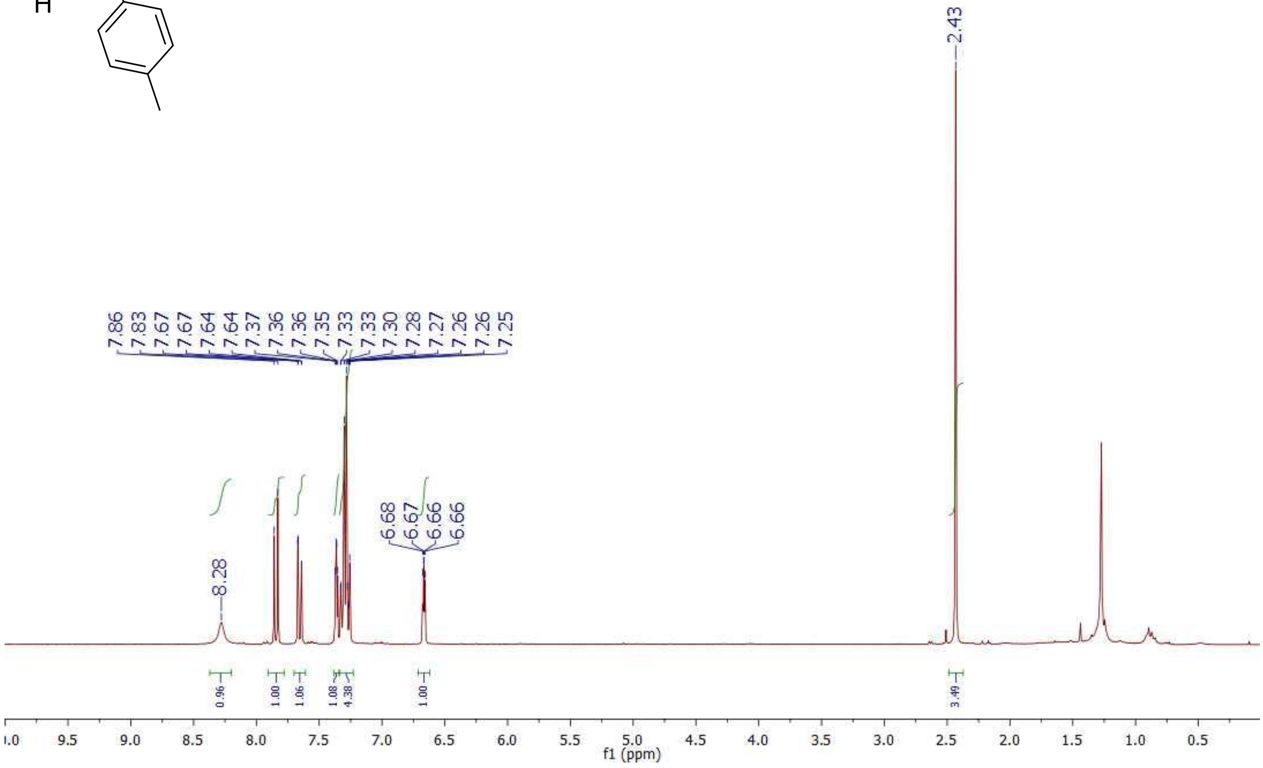
2.43
7.86
7.83
7.67
7.67
7.64
7.64
7.37
7.36
7.36
7.35
7.33
7.33
7.30
7.28
7.27
7.26
7.26
7.25
6.68
6.67
6.66
6.66
8.28
0.96
1.00
1.06
1.08
4.38
1.00
3.49
f1 (ppm)

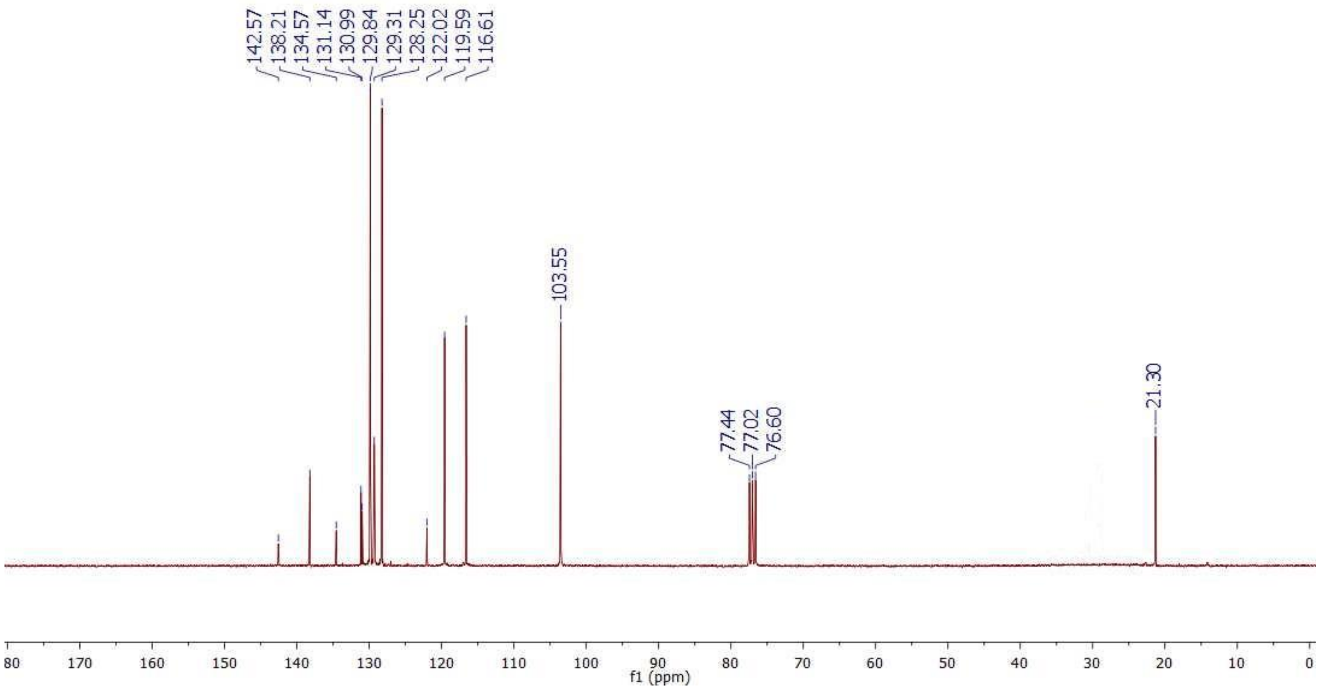
142.57
138.21
134.57
131.14
130.99
129.84
129.31
128.25
122.02
119.59
116.61
103.55
77.44
77.02
76.60
21.30
f1 (ppm)

7a

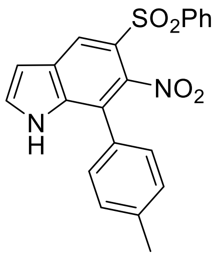
SO2Ph
NO2
N
H

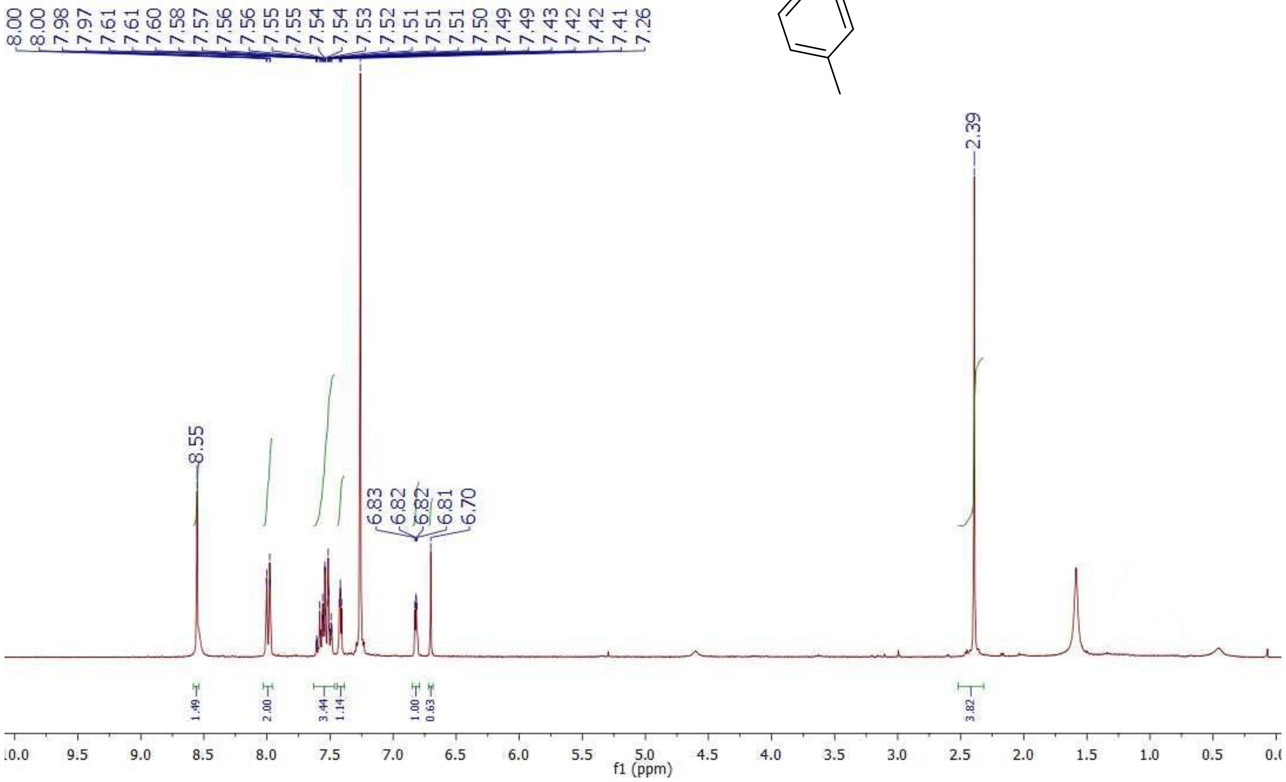
8.00 8.00 7.98 7.97 7.61 7.61 7.60 7.58 7.57 7.56 7.56 7.55 7.55 7.54 7.54 7.53 7.52 7.51 7.51 7.51 7.50 7.49 7.49 7.43 7.42 7.42 7.41 7.26
2.39
8.55
6.83 6.82 6.82 6.81 6.70
1.49 2.00 3.44 1.14 1.00 0.63 3.82
f1 (ppm)

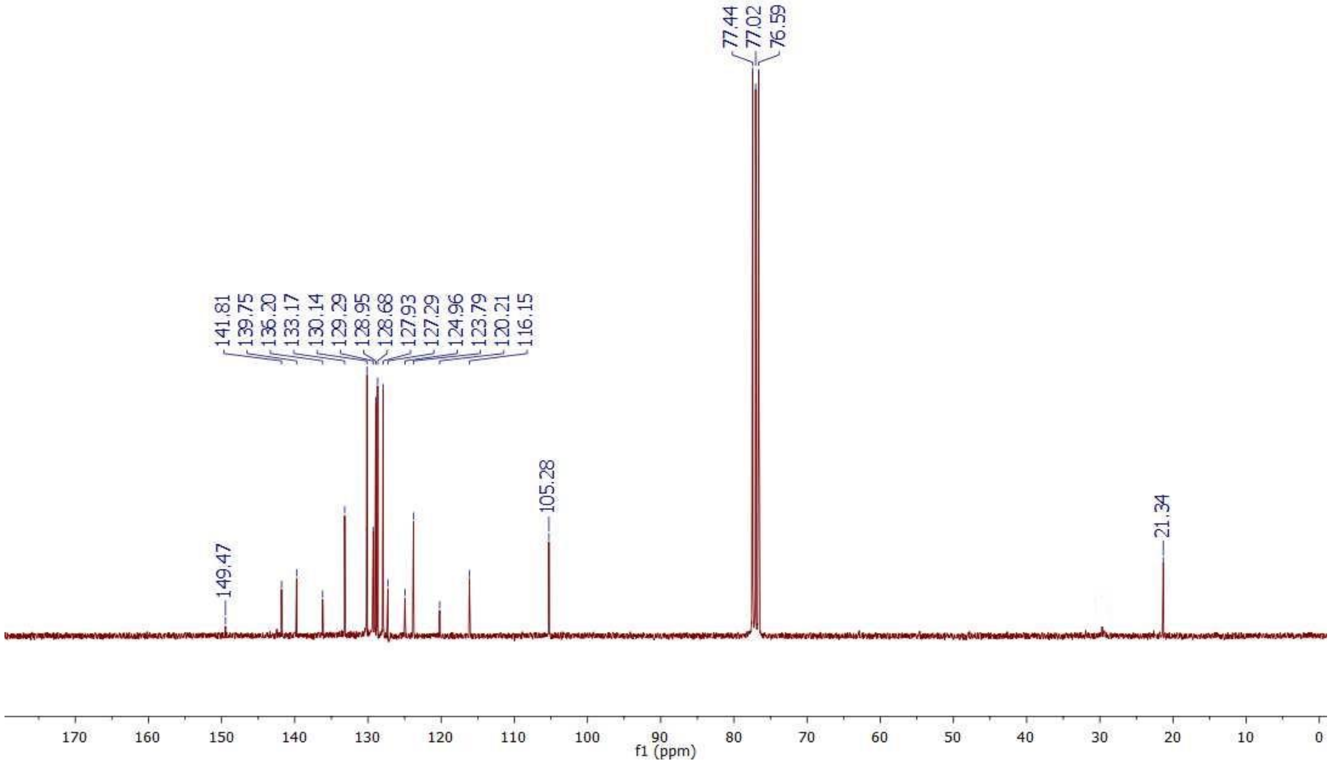
77.44 77.02 76.59
141.81 139.75 136.20 133.17 130.14 129.29 128.95 128.68 127.93 127.29 124.96 123.79 120.21 116.15
105.28
149.47
21.34
f1 (ppm)

8a

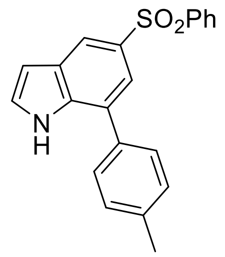
SO$_2$Ph
N
H

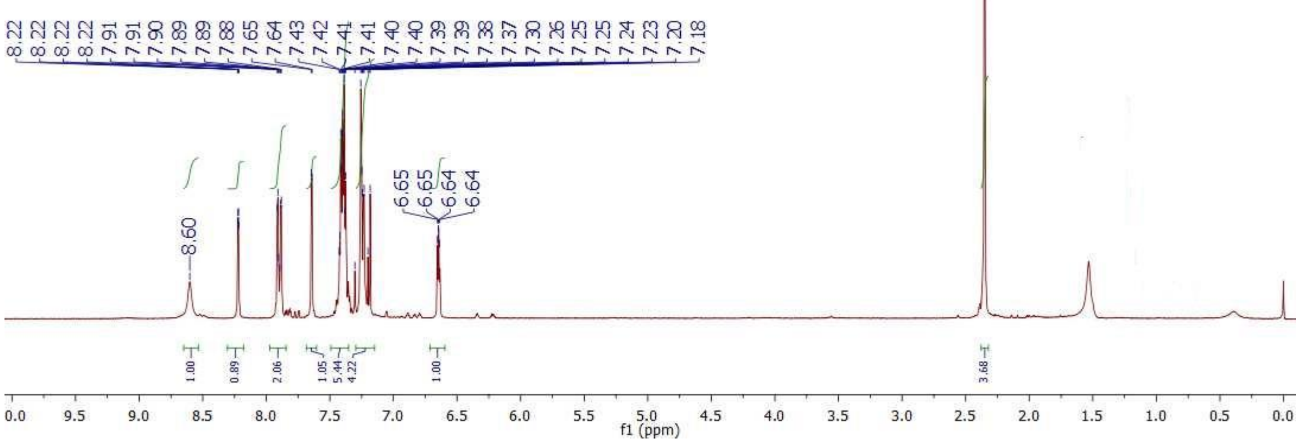
8.22
8.22
8.22
8.22
7.91
7.91
7.90
7.89
7.88
7.65
7.64
7.43
7.42
7.41
7.41
7.40
7.40
7.39
7.39
7.38
7.37
7.30
7.26
7.25
7.25
7.24
7.23
7.20
7.18
2.35
8.60
6.65
6.65
6.64
6.64
1.00
0.89
2.06
1.05
5.44
4.22
1.00
3.68
f1 (ppm)

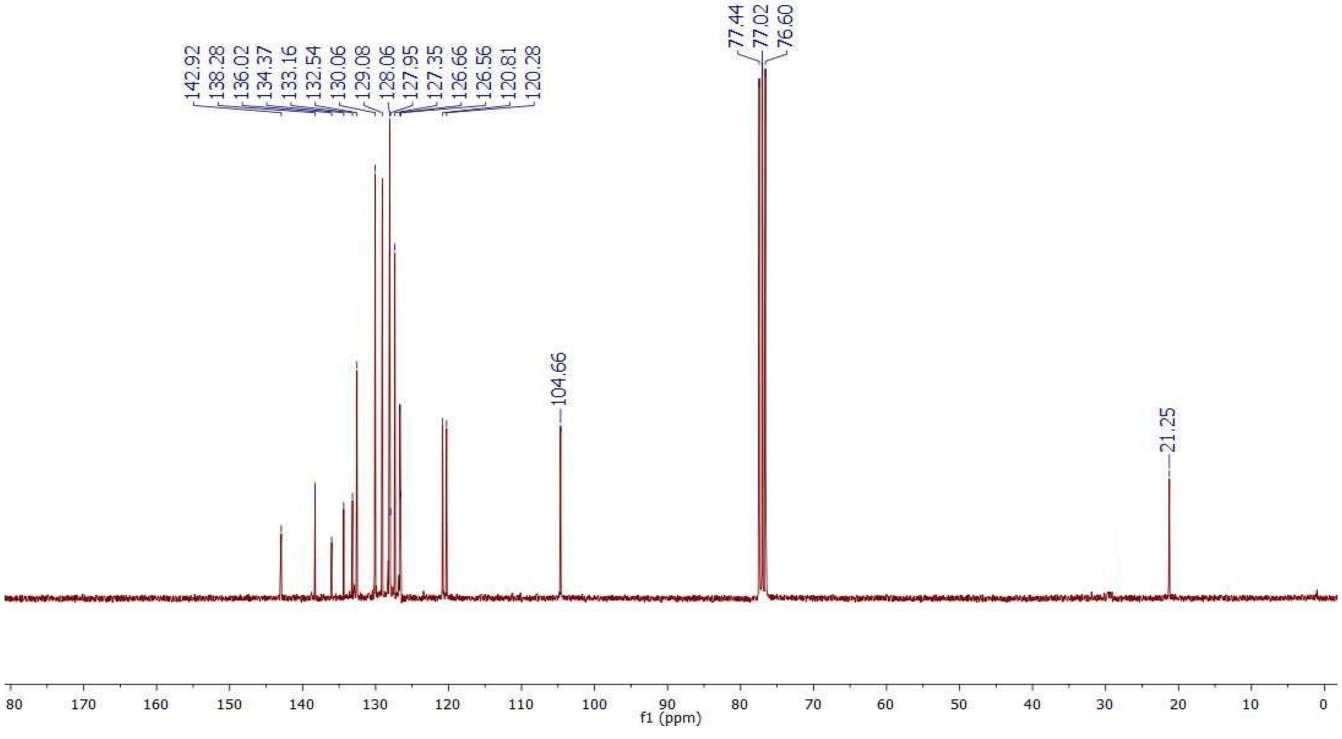
142.92
138.28
136.02
134.37
133.16
132.54
130.06
129.08
128.06
127.95
127.35
126.66
126.56
120.81
120.28
77.44
77.02
76.60
104.66
21.25
f1 (ppm)

6b

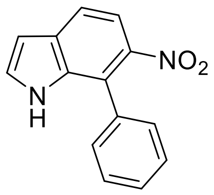
NO2
N
H

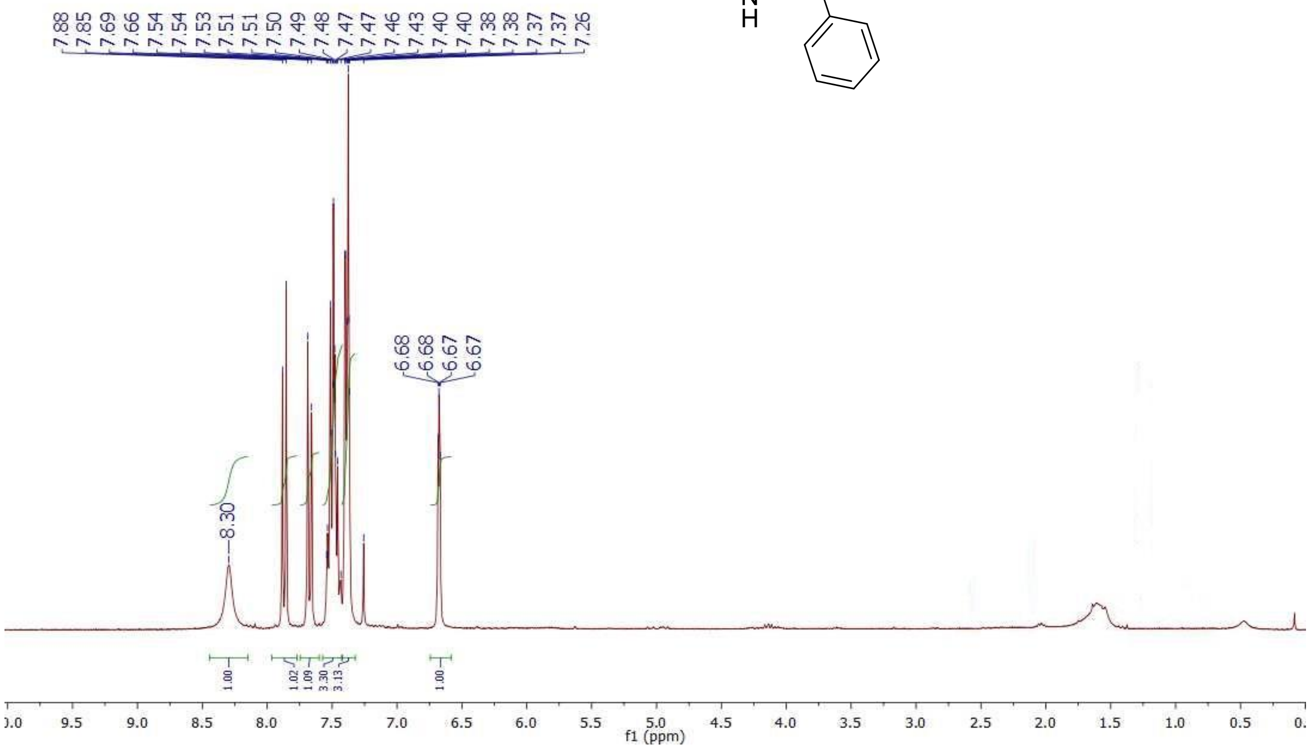
7.88 7.85 7.69 7.66 7.54 7.54 7.53 7.51 7.51 7.50 7.49 7.48 7.47 7.47 7.46 7.43 7.40 7.40 7.38 7.38 7.37 7.37 7.26
6.68 6.68 6.67 6.67
8.30
1.00 1.02 1.09 3.30 3.13 1.00
f1 (ppm)

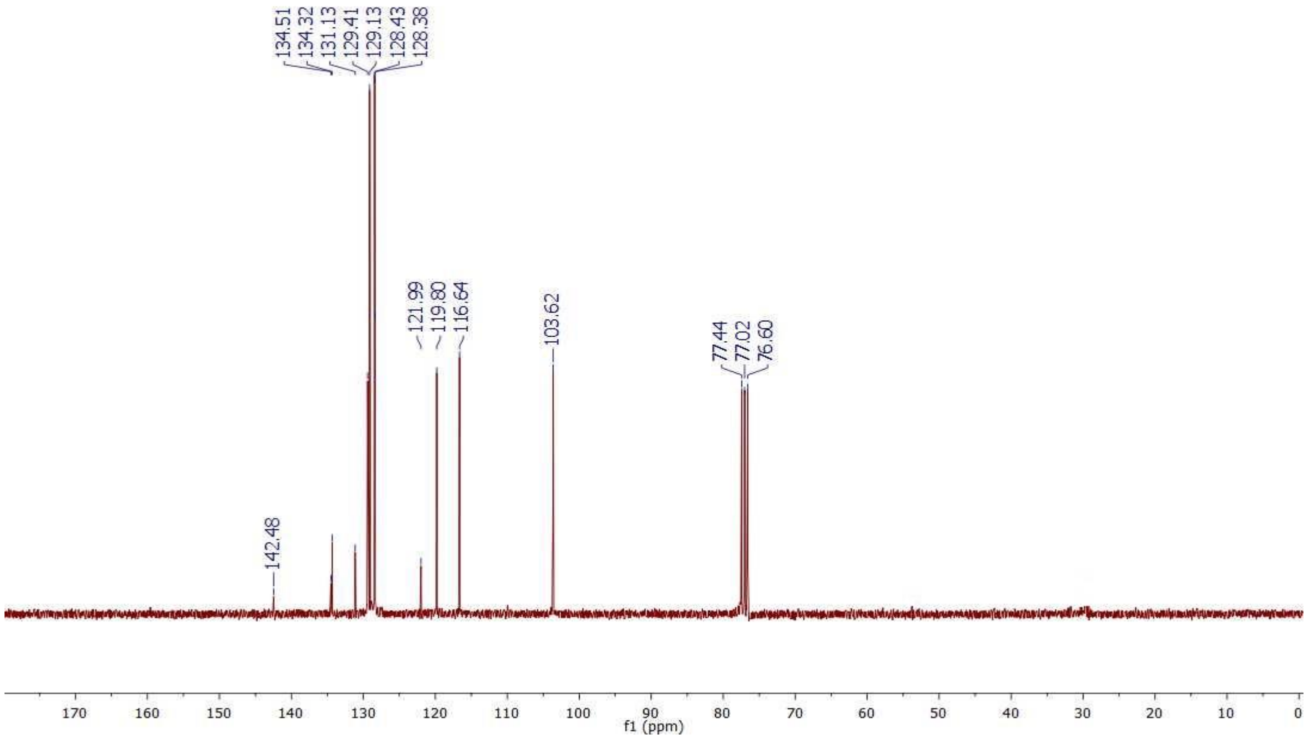
134.51 134.32 131.13 129.41 129.13 128.43 128.38
121.99 119.80 116.64
103.62
77.44 77.02 76.60
142.48
f1 (ppm)

6c

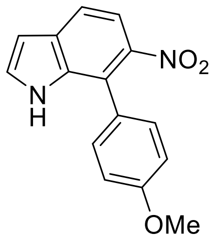
NO2
N
H
OMe

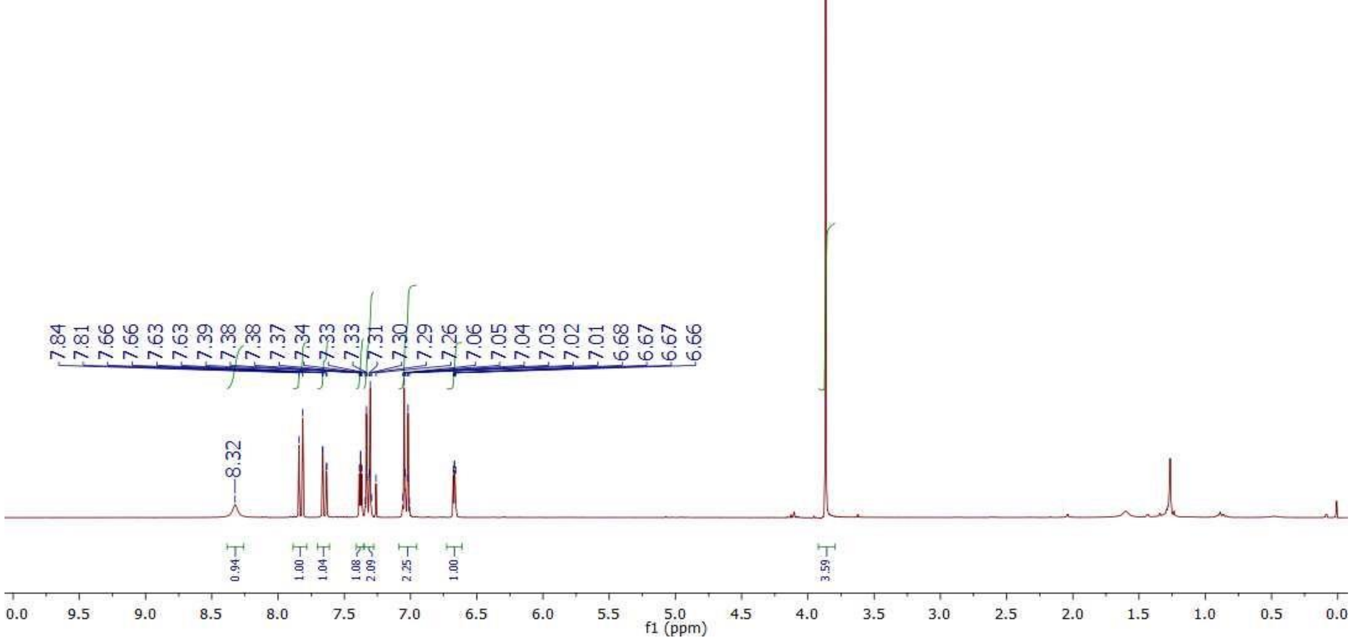
3.87
7.84
7.81
7.66
7.66
7.63
7.63
7.39
7.38
7.38
7.37
7.34
7.33
7.33
7.31
7.30
7.29
7.26
7.06
7.05
7.04
7.03
7.02
7.01
6.68
6.67
6.67
6.66
8.32
0.94
1.00
1.04
1.08
2.09
2.25
1.00
3.59
f1 (ppm)

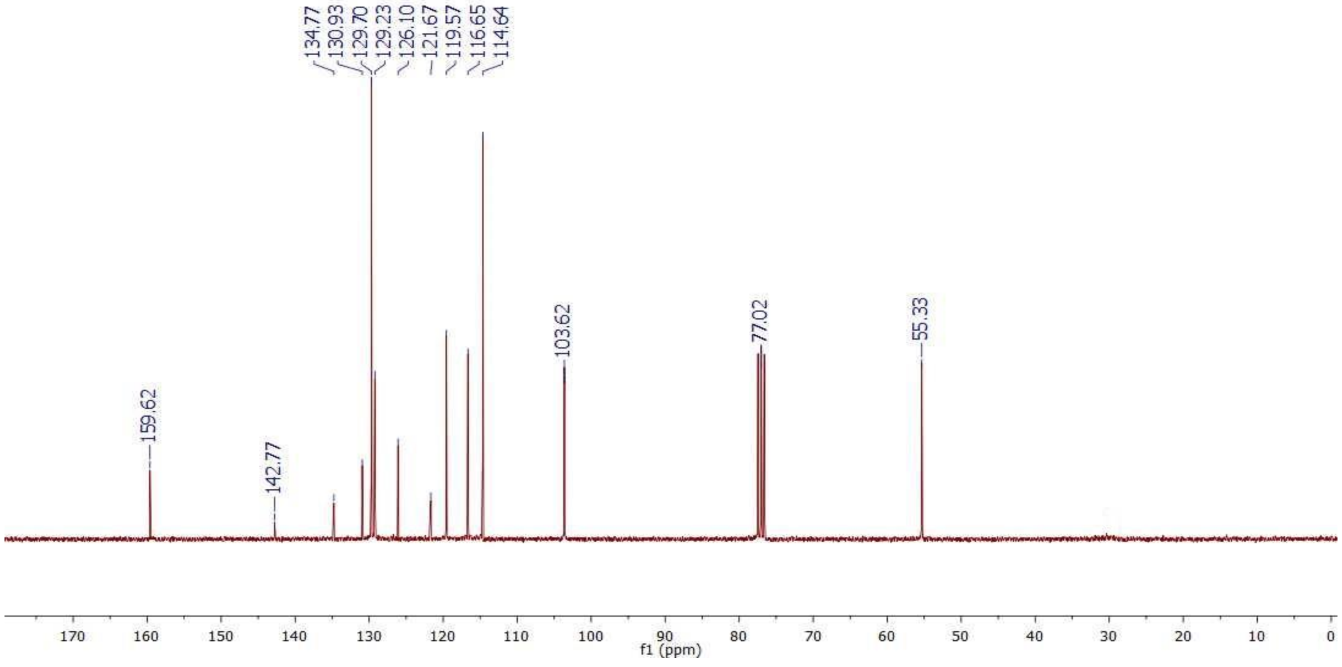
134.77
130.93
129.70
129.23
126.10
121.67
119.57
116.65
114.64
159.62
142.77
103.62
77.02
55.33
f1 (ppm)

6d

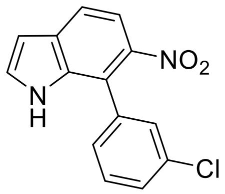
NO2
N
H
Cl

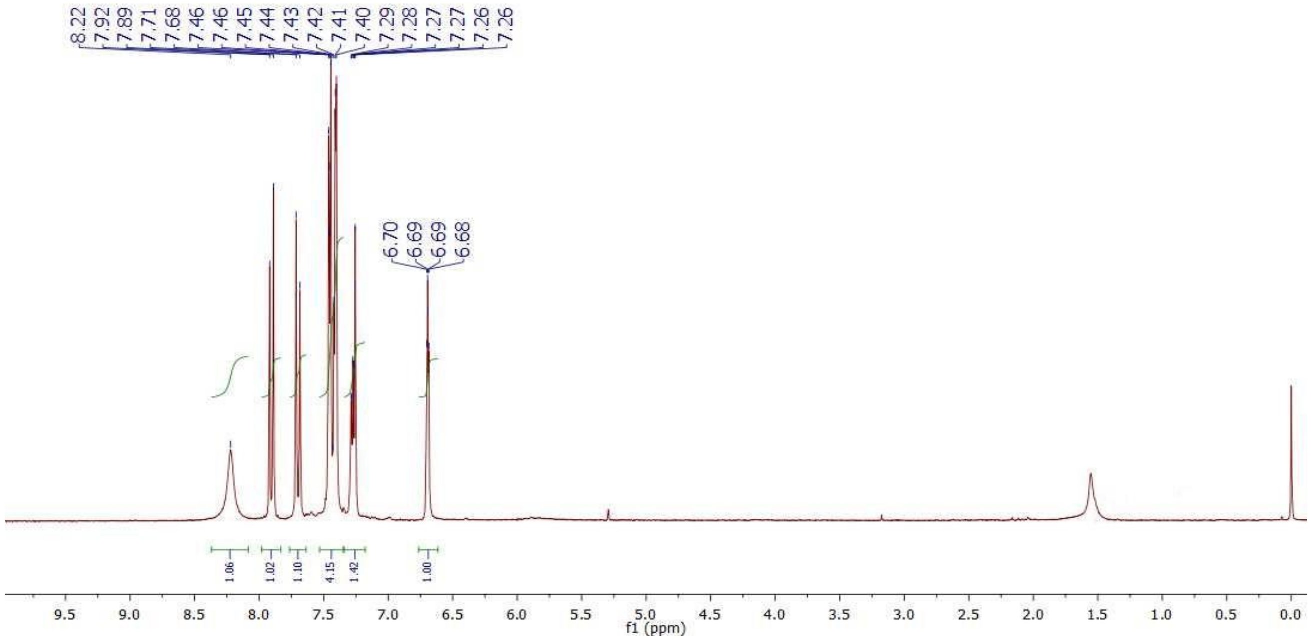
8.22 7.92 7.89 7.71 7.68 7.46 7.46 7.45 7.44 7.43 7.42 7.41 7.40 7.29 7.28 7.27 7.27 7.26 7.26
6.70 6.69 6.69 6.68
1.06 1.02 1.10 4.15 1.42 1.00
9.5 9.0 8.5 8.0 7.5 7.0 6.5 6.0 5.5 5.0 4.5 4.0 3.5 3.0 2.5 2.0 1.5 1.0 0.5 0.0
f1 (ppm)

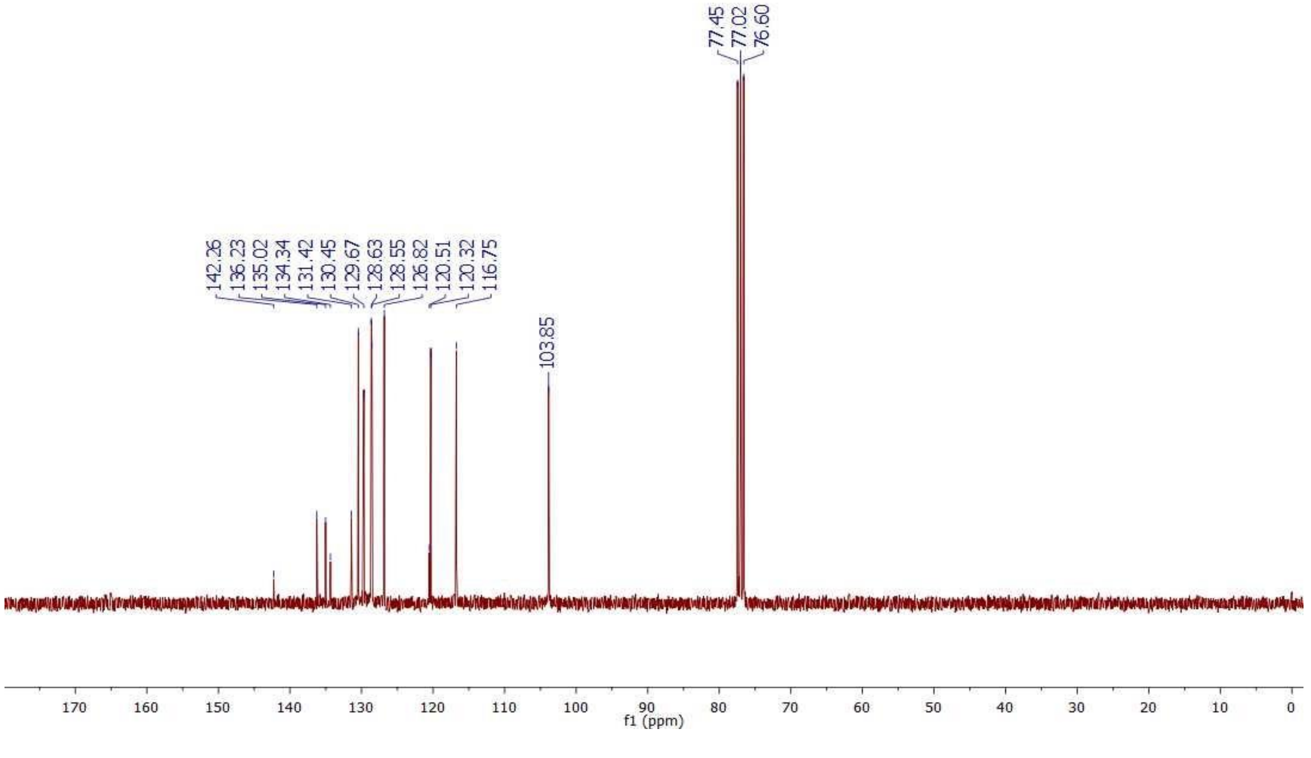
77.45 77.02 76.60
142.26 136.23 135.02 134.34 131.42 130.45 129.67 128.63 128.55 126.82 120.51 120.32 116.75
103.85
170 160 150 140 130 120 110 100 90 80 70 60 50 40 30 20 10 0
f1 (ppm)

6e

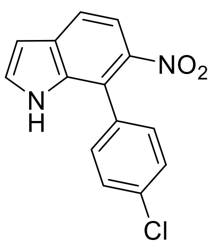

NO2
N
H
Cl

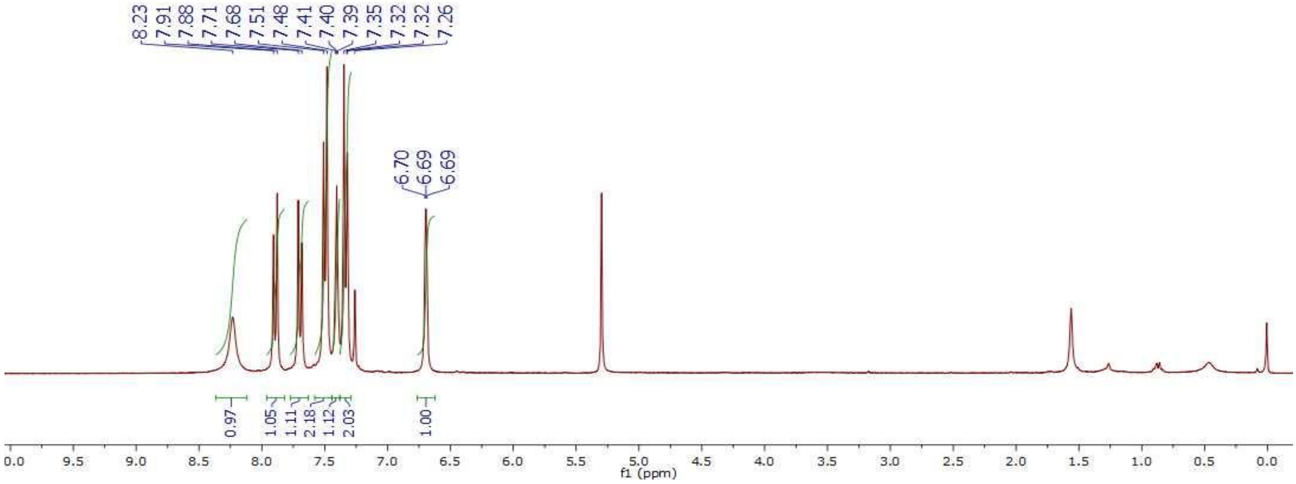

8.23
7.91
7.88
7.71
7.68
7.51
7.48
7.41
7.40
7.39
7.35
7.32
7.32
7.26
6.70
6.69
6.69
0.97
1.05
1.11
2.18
1.12
2.03
1.00
f1 (ppm)

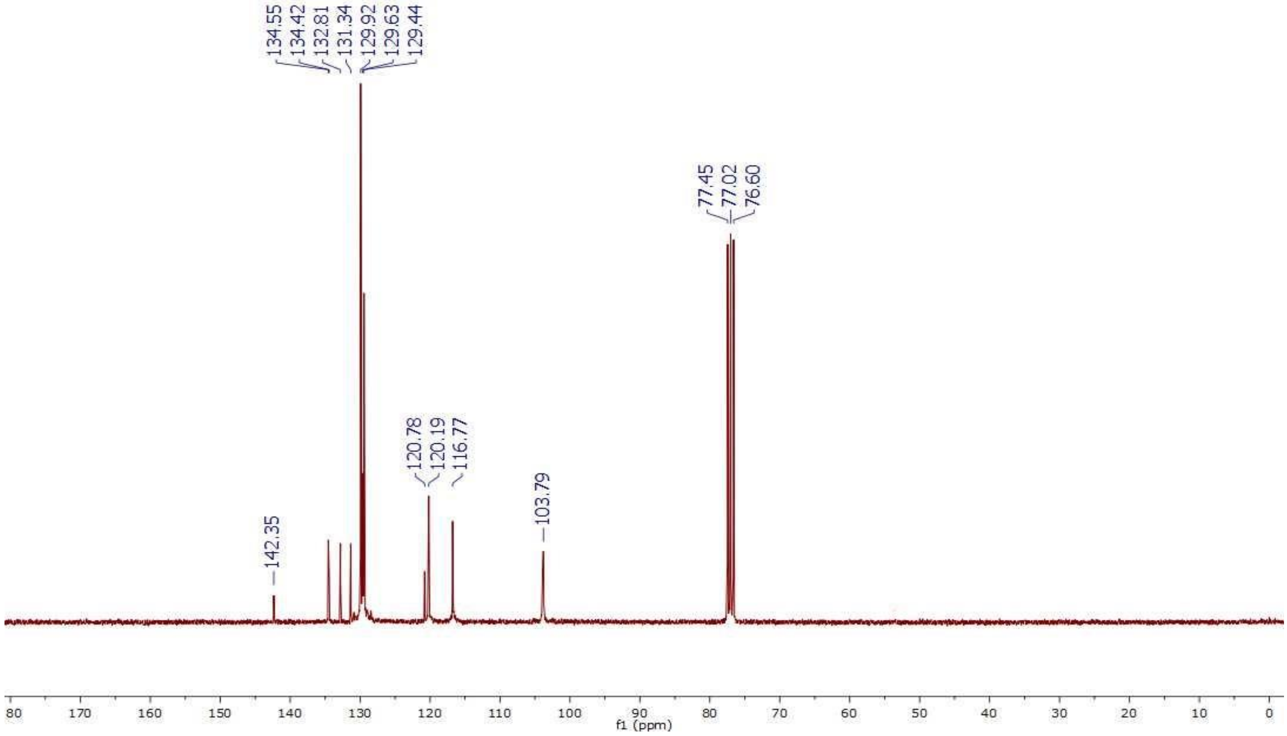

134.55
134.42
132.81
131.34
129.92
129.63
129.44
77.45
77.02
76.60
120.78
120.19
116.77
103.79
142.35
f1 (ppm)

6f

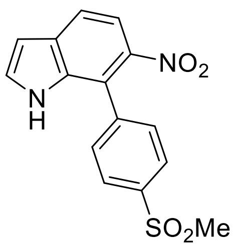
NO2
N
H
SO2Me

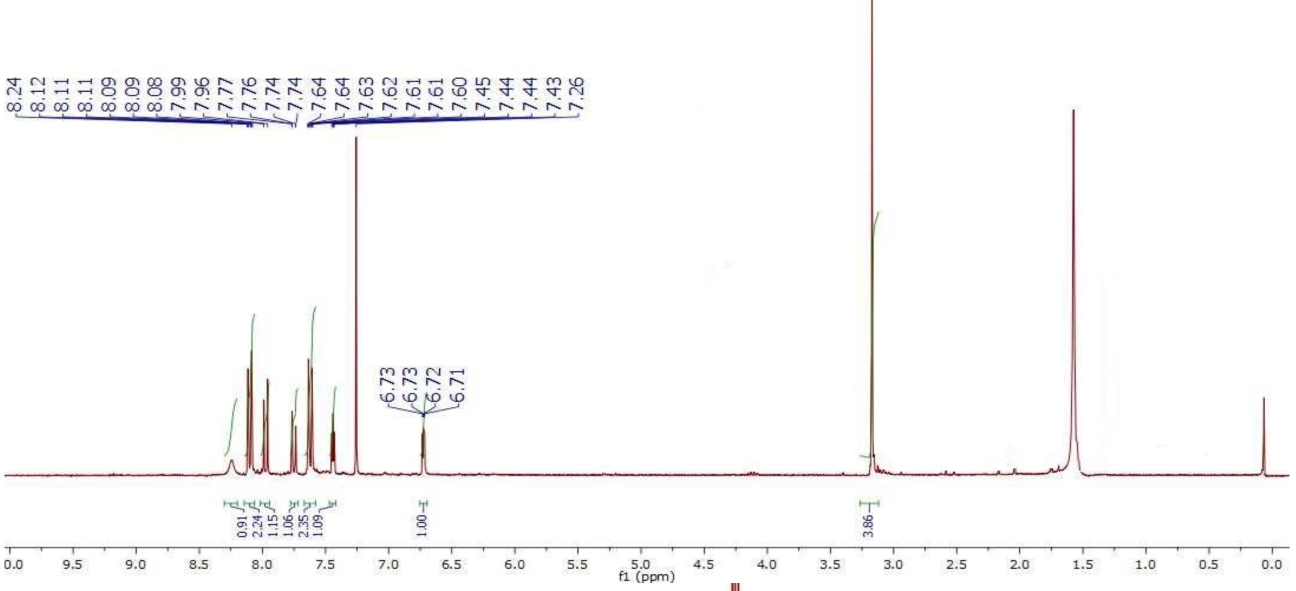
8.24
8.12
8.11
8.11
8.09
8.09
8.08
7.99
7.96
7.77
7.76
7.74
7.74
7.64
7.64
7.63
7.62
7.61
7.61
7.60
7.45
7.44
7.44
7.43
7.26
6.73
6.73
6.72
6.71
3.17
0.91
2.24
1.15
1.06
2.35
1.09
1.00
3.86
f1 (ppm)

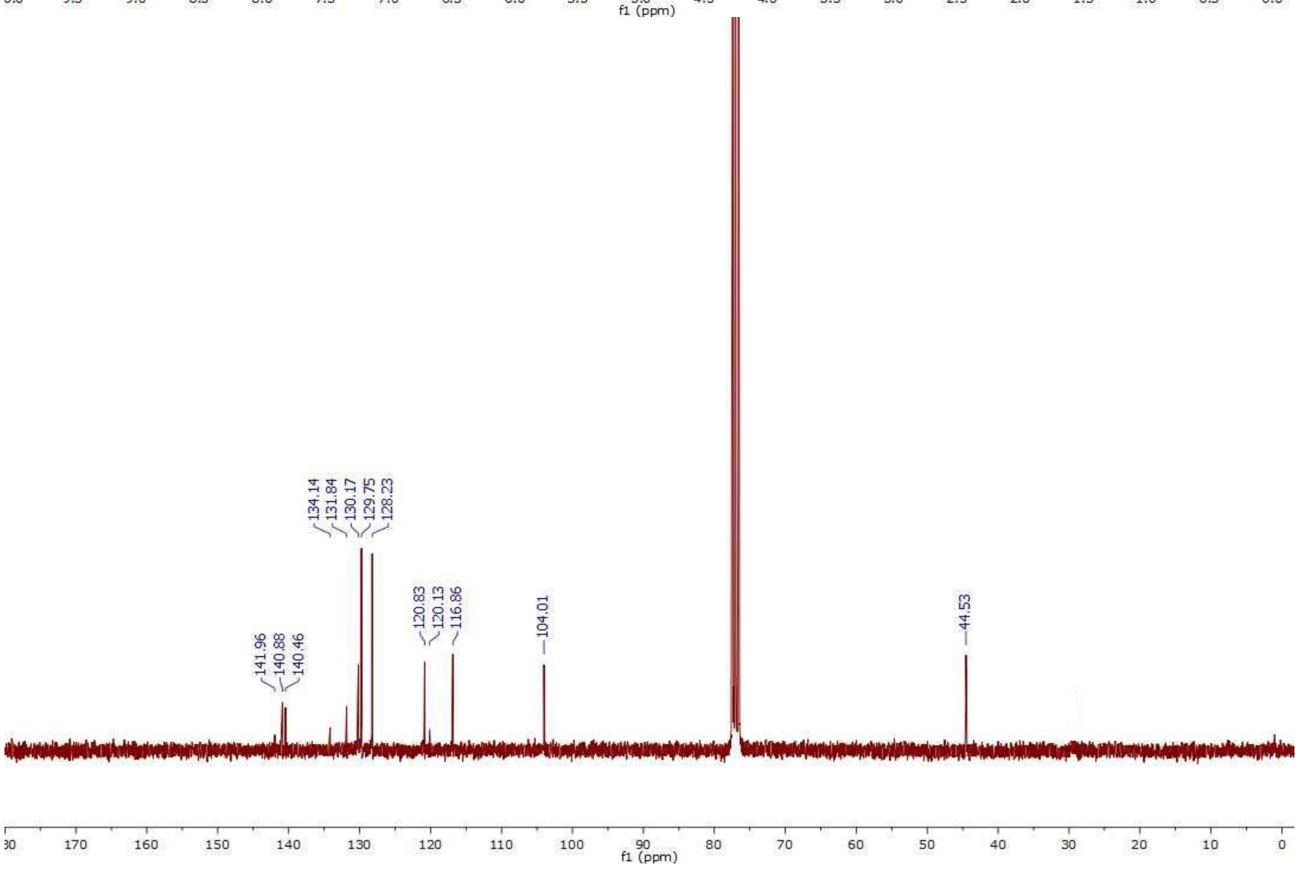
134.14
131.84
130.17
129.75
128.23
141.96
140.88
140.46
120.83
120.13
116.86
104.01
44.53
f1 (ppm)

6g

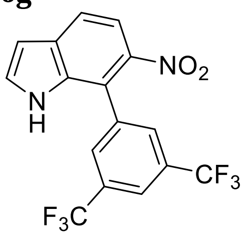
NO2
N
H
CF3
F3C

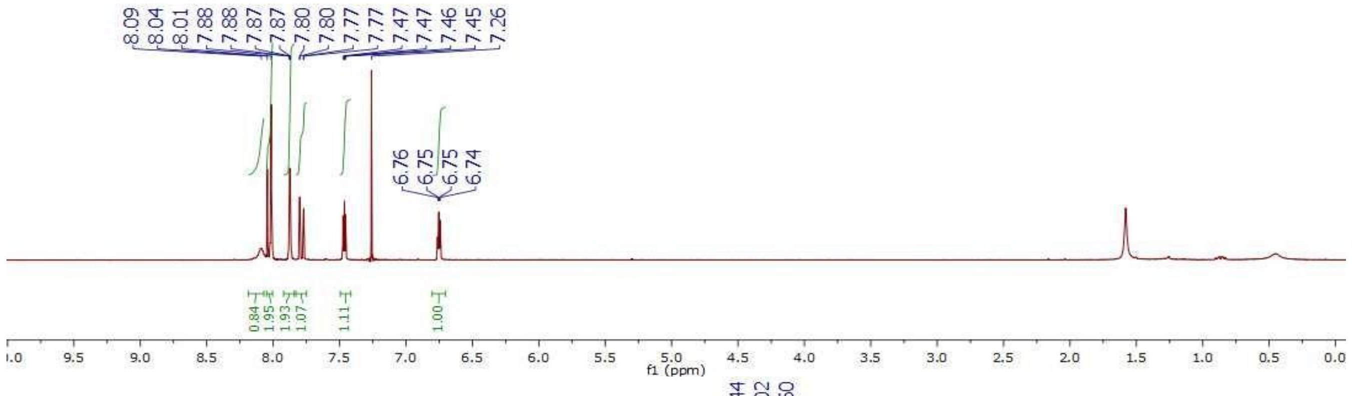
8.09
8.04
8.01
7.88
7.88
7.87
7.87
7.80
7.80
7.77
7.77
7.47
7.47
7.46
7.45
7.26
6.76
6.75
6.75
6.74
0.84
1.95
1.93
1.07
1.11
1.00
f1 (ppm)

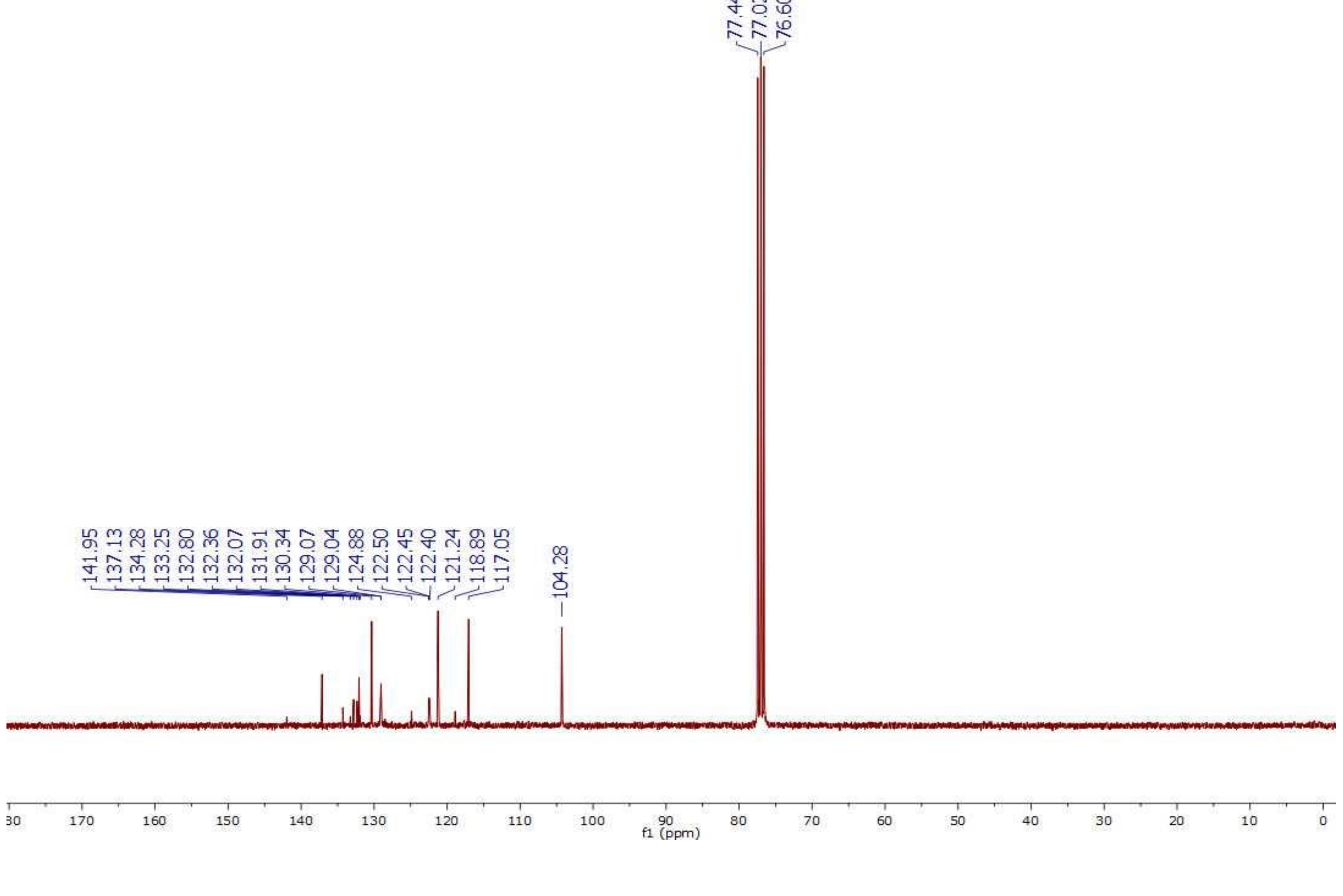
77.44
77.02
76.60
141.95
137.13
134.28
133.25
132.80
132.36
132.07
131.91
130.34
129.07
129.04
124.88
122.50
122.45
122.40
121.24
118.89
117.05
104.28
f1 (ppm)

9g

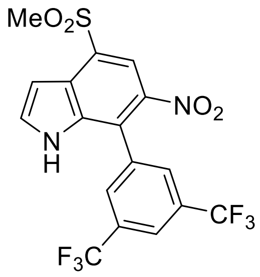
MeO2S
NO2
N
H
CF3
F3C

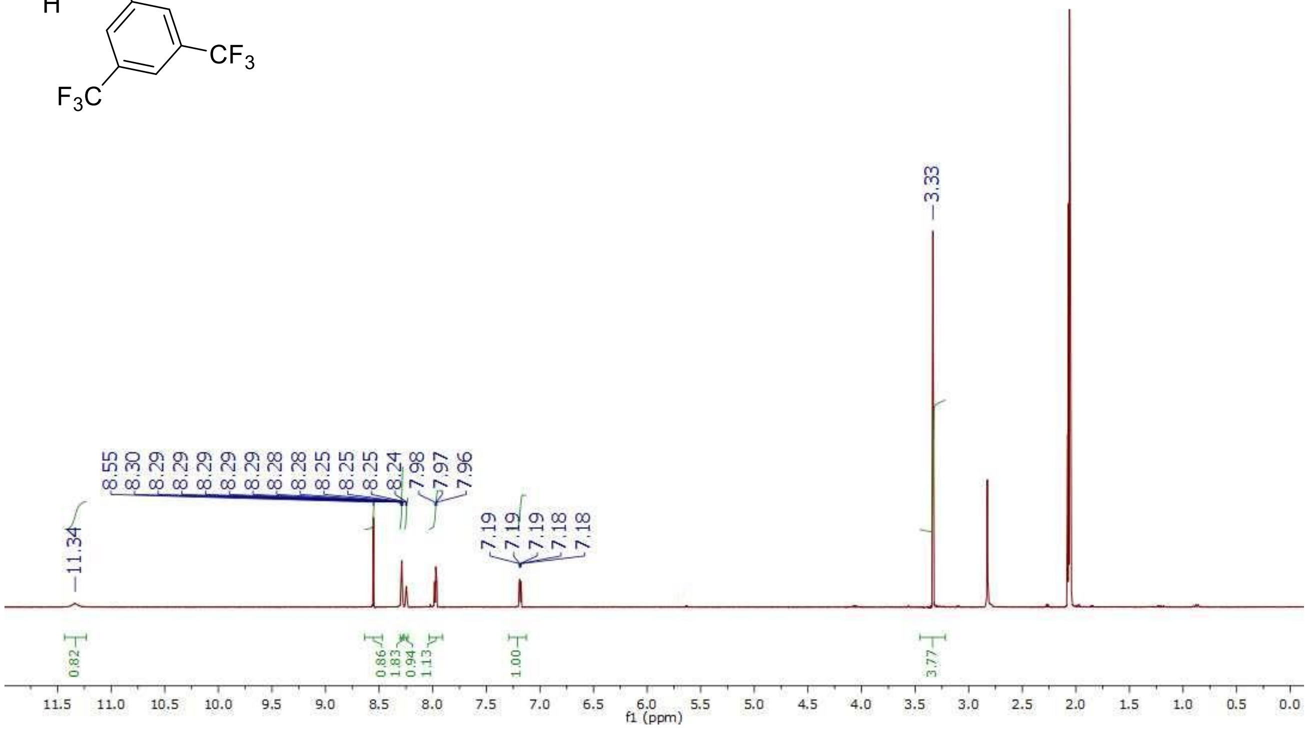
8.55 8.30 8.29 8.29 8.29 8.29 8.29 8.28 8.28 8.25 8.25 8.25 8.24 7.98 7.97 7.96
11.34
7.19 7.19 7.19 7.18 7.18
3.33
0.82 0.86 1.83 0.94 1.13 1.00 3.77
11.5 11.0 10.5 10.0 9.5 9.0 8.5 8.0 7.5 7.0 6.5 6.0 5.5 5.0 4.5 4.0 3.5 3.0 2.5 2.0 1.5 1.0 0.5 0.0
f1 (ppm)

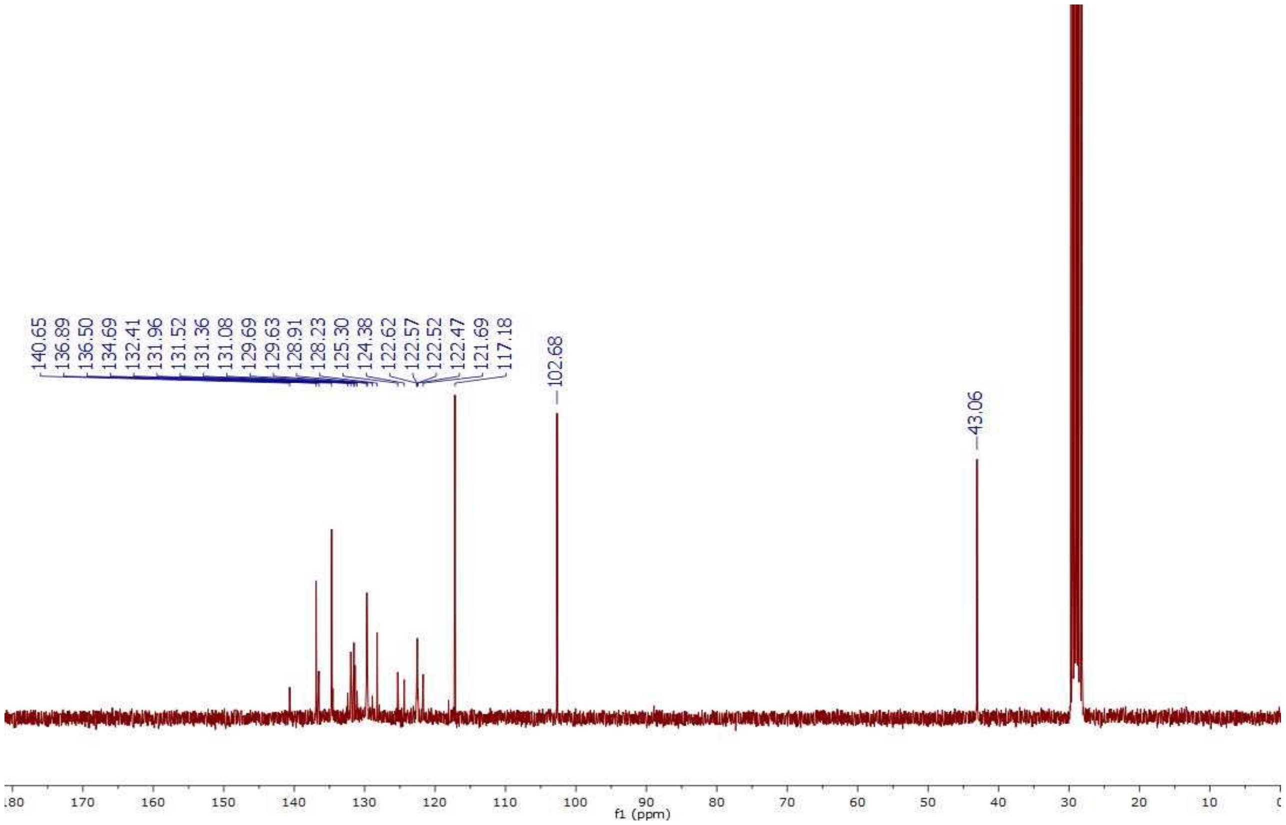
140.65 136.89 136.50 134.69 132.41 131.96 131.52 131.36 131.08 129.69 129.63 128.91 128.23 125.30 124.38 122.62 122.57 122.52 122.47 121.69 117.18
102.68
43.06
170 160 150 140 130 120 110 100 90 80 70 60 50 40 30 20 10
f1 (ppm)

6h

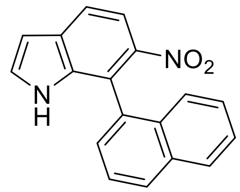
NO2
N
H

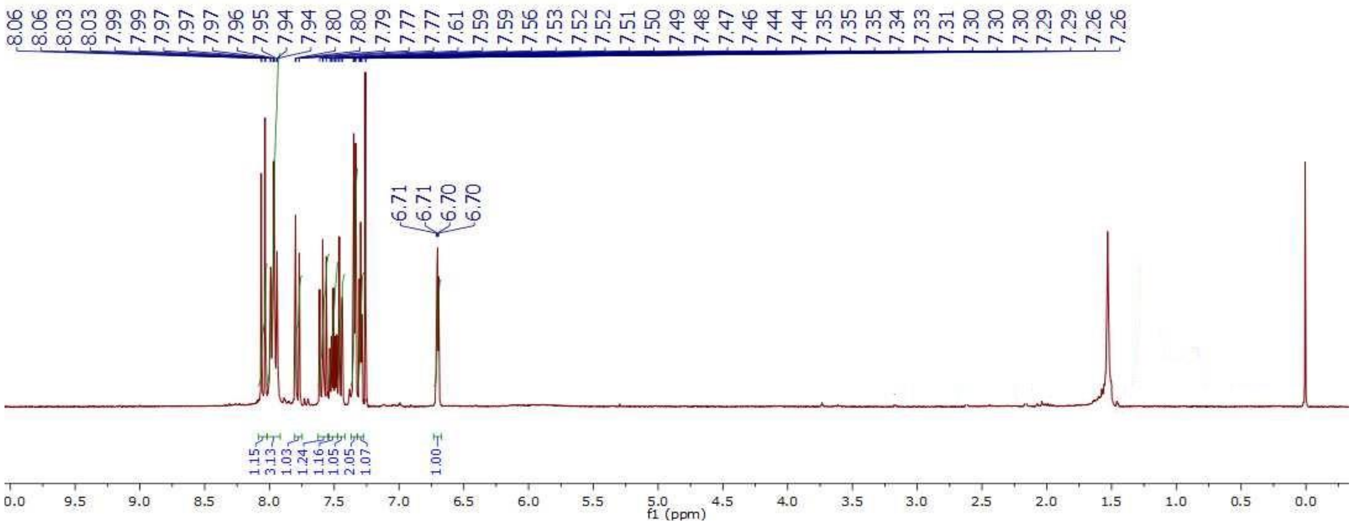
8.06
8.06
8.03
8.03
7.99
7.99
7.97
7.97
7.97
7.96
7.95
7.94
7.94
7.80
7.80
7.79
7.77
7.77
7.61
7.59
7.59
7.56
7.53
7.52
7.52
7.51
7.50
7.49
7.48
7.47
7.46
7.44
7.44
7.35
7.35
7.35
7.34
7.33
7.31
7.30
7.30
7.30
7.29
7.29
7.26
7.26
6.71
6.71
6.70
6.70
1.15
3.13
1.03
1.24
1.16
1.05
2.05
1.07
1.00
f1 (ppm)

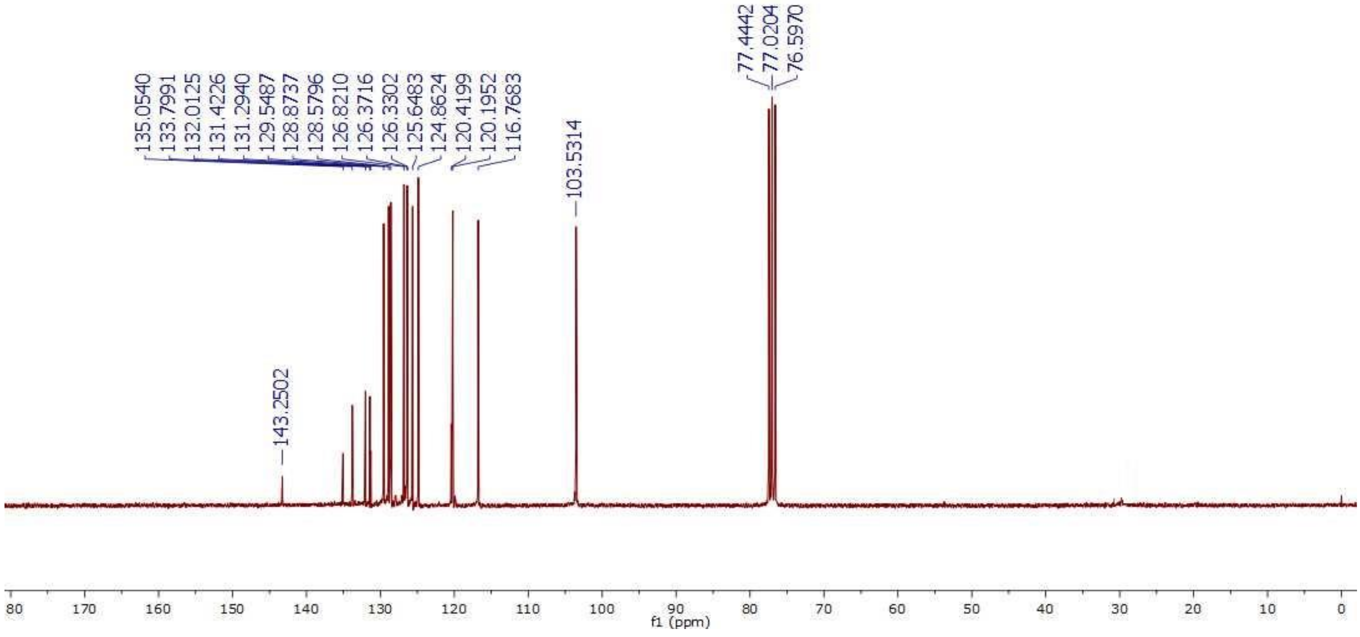
135.0540
133.7991
132.0125
131.4226
131.2940
129.5487
128.8737
128.5796
126.8210
126.3716
126.3302
125.6483
124.8624
120.4199
120.1952
116.7683
103.5314
143.2502
77.4442
77.0204
76.5970
f1 (ppm)

6i

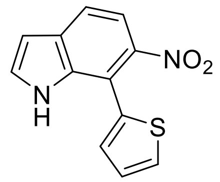

NO2
N
H
S

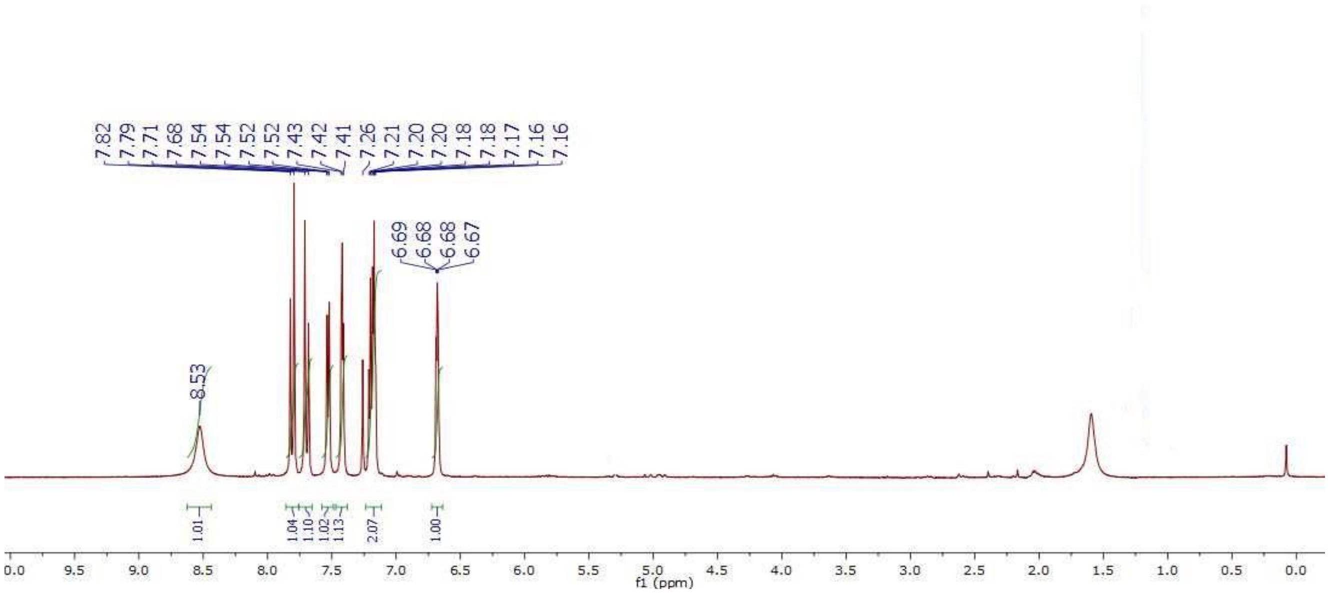

7.82
7.79
7.71
7.68
7.54
7.54
7.52
7.52
7.43
7.42
7.41
7.26
7.21
7.20
7.20
7.18
7.18
7.17
7.16
7.16
6.69
6.68
6.68
6.67
8.53
1.01
1.04
1.10
1.02
1.13
2.07
1.00
f1 (ppm)

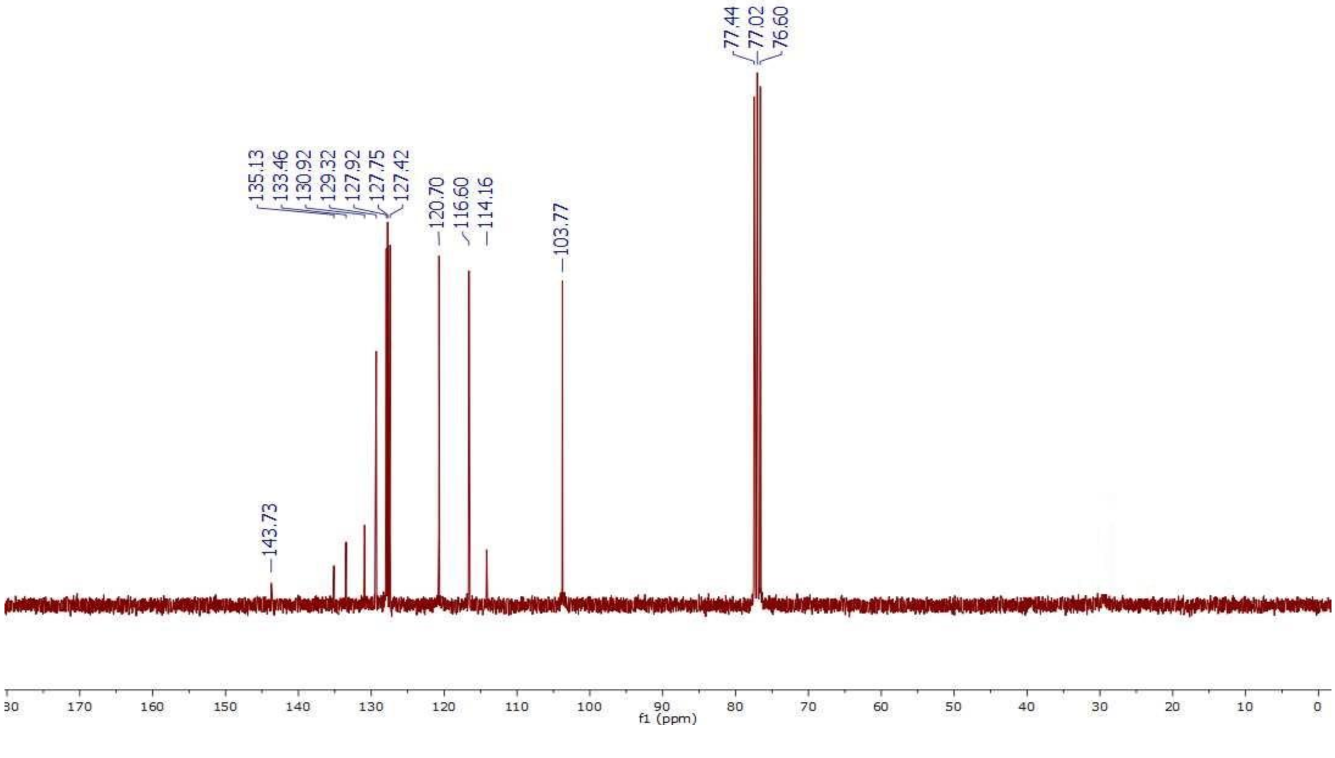

77.44
77.02
76.60
135.13
133.46
130.92
129.32
127.92
127.75
127.42
120.70
116.60
114.16
103.77
143.73
f1 (ppm)

10a

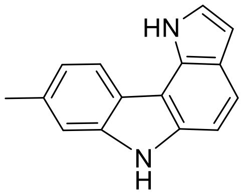
HN
N
H

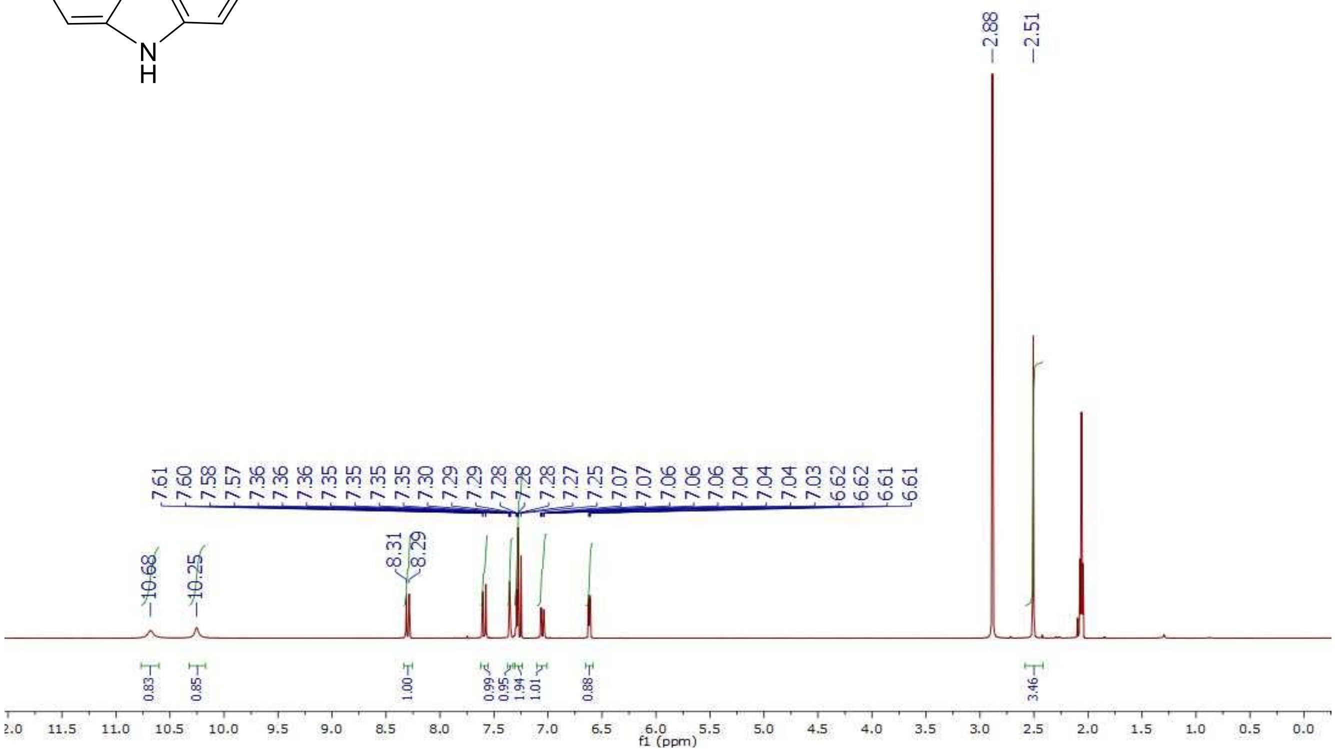
2.88
2.51
7.61 7.60 7.58 7.57 7.36 7.36 7.36 7.35 7.35 7.35 7.35 7.30 7.29 7.29 7.28 7.28 7.28 7.27 7.25 7.07 7.07 7.06 7.06 7.06 7.04 7.04 7.04 7.03 6.62 6.62 6.61 6.61
10.68
10.25
8.31
8.29
0.83
0.85
1.00
0.99
0.95
1.94
1.01
0.88
3.46
f1 (ppm)

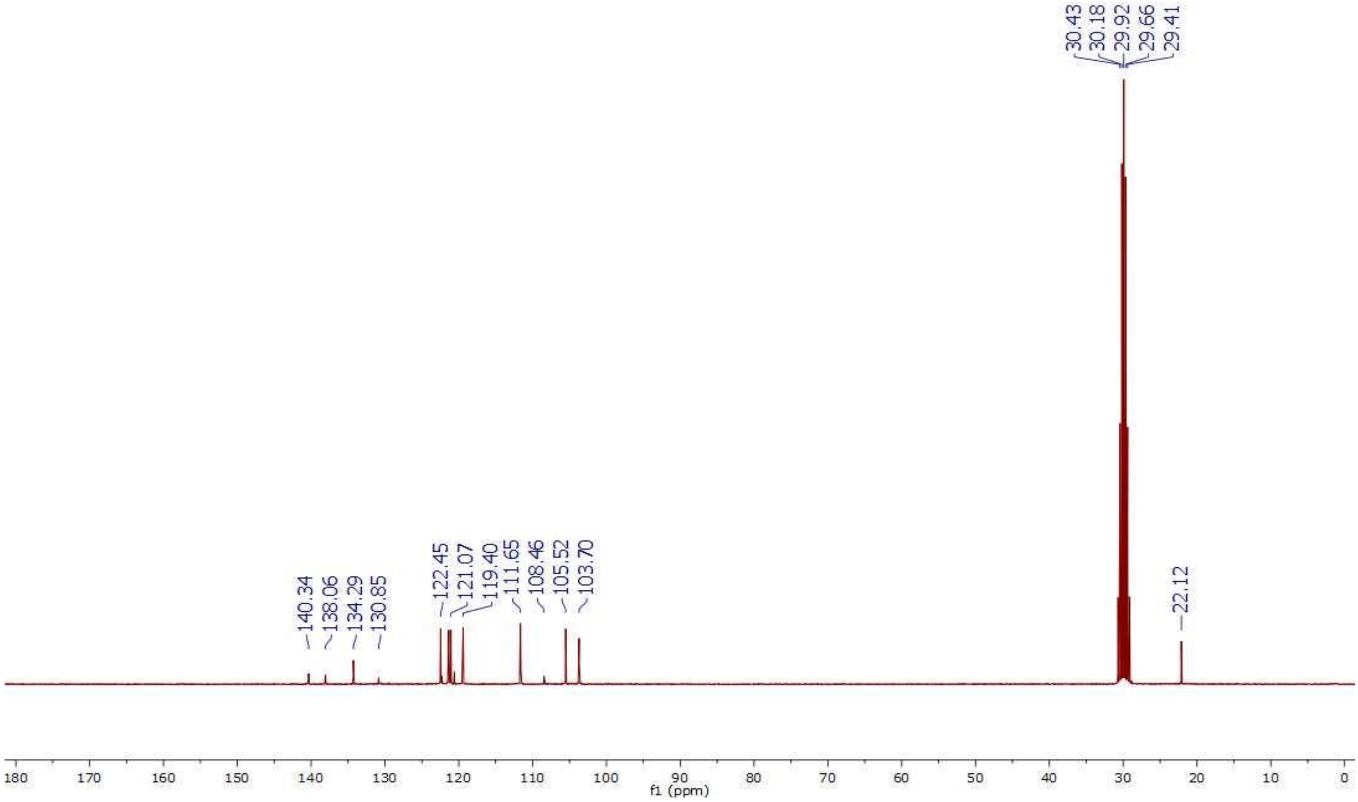
30.43
30.18
29.92
29.66
29.41
140.34
138.06
134.29
130.85
122.45
121.07
119.40
111.65
108.46
105.52
103.70
22.12
f1 (ppm)

10b

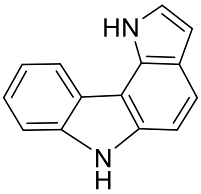
HN
N
H

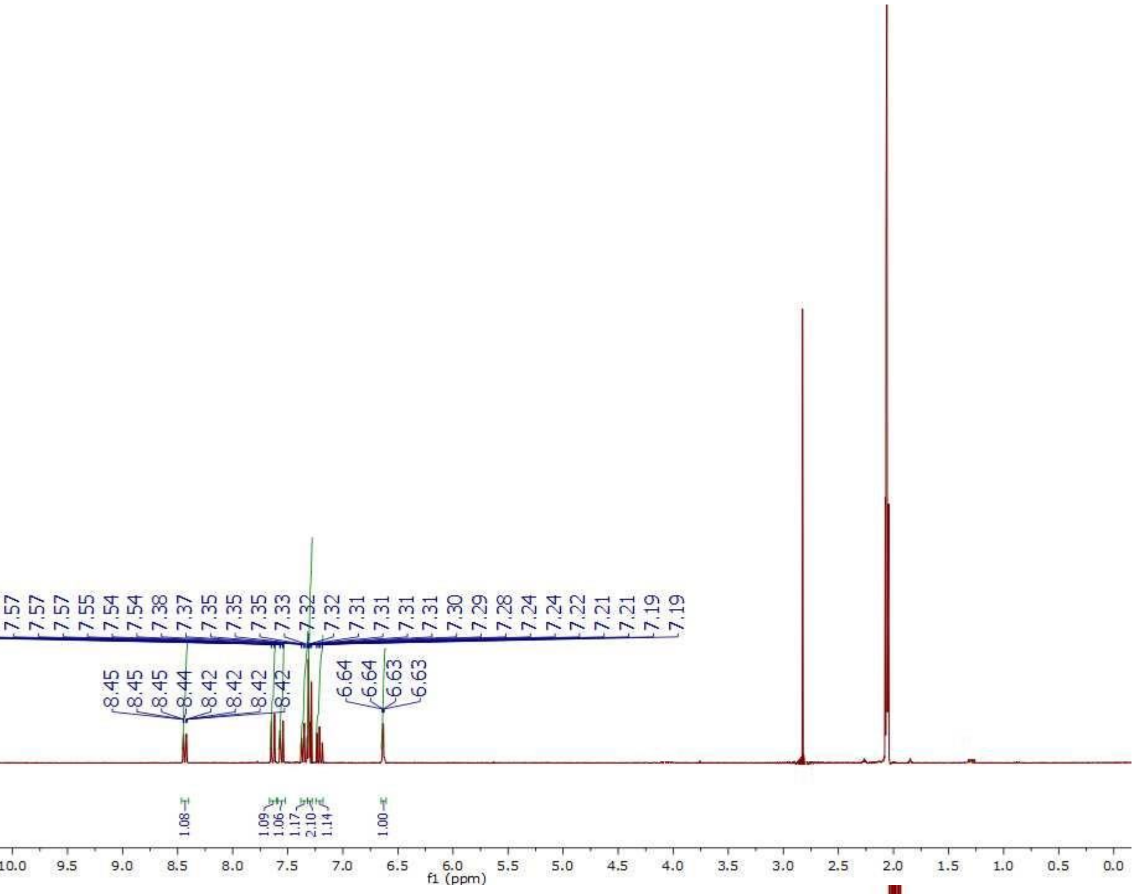
7.65 7.65 7.62 7.62 7.57 7.57 7.57 7.55 7.54 7.54 7.38 7.37 7.35 7.35 7.35 7.33 7.32 7.32 7.31 7.31 7.31 7.31 7.30 7.29 7.28 7.24 7.24 7.22 7.21 7.21 7.19 7.19
10.72
10.38
8.45 8.45 8.45 8.44 8.42 8.42 8.42 8.42
6.64 6.64 6.63 6.63
1.08
1.09 1.06 1.17 2.10 1.14
1.00
f1 (ppm)

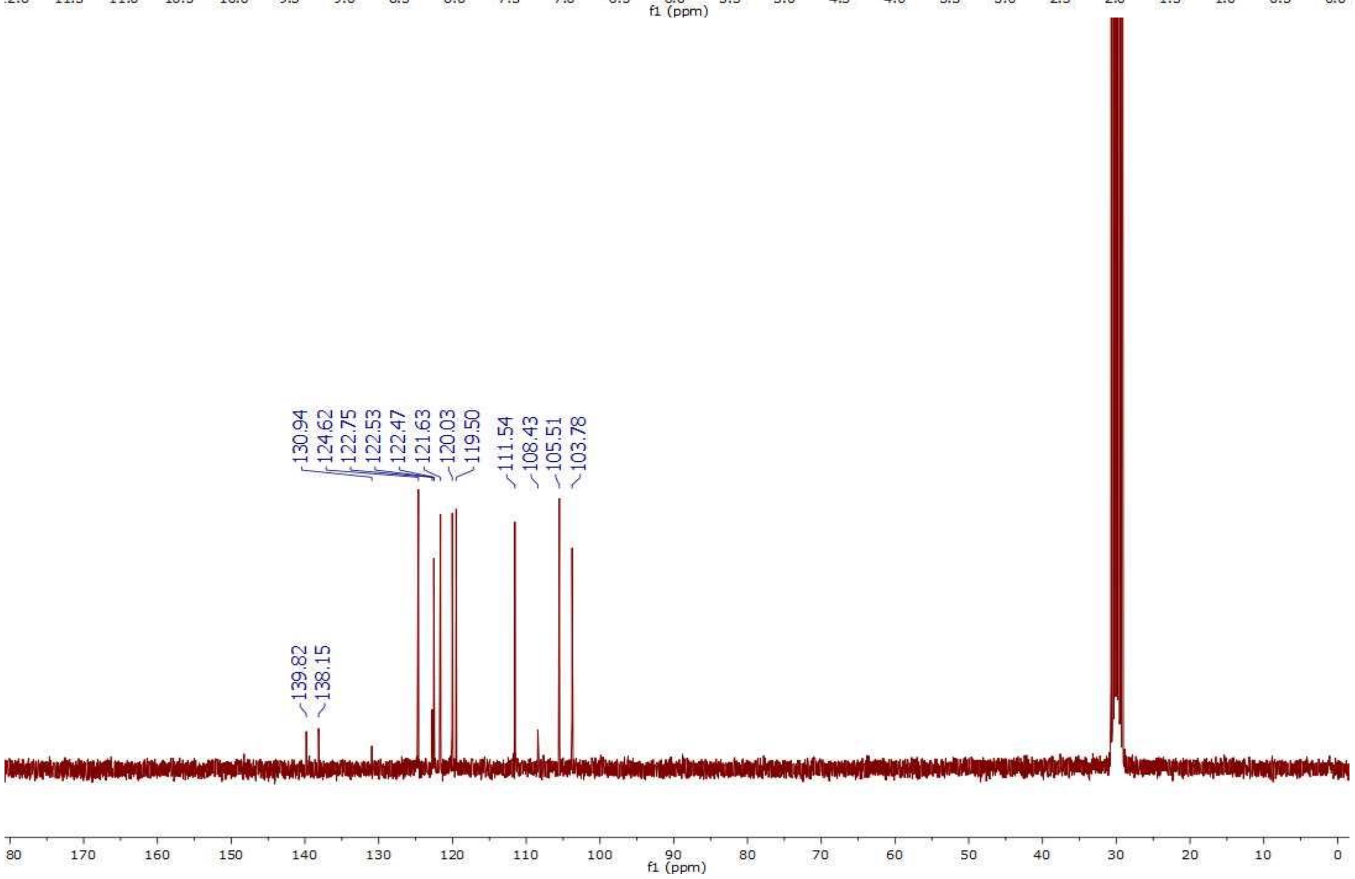
130.94 124.62 122.75 122.53 122.47 121.63 120.03 119.50
111.54 108.43 105.51 103.78
139.82 138.15
f1 (ppm)

10c

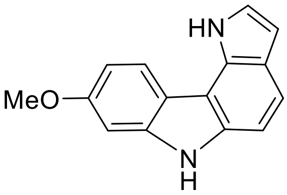
HN
MeO
N
H

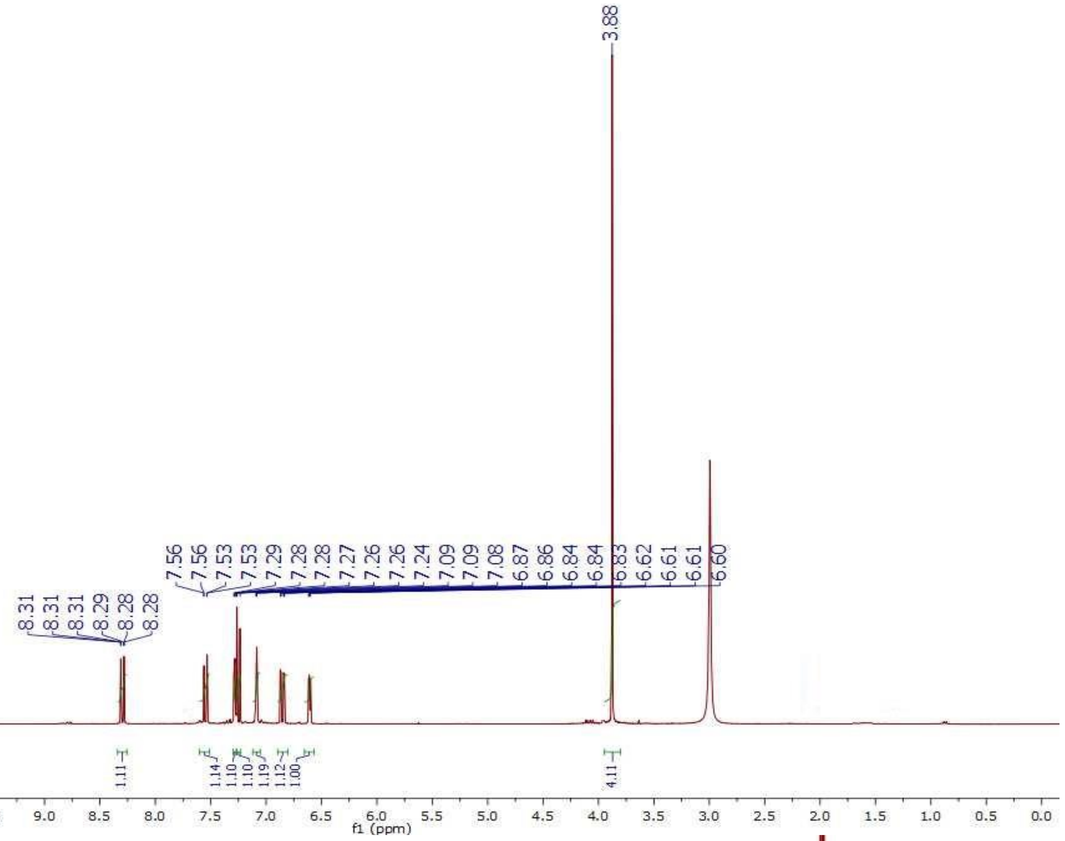
3.88
7.56
7.56
7.53
7.53
7.29
7.28
7.28
7.27
7.26
7.26
7.24
7.09
7.09
7.08
6.87
6.86
6.84
6.84
6.83
6.62
6.61
6.61
6.60
8.31
8.31
8.31
8.29
8.28
8.28
10.68
10.27
0.85
0.97
1.11
1.14
1.10
1.10
1.19
1.12
1.00
4.11
f1 (ppm)

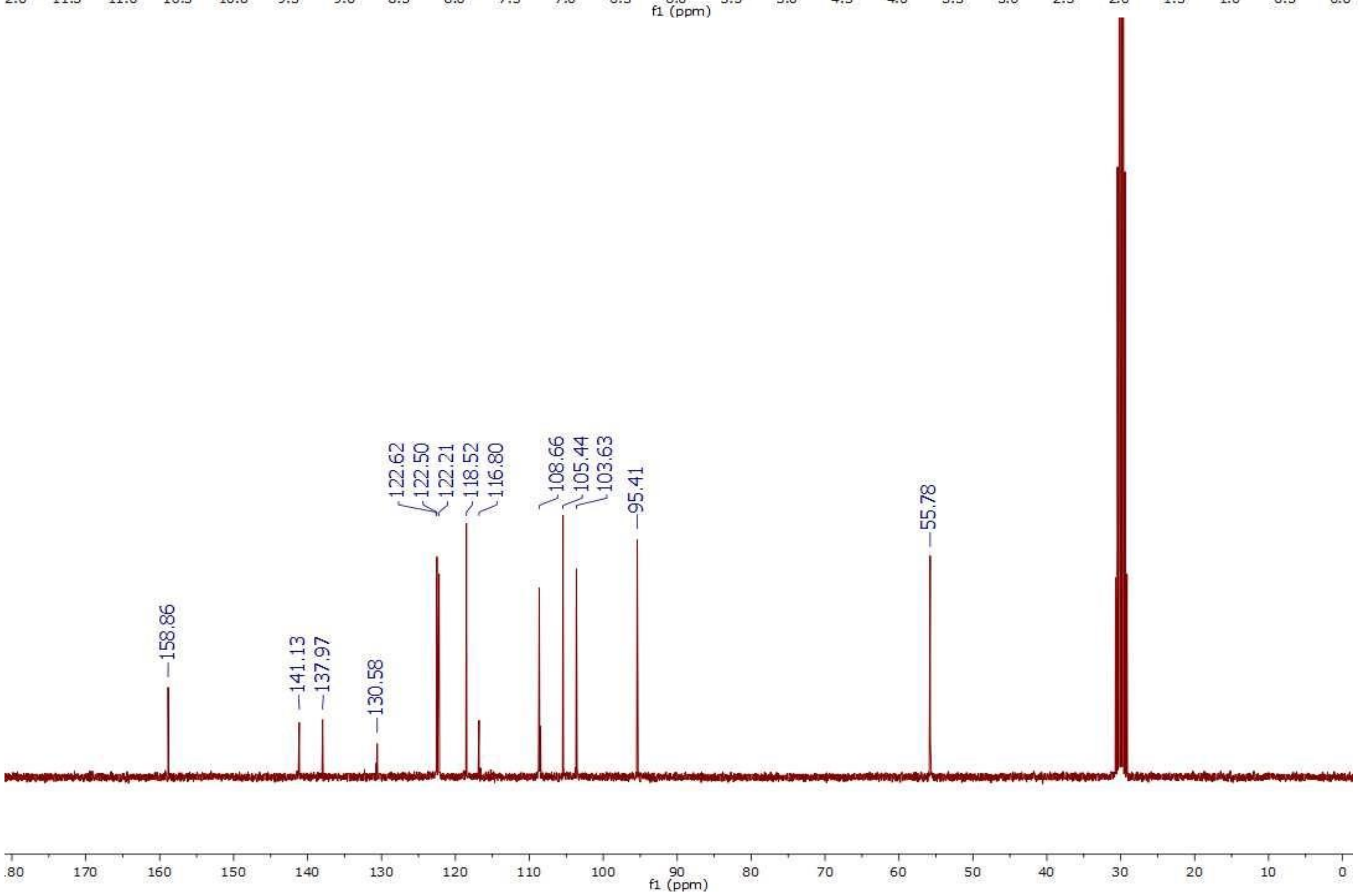
122.62
122.50
122.21
118.52
116.80
108.66
105.44
103.63
95.41
55.78
158.86
141.13
137.97
130.58
f1 (ppm)

10d

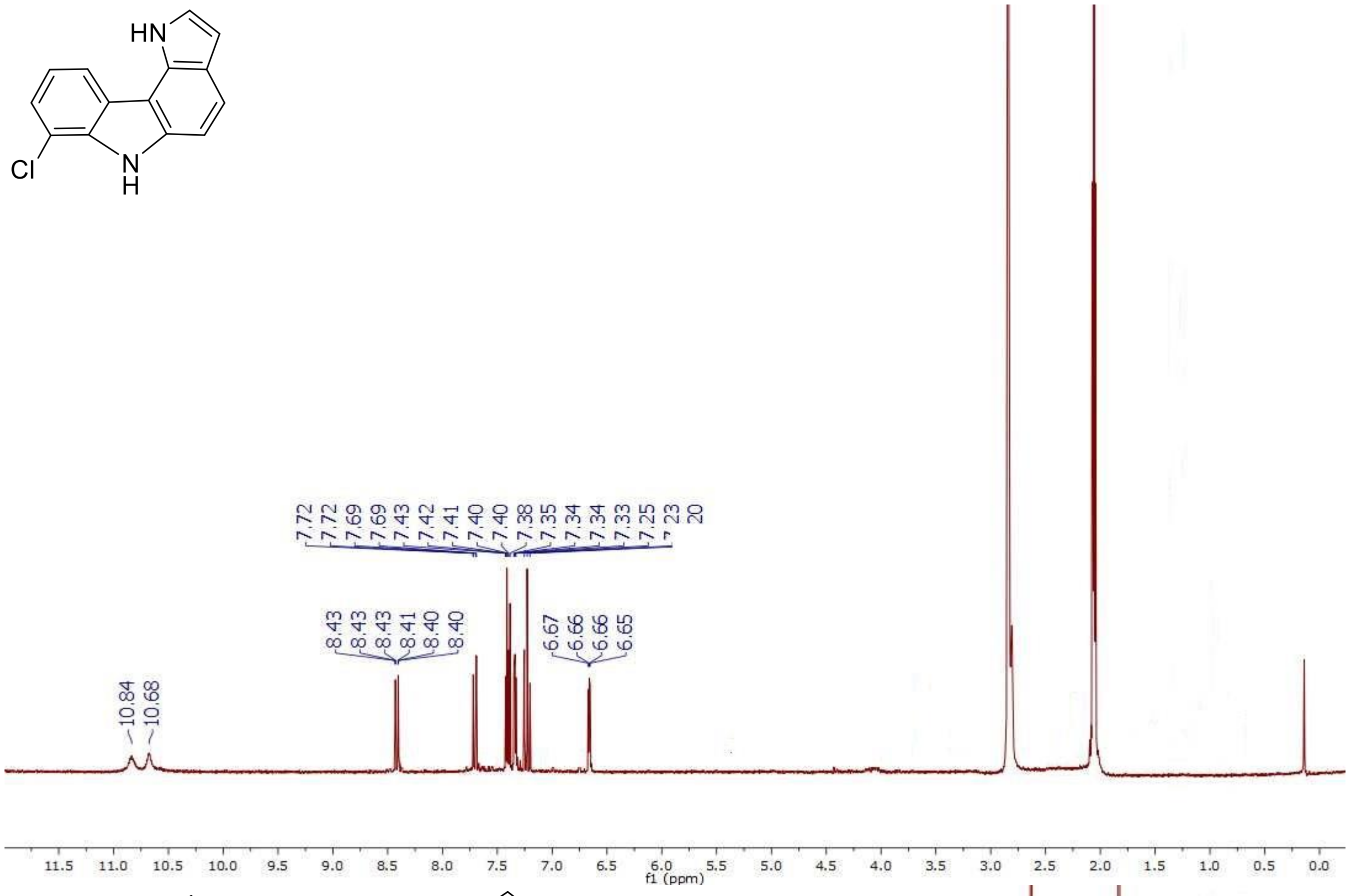
HN
Cl
N
H
7.72 7.72 7.69 7.69 7.43 7.42 7.41 7.40 7.40 7.38 7.35 7.34 7.34 7.33 7.25 7.23 7.20
8.43 8.43 8.43 8.41 8.40 8.40
6.67 6.66 6.66 6.65
10.84 10.68
11.5 11.0 10.5 10.0 9.5 9.0 8.5 8.0 7.5 7.0 6.5 6.0 5.5 5.0 4.5 4.0 3.5 3.0 2.5 2.0 1.5 1.0 0.5 0.0
f1 (ppm)

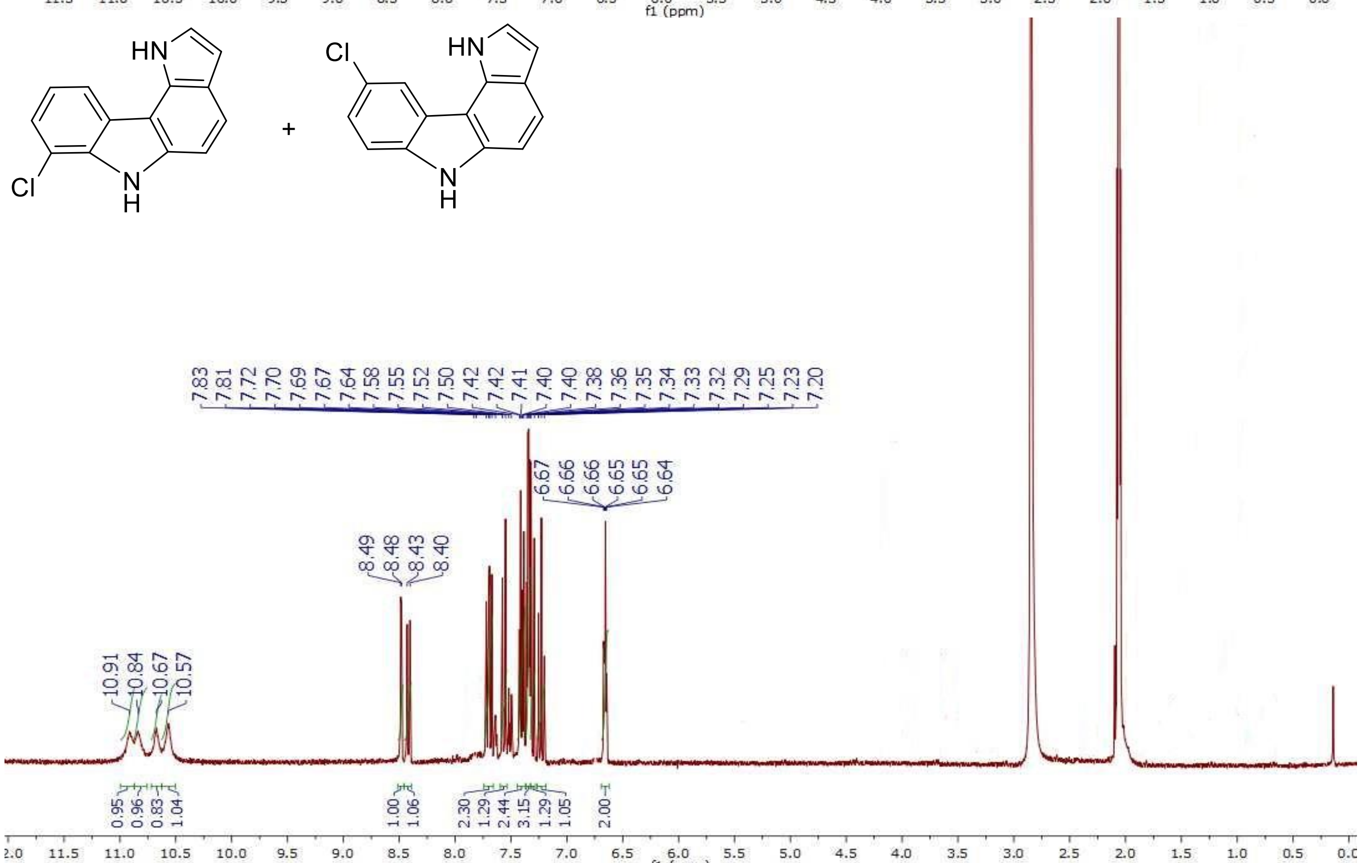
HN
Cl
N
H
+
Cl
HN
N
H
7.83 7.81 7.72 7.70 7.69 7.67 7.64 7.58 7.55 7.52 7.50 7.42 7.42 7.41 7.40 7.40 7.38 7.36 7.35 7.34 7.33 7.32 7.29 7.25 7.23 7.20
6.67 6.66 6.66 6.65 6.65 6.64
8.49 8.48 8.43 8.40
10.91 10.84 10.67 10.57
0.95 0.96 0.83 1.04
1.00 1.06
2.30 1.29 2.44 3.15 1.29 1.05
2.00
12.0 11.5 11.0 10.5 10.0 9.5 9.0 8.5 8.0 7.5 7.0 6.5 6.0 5.5 5.0 4.5 4.0 3.5 3.0 2.5 2.0 1.5 1.0 0.5 0.0
f1 (ppm)

10e

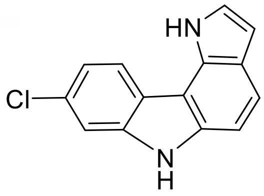
HN
Cl
N
H

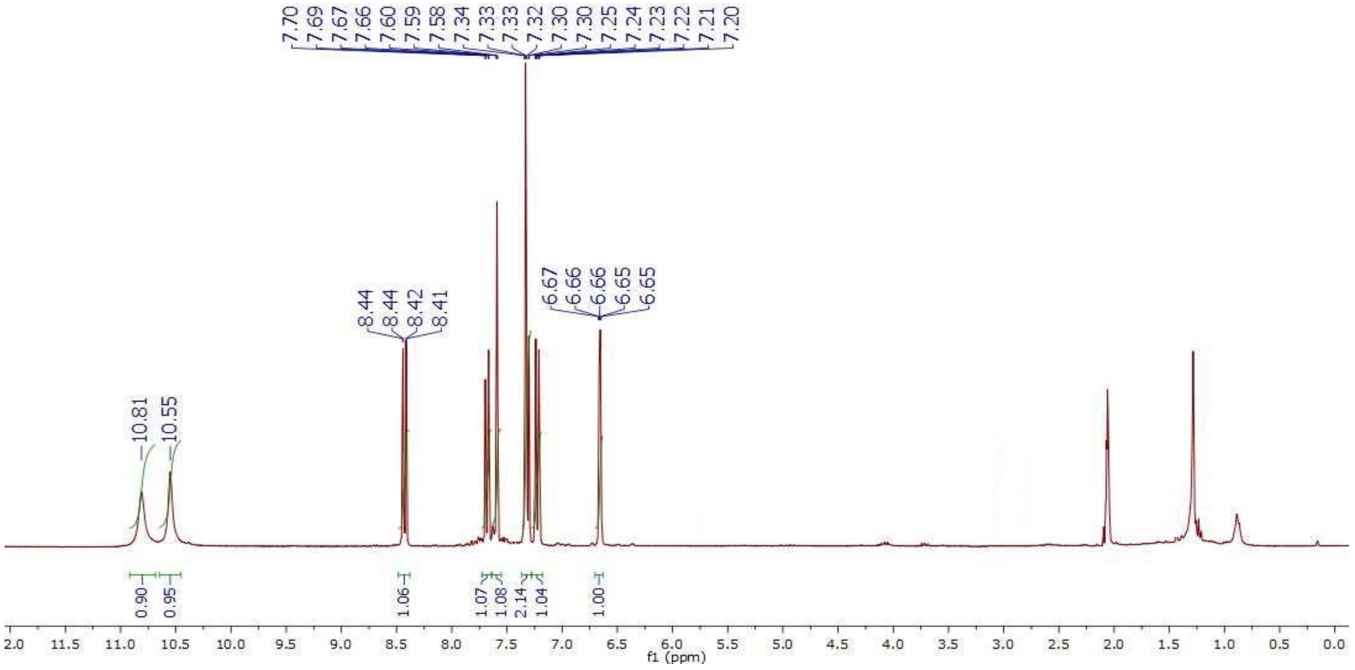
7.70
7.69
7.67
7.66
7.60
7.59
7.58
7.34
7.33
7.33
7.32
7.30
7.30
7.25
7.24
7.23
7.22
7.21
7.20
6.67
6.66
6.66
6.65
6.65
8.44
8.44
8.42
8.41
10.81
10.55
0.90
0.95
1.06
1.07
1.08
2.14
1.04
1.00
f1 (ppm)

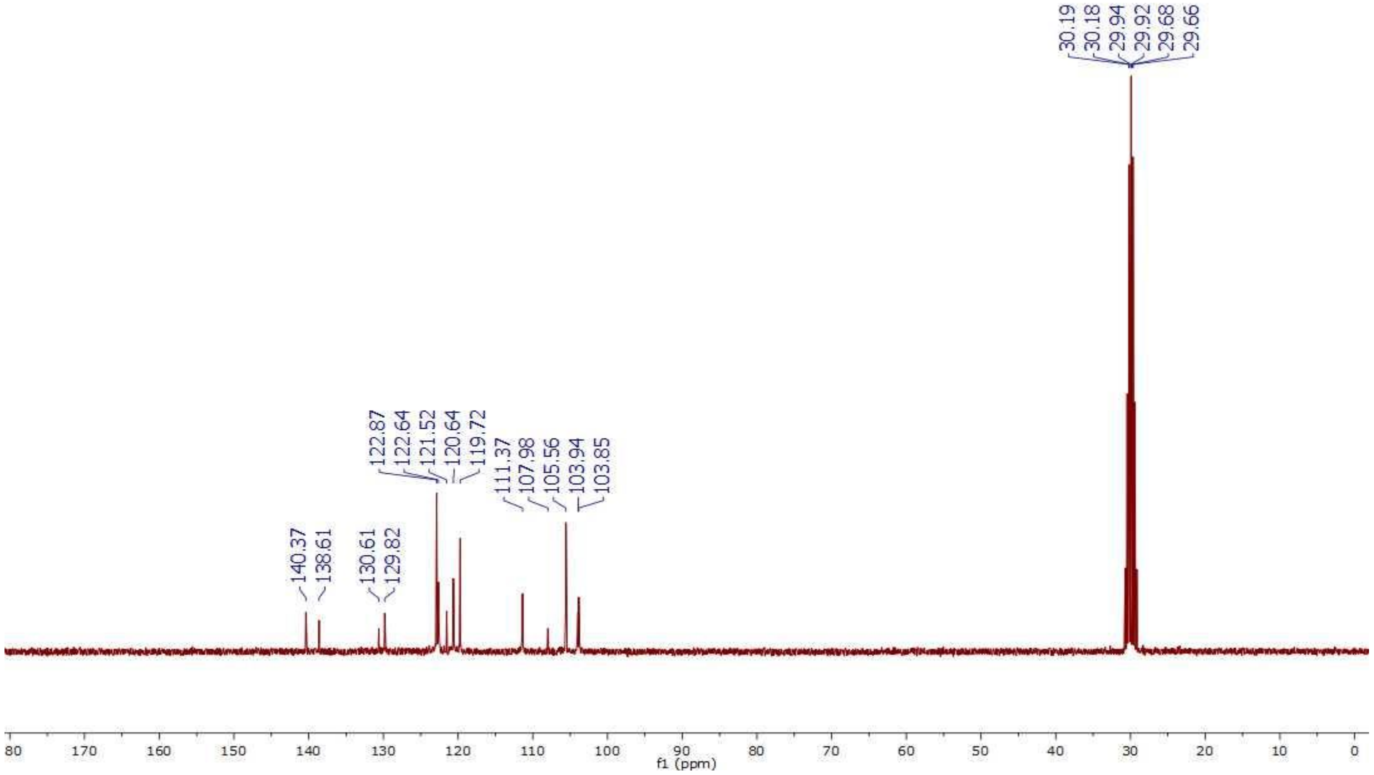
30.19
30.18
29.94
29.92
29.68
29.66
122.87
122.64
121.52
120.64
119.72
111.37
107.98
105.56
103.94
103.85
140.37
138.61
130.61
129.82
f1 (ppm)

10g

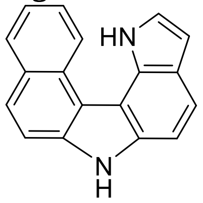
HN
N
H

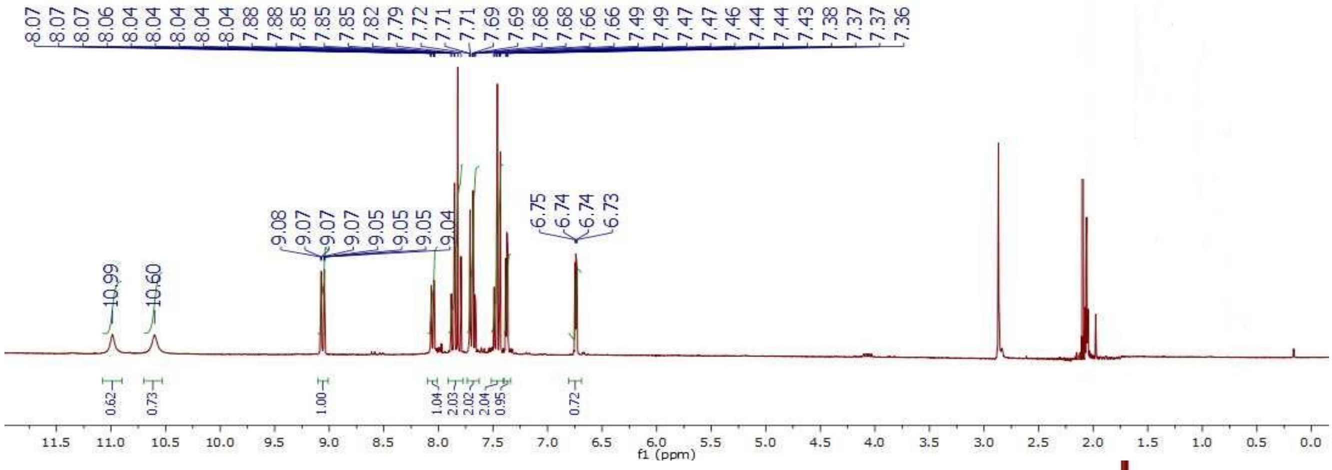
8.07 8.07 8.07 8.06 8.04 8.04 8.04 8.04 7.88 7.88 7.85 7.85 7.85 7.82 7.79 7.72 7.71 7.71 7.69 7.69 7.68 7.68 7.66 7.66 7.49 7.49 7.47 7.47 7.46 7.44 7.44 7.43 7.38 7.37 7.37 7.36
9.08 9.07 9.07 9.07 9.05 9.05 9.05 9.04
6.75 6.74 6.74 6.73
10.99
10.60
0.62 0.73 1.00 1.04 2.03 2.02 2.04 0.95 0.72
11.5 11.0 10.5 10.0 9.5 9.0 8.5 8.0 7.5 7.0 6.5 6.0 5.5 5.0 4.5 4.0 3.5 3.0 2.5 2.0 1.5 1.0 0.5 0.0
f1 (ppm)

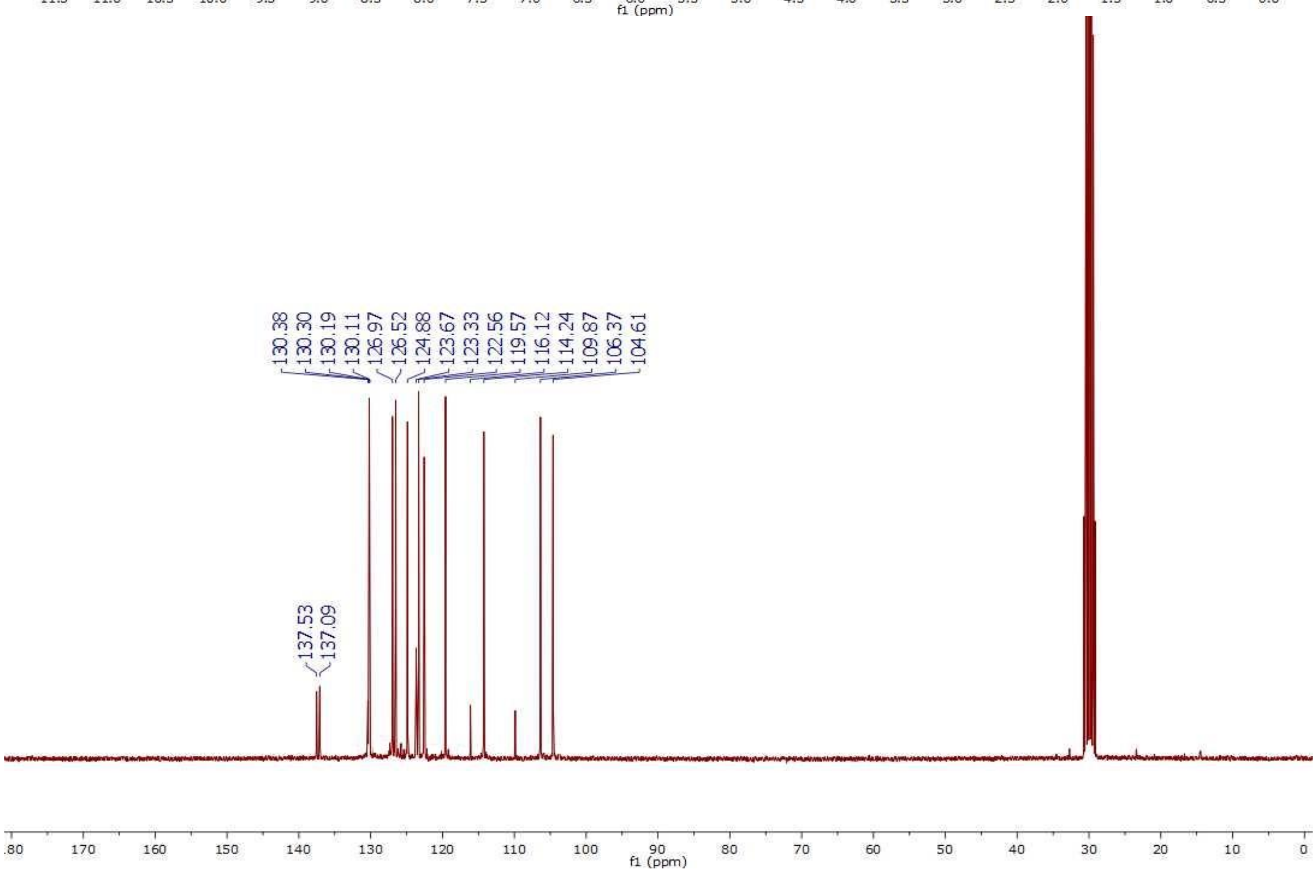
130.38 130.30 130.19 130.11 126.97 126.52 124.88 123.67 123.33 122.56 119.57 116.12 114.24 109.87 106.37 104.61
137.53 137.09
80 170 160 150 140 130 120 110 100 90 80 70 60 50 40 30 20 10 0
f1 (ppm)

10h

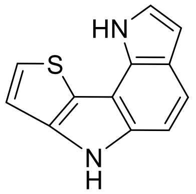
HN
S
N
H

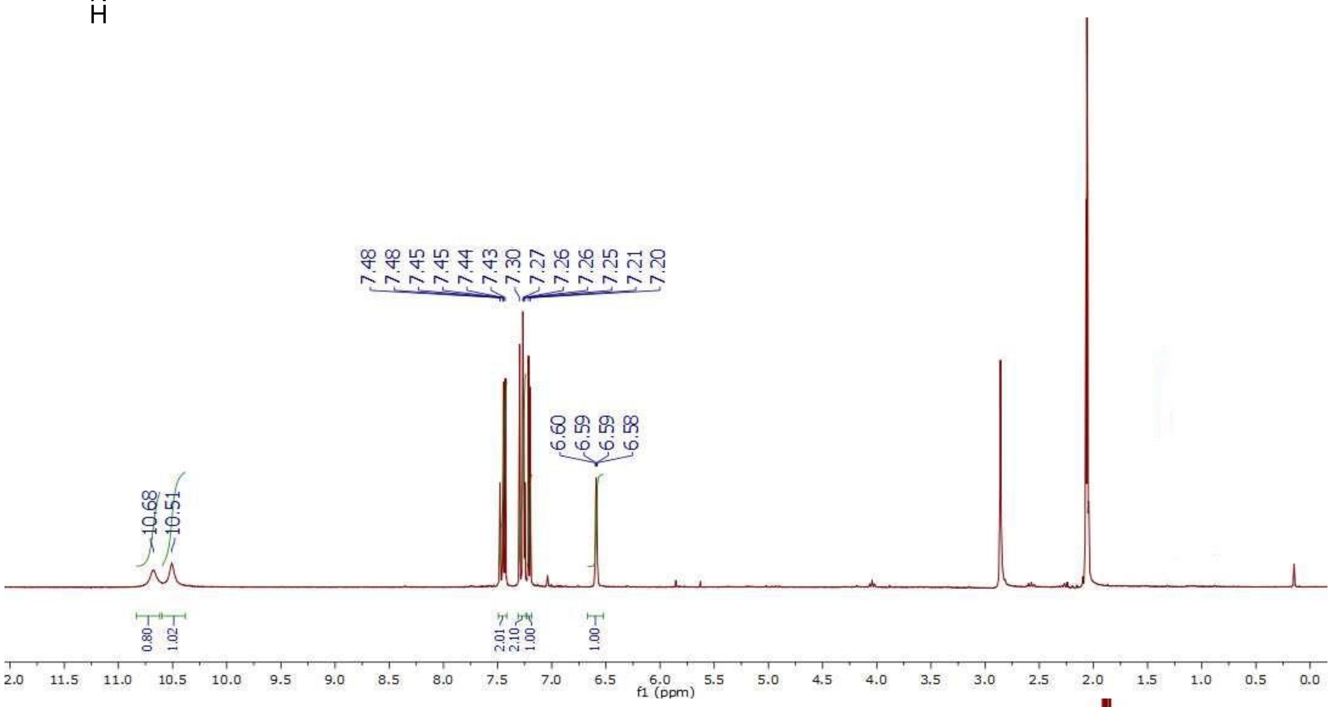
7.48
7.48
7.45
7.45
7.44
7.43
7.30
7.27
7.26
7.26
7.25
7.21
7.20
6.60
6.59
6.59
6.58
10.68
10.51
0.80
1.02
2.01
2.10
1.00
1.00
f1 (ppm)

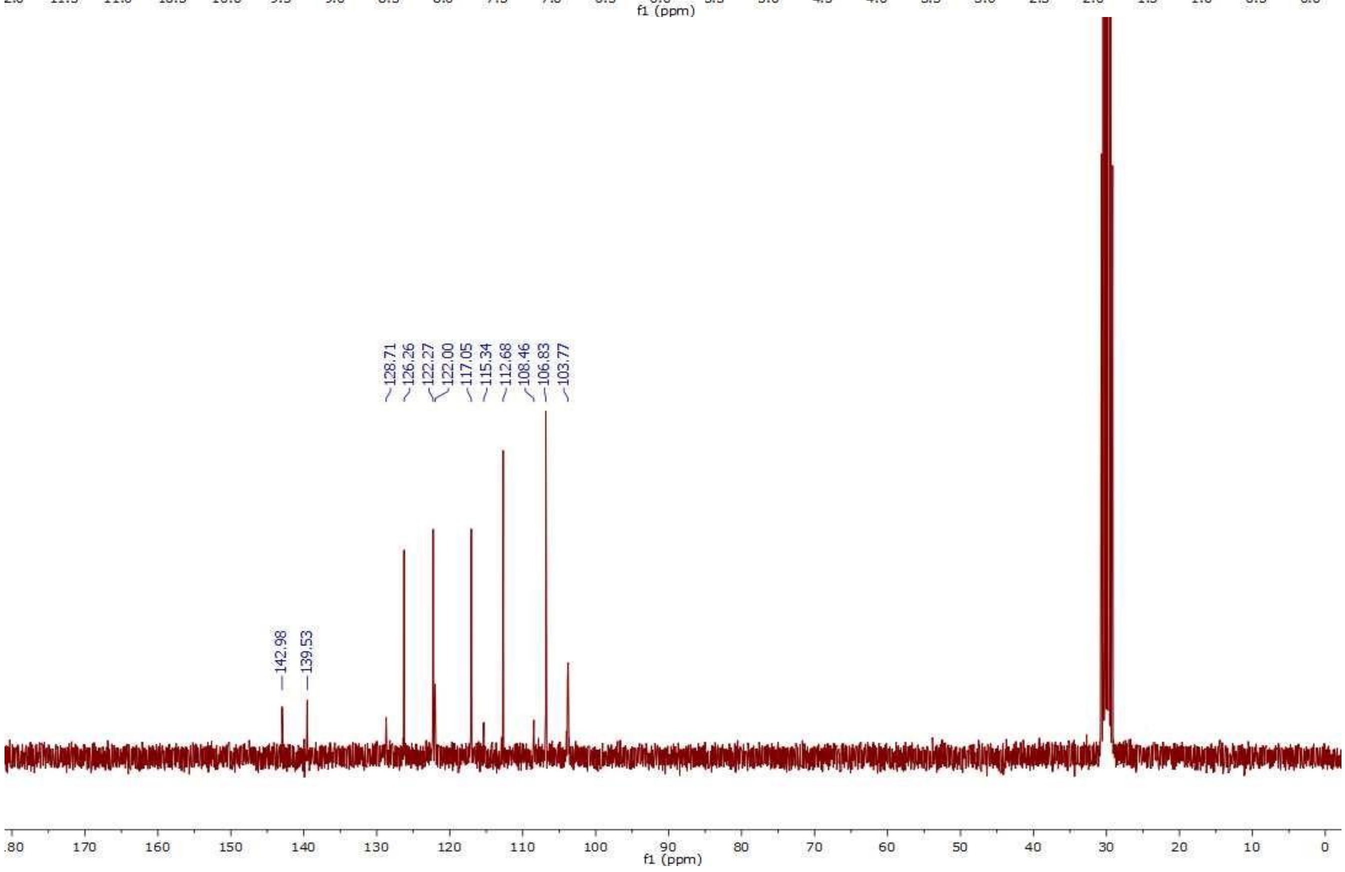
128.71
126.26
122.27
122.00
117.05
115.34
112.68
108.46
106.83
103.77
142.98
139.53
f1 (ppm)

11a

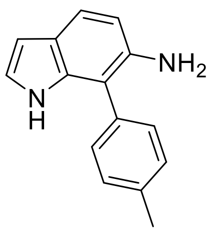

NH2
N
H

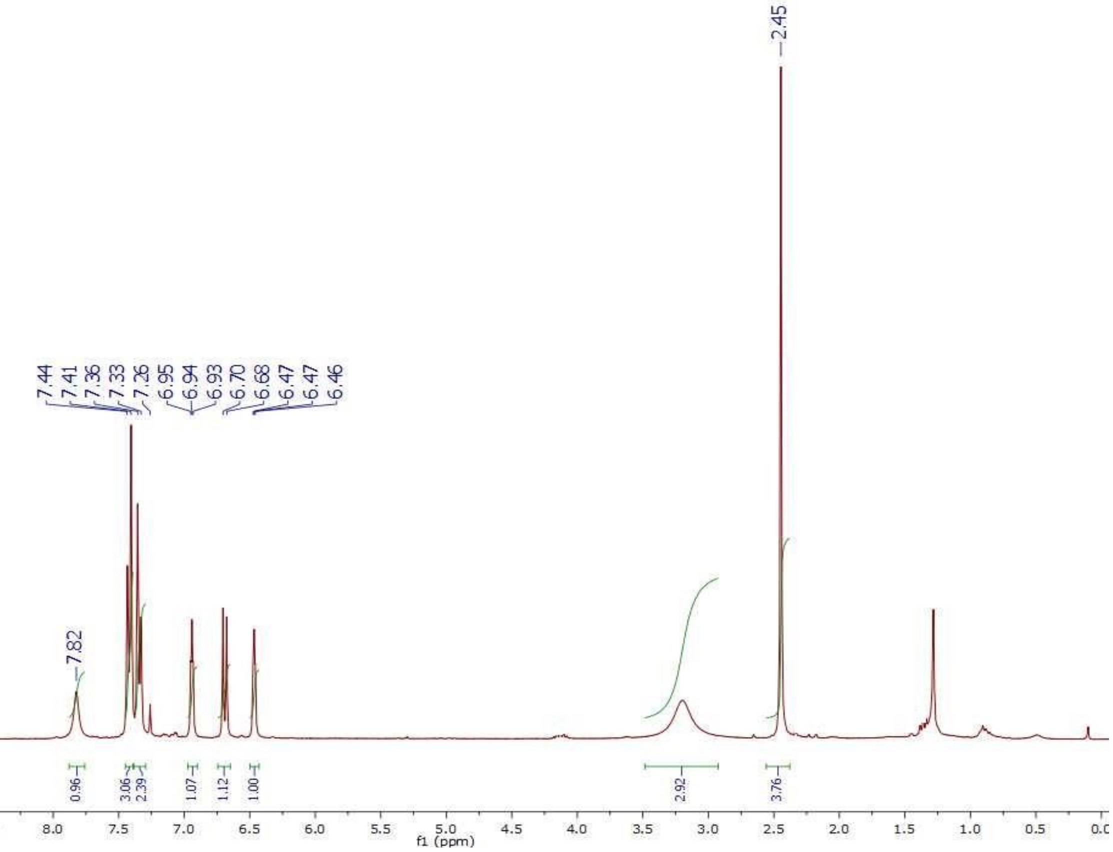

2.45
7.44
7.41
7.36
7.33
7.26
6.95
6.94
6.93
6.70
6.68
6.47
6.47
6.46
7.82
0.96
3.06
2.39
1.07
1.12
1.00
2.92
3.76
f1 (ppm)

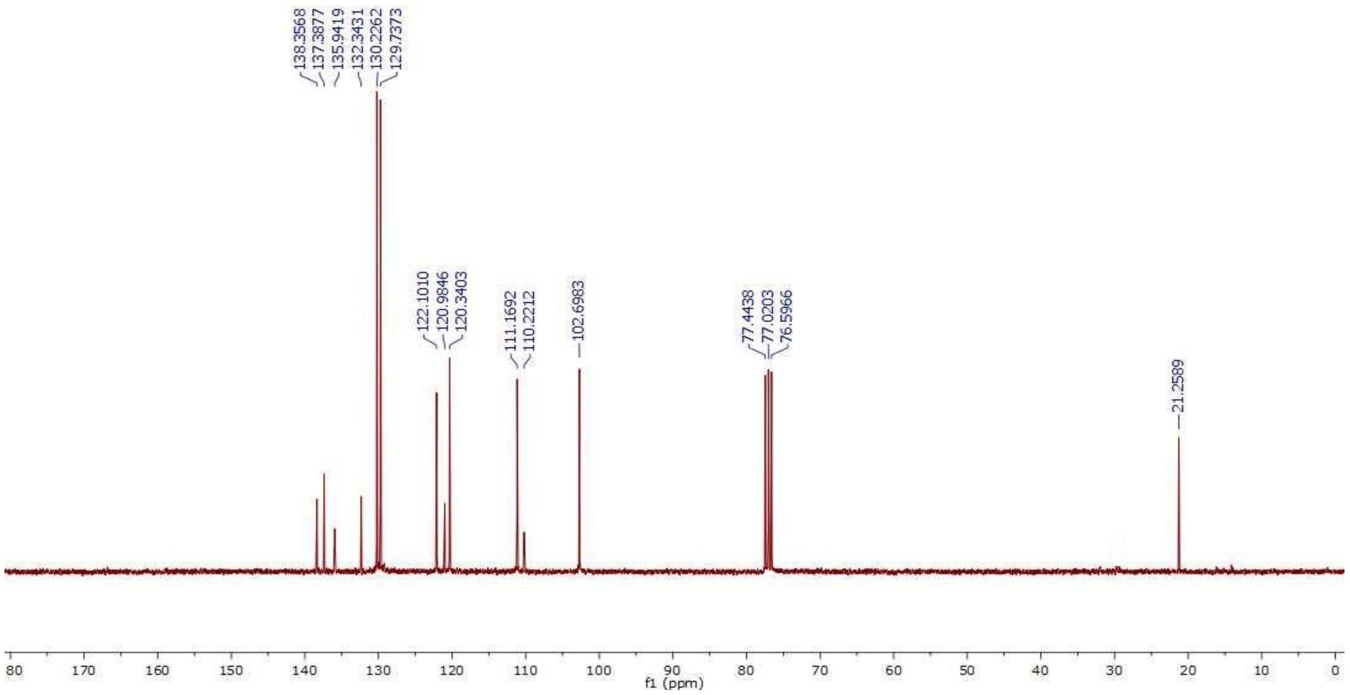

138.3568
137.3877
135.9419
132.3431
130.2262
129.7373
122.1010
120.9846
120.3403
111.1692
110.2212
102.6983
77.4438
77.0203
76.5966
21.2589
f1 (ppm)
